# Ring‐Opening Regio‐, Diastereo‐, and Enantioselective 1,3‐Chlorochalcogenation of Cyclopropyl Carbaldehydes

**DOI:** 10.1002/chem.201605265

**Published:** 2016-11-29

**Authors:** Jan Wallbaum, Lennart K. B. Garve, Peter G. Jones, Daniel B. Werz

**Affiliations:** ^1^ Institute of Organic Chemistry Technische Universität Braunschweig Hagenring 30 38106 Braunschweig Germany; ^2^ Institute of Inorganic and Analytical Chemistry Technische Universität Braunschweig Hagenring 30 38106 Braunschweig Germany

**Keywords:** 1,3-bisfunctionalization, cyclopropane, enantioselectivity, organocatalysis, sulfur

## Abstract

*meso*‐Cyclopropyl carbaldehydes are treated in the presence of an organocatalyst with sulfenyl and selenyl chlorides to afford 1,3‐chlorochalcogenated products. The transformation is achieved by a merged iminium–enamine activation. The enantioselective desymmetrization reaction, leading to three adjacent stereocenters, furnished the target products in complete regioselectivity and moderate to high diastereo‐ and enantioselectivities (d.r. up to 15:1 and e.r. up to 93:7).

Because of their high strain energy (about 27.5 kcal mol^−1^)^1^ cyclopropanes have been used in recent years as starting materials for the 1,3‐bisfunctionalization of aliphatic chains under ring‐opening conditions. Recently, the Szabó group generated 1,3‐difluorides from 1,1‐disubstituted cyclopropanes,^2^, ^3^ whereas our group synthesized 1,3‐dichlorides from donor‐acceptor cyclopropanes.^4^, ^5^ Sparr and Gilmour reported the use of aldehyde‐functionalized cyclopropanes for an asymmetric 1,3‐dichlorination,^6^ making use of a merged iminium–enamine activation (Scheme 1).^7^, ^8^, ^9^ They cleverly exploited the intermediate formation of iminium species by secondary amine catalysts, leading, after the attack of a chloride and opening of the three‐membered ring, to a formal umpolung of the γ‐position. The emerging enamine was intercepted by “Cl^+^” resulting in a formal 1,3‐addition of Cl_2_ across the C−C bond adjacent to the carbonyl group (Scheme 1).^6^ In contrast to numerous papers dealing with asymmetric 1,2‐bisfunctionalizations employing two different residues,^7^ such regio‐, diastereo‐, and enantioselective 1,3‐bisfunctionalizations have not been reported.

**Scheme 1:**
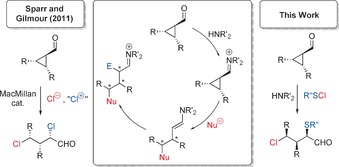
Asymmetric 1,3‐dichlorination by Sparr and Gilmour (left). General mechanistic hypothesis for the 1,3‐bisfunctionalization using cyclopropanes (middle). Envisioned asymmetric 1,3‐chlorosulfenation (right).

On the basis of these results we were keen to investigate whether such asymmetric regioselective 1,3‐bisfunctionalizations are also feasible using different substituents. We chose sulfenyl chlorides as reactants to add across the C−C bond in the cyclopropane;^10^, ^11^ because of the highly polarized S−Cl bond we anticipated that sulfur would act as the electrophilic component, with chlorine as the nucleophilic counterpart (Scheme 1, right).^12^

To test our notion, achiral *meso*‐cyclopropyl carbaldehyde **1 a** and *p*‐tolylsulfenyl chloride **2 a** were chosen as substrates for the optimization.^13^ As starting point we chose the first generation MacMillan catalyst^14^ using different counterions, which provided the desired product, but without any enantioinduction (Table 1, Entries 1 and 2).

**Table 1:** Optimization of the enantioselective 1,3‐chlorosulfenation of *meso*‐cyclopropyl carbaldehyde (**1 a**).^[a]^

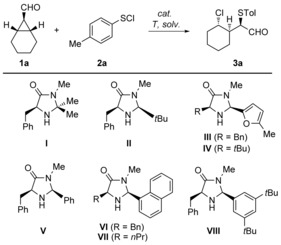						
Entry	Cat.	Solvent	*T* [°C]	Yield^[b]^ [%]	d.r.^[c]^	e.r.^[d]^
1	**I⋅TFA**	CDCl_3_	rt	56	4.0:1	50:50
2	**I⋅HCl**	CDCl_3_	rt	58	3.2:1	53:47
3	**II**	CDCl_3_	rt	57	12:1	50:50
4	**III**	DCM	rt	63	3.4:1	69:31
5	**III**	DCM	0	63	4.5:1	82:18
6	**IV**	DCM	0	72	4.0:1	63:37
7	**V**	DCM	0	67	4.0:1	78:22
8	**VI**	DCM	0	71	4.5:1	83:17
9	**VII**	DCM	0	76	7.0:1	62:38
10	**VI⋅TFA**	DCM	0	66	4.2:1	88:12
11	**VI⋅DCA**	DCM	0	67	6.5:1	89:11
12	**VI⋅DCA**	DME	0	60	6.3:1	90:10
13	**VI⋅DCA**	EtOAc	0	59	4.5:1	91:9
14	**VI⋅DCA**	EtOAc	‐4	68	4.6:1	**93:7** 18
15^[e]^	**VI⋅DCA**	EtOAc	‐4	67	4.5:1	91:9
16	**VI⋅DCA**	EtOAc	‐8	65	5.6:1	88:12
17	**VIII**	DCM	rt	**84**	**>20:1**	60:40

[a] Reaction conditions: **1 a** (100 μmol), **2 a** (120 μmol), cat. (20 mol %), solv. (1.0 mL), reaction time 15 min to 24 h under Ar; subsequent reduction with NaBH_4_ (500 μmol) in EtOH (1.0 mL) for 15 min at given temperature. [b] Isolated yield over 2 steps. [c] Determined after column chromatography by ^1^H‐NMR spectroscopy. [d] Determined as the corresponding crude Mosher ester by ^19^F‐NMR spectroscopy. [e] Air atmosphere and damp EtOAc used. TFA=trifluoroacetic acid. DCA=dichloroacetic acid. DME=dimethoxyethane.

Use of second generation MacMillan catalysts only provided a better diastereoselectivity (Table 1, Entry 3).^15^ By changing the catalyst to **III**,^16^ the solvent to DCM, and lowering the temperature to 0 °C, the enantiomeric ratio was substantially increased (Table 1, Entries 4 and 5). Replacement of the benzyl group with a *tert*‐butyl moiety (catalyst **IV**) resulted in a lowered e.r. (Table 1, Entry 6). While the less sterically demanding catalyst **V** 17 afforded also lower e.r., **VI** showed a slightly better selectivity (Table 1, Entries 7 and 8). Exchange of the benzyl for a less bulky propyl group resulted in a decrease of the e.r., thus we continued with catalyst **VI** (Table 1, Entry 9).

With the optimized results in hand, a variety of sulfenyl chlorides and phenylselenyl chloride were subjected to the reaction (Table 2). Various aryl sulfenyl chlorides were utilized (Table 2, Entries 1–4); electron‐withdrawing and also electron‐donating substituents were tolerated, affording the desired products in moderate to good yields (61–84 %). The diastereoselectivity was improved by using electron‐rich sulfenyl chlorides (**3 b**), while electron‐poor aryl units led to lower selectivities (**3 d**, **3 e**). In all cases a slight decrease of the enantiomeric ratio was observed compared to the optimized model system.

**Table 2:** Scope using cyclopropyl carbaldehyde (**1 a**).^[a]^

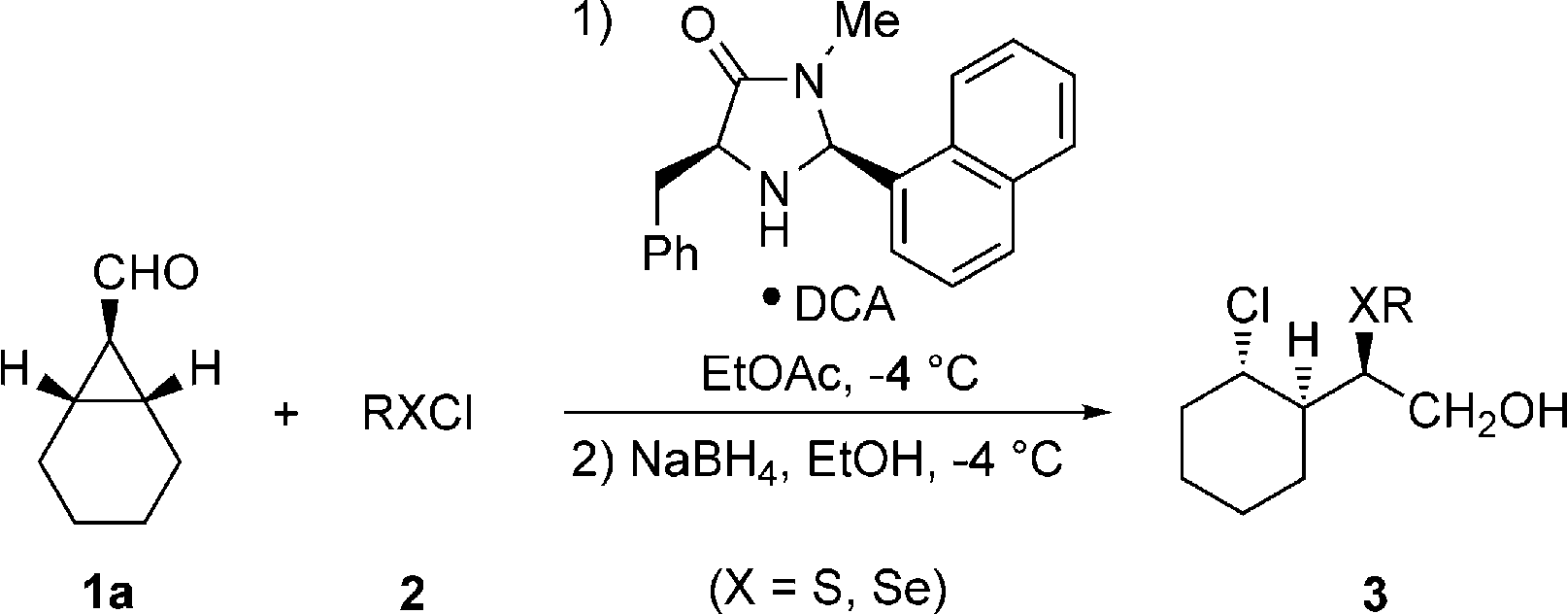						
Entry	Substrate **2**	Product		Yield^[b]^ [%]	d.r.^[c]^	e.r.^[d]^
1	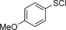	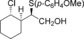	**3 b**	63	5.7:1	87:13
2	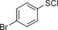	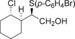	**3 c**	84	3.5:1	90:10
3^[e]^	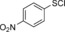	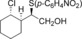	**3 d**	62	3.1:1	85:15
4	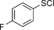	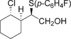	**3 e**	61	2.5:1	90:10
5	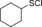	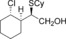	**3 f**	42	15:1	83:17
6^[f]^	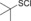	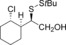	**3 g**	61	4.2:1	75:25
7^[g]^	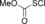	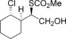	**3 h**	56	4.2:1	83:17
8	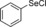	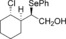	**3 i**	72	5.7:1	79:21

[a] Reaction conditions: **1 a** (100 μmol), **2** (120 μmol), **VI⋅DCA** (20 mol %), EtOAc (1.0 mL), reaction time 90 min to 120 min; subsequent reduction with NaBH_4_ (500 μmol) in EtOH (1.0 mL) for 15 min at −4 °C. [b] Isolated yield over 2 steps. [c] Determined after column chromatography by ^1^H‐NMR spectroscopy. [d] Determined as the corresponding crude Mosher ester by ^19^F‐NMR spectroscopy. [e] 200 μmol scale. [f] 2.0 equiv of **2 g** were used. [g] Reduction with NaBH_4_ (1.5 equiv) for 60 min.

The use of sterically demanding alkyl sulfenyl chlorides (Table 2, Entries 5 and 6) was possible. In the case of the cyclohexyl substituent, a significantly higher diastereomeric ratio was observed, while yield and enantiomeric ratio decreased. In the case of *tert*‐butyl sulfenyl chloride, the system avoided the steric hindrance between the bulky *tert*‐butyl moiety and the aliphatic chain by forming the disulfide **3 g**, and thus two equivalents of the sulfenyl chloride had to be used; lower amounts resulted in the same product and selectivity, but with lower yield. With primary alkyl sulfenyl chlorides the desired products were not observed. Additionally, commercially available methoxycarbonyl sulfenyl chloride and phenylselenyl chloride were tested as reagents. The products **3 h** and **3 i** were obtained, respectively, in good yield with acceptable diastereo‐ and enantioselectivity. The use of analogous sulfenyl and selenyl bromides showed no conversion of **1 a**, probably because the less polarized S−Br and Se−Br bonds are not sufficiently reactive.

After evaluating the scope with respect to **1 a**, we varied the substituents of the cyclopropyl carbaldehyde to determine limitations of the reaction (Table 3). Exchanging the cyclohexyl ring for two ethyl moieties resulted in a prolonged reaction time, probably because of the loss of ring strain and increased steric hindrance. The diastereoselectivity was completely lost, while the e.r. showed only a slight decrease for the analogous diastereomer **4 b**. In this case the undesired diastereomer **4 b′** was also isolated, still showing an e.r. of 74:26. Determination of the configuration by NOESY revealed that the stereocenter next to the hydroxymethyl group is inverted. The other two stereocenters are built up in a catalyst‐controlled fashion and are defined during the cyclopropane ring‐opening step after iminium activation. Replacement of the ethyl groups by phenyl groups (Table 3, Entry 2) yielded no reactivity of the substrate at the optimized conditions. The reaction temperature had to be increased to ambient temperature and the catalyst was changed to **III** with 5‐methylfuryl as substituent R^1^. These changes were necessary to overcome the larger steric hindrance of the phenyl rings compared to the ethyl moieties, providing the major diastereomer **4 c** in reasonable d.r. and e.r.

**Table 3:** Scope and limitations using varying cyclopropyl carbaldehydes **1**.^[a]^

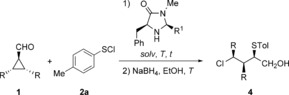						
Entry	Substrate **1**	Product		Yield^[b]^ [%]	d.r.^[c]^	e.r.^[d]^
1^[e]^	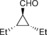	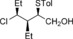	**4 b**	63	1:1	88:12
		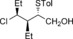	**4 b′**			74:26
2^[f]^	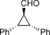	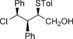	**4 c**	70	5.2:1	81:19
3^[g]^	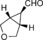	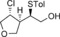	**4 d**	65	2.1:1	71:29
4^[h]^	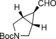	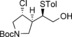	**4 e**	48	1.6:1	50:50

[a] Reaction conditions: **1** (100 μmol), **2 a** (120 μmol), cat. (20 mol %), solv. (1.0 mL); subsequent reduction with NaBH_4_ (500 μmol) in EtOH (1.0 mL) for 15 min at given temperature. [b] Isolated yield over 2 steps. [c] Determined after column chromatography by ^1^H‐NMR spectroscopy. [d] Determined as the corresponding crude Mosher ester by ^19^F‐NMR spectroscopy. [e] 200 μmol scale, cat. **VI⋅DCA**, EtOAc, −4 °C, 20 h; reduction time 30 min. [f] Cat. **III**, EtOAc, rt, 5 h. [g] Cat. **V⋅DCA**, DME, rt, 72 h. [h] Cat. **VI**, DME (2.0 mL), rt, 72 h.

Entry 3 and especially Entry 4 (Table 3) reveal the limitations of the transformation. Because of the increased polarity of the substrates **1 d** and **1 e**, DME had to be used as solvent to avoid precipitation of intermediates. The reaction time was increased to 72 h at ambient temperature. In the case of the substrate **1 d**, a moderate selectivity was achieved using the catalyst **V⋅DCA**, while utilization of the Boc‐protected pyrrolidine cyclopropyl carbaldehyde **1 e** as substrate gave the desired product **4 e** with almost no selectivity regarding d.r. and e.r. Exchange of the Boc‐ for benzyl‐ or tosyl‐protected pyrrolidine led to no conversion of the substrates. The preliminary studies using these polar substrates demonstrate that they can, indeed, be converted to the resulting 1,3‐chlorochalcogenated products, but to obtain higher selectivities different catalytic systems need to be examined.

The reaction mechanism consists of a merged iminium–enamine activation (Scheme 2). The initial step is the formation of the iminium ion **VIb**, which is then attacked at the 3‐position by the chloride from the sulfenyl chloride, leading to the enamine complex **VIc**, while releasing the positively charged sulfenylium ion.

**Scheme 2:**
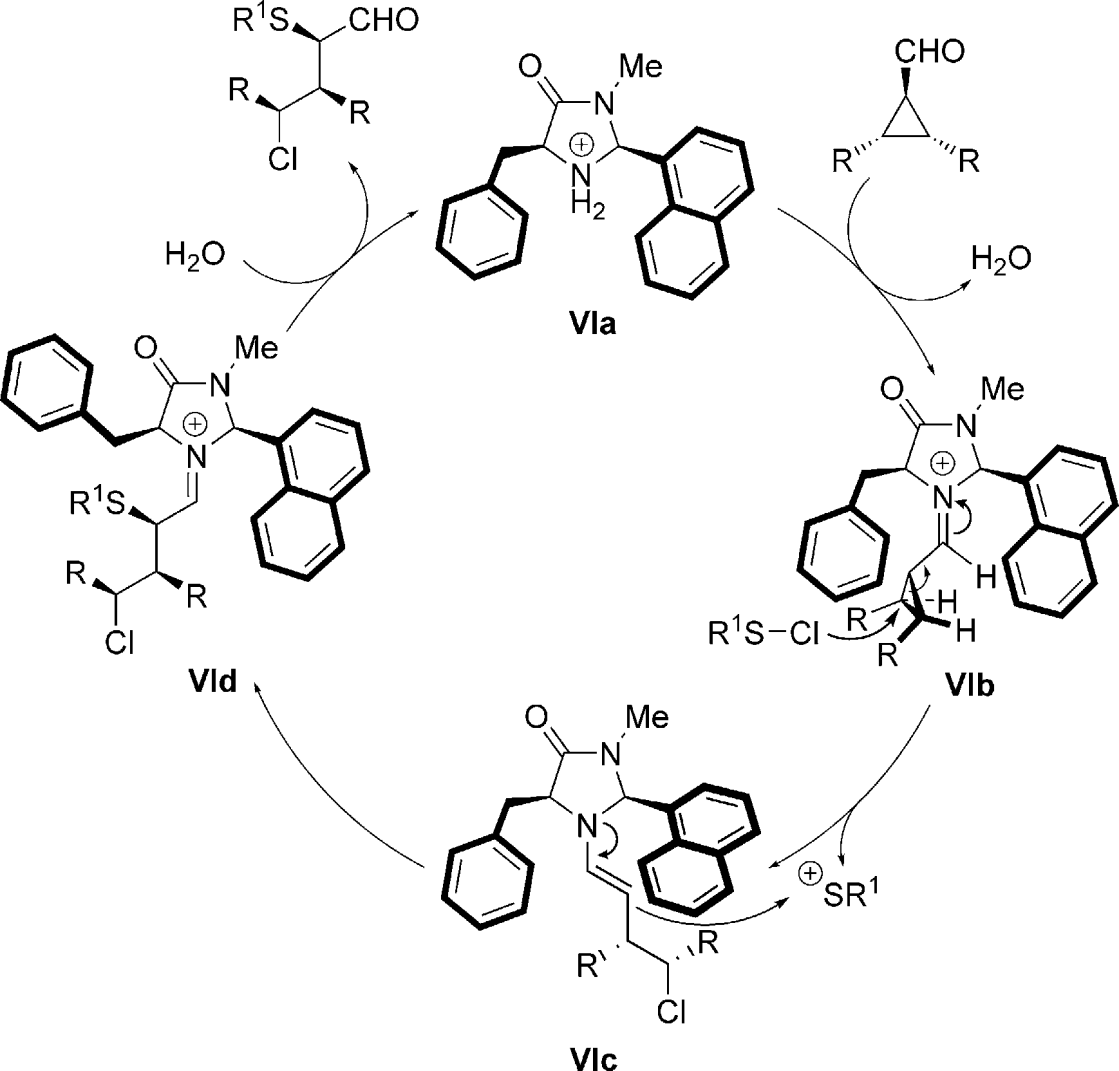
Proposed catalytic cycle of the enantioselective 1,3‐chlorochalcogenation.

The benzyl moiety that shields the top face seems to be crucial for enantioselectivity, which is determined in this step. The subsequent attack of the emerging enamine at the cationic RS forms the iminium complex **VId**. This last step is responsible for the diastereomeric ratio of the transformation. As demonstrated in Table 1 (Entries 3 and 17) and Table 2 (Entry 5), the larger the substituent, the greater the steric repulsion and the better the diastereomeric ratio. Final hydrolysis releases the product and regenerates the active catalyst **VIa**.

To confirm the relative and absolute configuration of the three contiguous stereocenters, **3 a** was converted to the corresponding ester **5** with ferrocenecarboxylic acid chloride^19^ and 4‐DMAP. X‐ray crystallography revealed the (2*R*,3*R*,4*S*)‐enantiomer as major product of the reaction (Figure 1).^20^, ^21^

**Figure 1:**
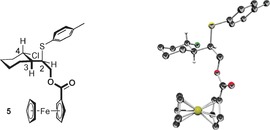
Molecular structure of the ferrocenyl ester **5**. Thermal ellipsoids are given at 50 % probability level.^21^ Hydrogen atoms at all nonstereogenic centers are omitted for clarity.

In summary, we have developed the first 1,3‐chlorochalcogenation of cyclopropyl carbaldehydes using highly polarized sulfenyl and selenyl chlorides. The key to success was the application of iminium–enamine catalysis to aldehyde‐substituted cyclopropanes, paving the way to a ring‐opening 1,3‐bisfunctionalization using a nucleophilic and an electrophilic component. Chiral imidazolidinone organocatalysts delivered the desired target products in diastereomeric ratios of up to 15:1 and enantioselectivities of up to 93:7.

## Supporting information

As a service to our authors and readers, this journal provides supporting information supplied by the authors. Such materials are peer reviewed and may be re‐organized for online delivery, but are not copy‐edited or typeset. Technical support issues arising from supporting information (other than missing files) should be addressed to the authors.

Supplementary
