# Supplementary material for: Ring‐Opening Regio‐, Diastereo‐, and Enantioselective 1,3‐Chlorochalcogenation of Cyclopropyl Carbaldehydes
Source: Chemistry. 2016 Nov 29;22(52):18756–9. doi: 10.1002/chem.201605265 (PMC6680189; doi:10.1002/chem.201605265)
Supplement: Supplementary file 1 — Supplementary [file CHEM-22-18756-s001.pdf]

CHEMISTRY
A European Journal

Supporting Information

Ring-Opening Regio-, Diastereo-, and Enantioselective 1,3-Chlorochalcogenation of Cyclopropyl Carbaldehydes

Jan Wallbaum,[a] Lennart K. B. Garve,[a] Peter G. Jones,[b] and Daniel B. Werz*[a]

chem_201605265_sm_miscellaneous_information.pdf

Table of Contents

General Experimental S2

Experimental Section S3

^{1}H-, ^{13}C- and ^{19}F-NMR Spectra of New Compounds S37

Crystal Structure Determination of **5** S59

General Experimental

All solvents were distilled before use unless otherwise stated. Dichloromethane (CH_2Cl_2) was distilled from $CaCl_2$ under an argon atmosphere. Dimethoxyethane (DME) and tetrahydrofuran (THF) were distilled over sodium and benzophenone under an argon atmosphere. Dry ethylacetat (EtOAc) over molecular sieves was bought from *Acros* and used without further purification. The Mosher Ester Acid Chloride was bought from *Sigma Aldrich* in a purity of ≥ 99.0%. Air and moisture sensitive reactions were carried out in oven-dried or flame-dried glassware, septum-capped under atmospheric pressure of argon. Commercially available compounds were used without further purification unless otherwise stated.

Proton (^{1}H) and carbon (^{13}C) NMR spectra were recorded on a 300, 400 or 600 MHz instrument using the residual signals from $CHCl_3$, δ = 7.26 ppm and δ = 77.0 ppm, using TMS as internal reference for ^{1}H and ^{13}C chemical shifts, respectively. Assignments of the respective signals and the stereochemistry were made by combination of H,H-COSY, HSQC, HMBC and NOESY experiments. ESI-HRMS mass spectrometry was carried out on a FTICR instrument. IR spectra were measured on an ATR spectrometer. UV spectra were measured with a common photometer. Optical rotation was measured on a common polarimeter.

General Procedure 1 (GP1): A *Schlenk* flask was charged with a magnetic stirrer and powdered 4 Å molecular sieves (20 mg/mL) and flame dried for 10 minutes under high vacuum. After cooling to ambient temperature methylamide (1.0 eq.) was added and the flask subjected to the glove box. After addition of $Yb(OTf)_3$ (10 mol%) the flask was removed from the glovebox and THF (4.0 mL/mmol) and corresponding aldehyde (1.1 to 2.0 eq.) were added subsequently. The resulting mixture was stirred at ambient temperature for given time. The suspension was filtrated over celite and the solvent removed *in vacuo*.

(2*R*,5*S*)-(–)-5-*tert*-butyl-3-methyl-2-(5-methylanfuran-2-yl)-4-imidazolidinone (IV')

(2*S*,5*S*)-(–)-5-*tert*-butyl-3-methyl-2-(5-methylanfuran-2-yl)-4-imidazolidinone (IV)

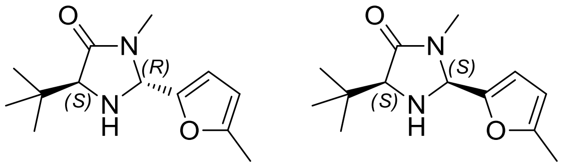

(*S*)-*Tert*-leucinmethylamide (1.44 g, 10.0 mmol, 1.0 eq.), 5-methylfufural (1.21g, 11.0 mmol, 1.09 mL, 1.1 eq.), 4 Å molecular sieves (800 mg) and $Yb(OTf)_3$ (621 mg, 1.00 µmol, 10 mol%) in THF (40 mL) were reacted according to GP1. Silica gel column chromatography (*n*-pentane:EtOAc = 10:1 → 2:1) gave the faster eluting (*S*,*R*)-diastereomere **IV'** (805 mg, 3.41 mmol, 34%) and the desired (*S*,*S*)-diastereomere **IV** (350 mg, 1.48 mmol, 15%) as white solids.

2*R*,5*S*-diastereomere **IV'**:

^{1}H-NMR (600 MHz, $CDCl_3$): δ = 1.04 (s, 9 H), 2.27 (t, J = 0.9 Hz, 3 H), 2.67 (s, 3 H), 3.46 (dd, J = 1.8, 0.9 Hz, 1 H), 5.25 (d, J = 1.8 Hz, 1 H), 5.93 (dq, J = 3.1, 0.9 Hz, 1 H), 6.28 (d, J = 3.1 Hz, 1 H).

^{13}C-NMR (150 MHz, $CDCl_3$): δ = 13.6, 26.1, 26.6, 35.1, 66.4, 71.1, 106.3, 110.6, 149.5, 153.3, 173.2.

$\boldsymbol{\alpha_D^{24.0}}$ ($CHCl_3$, c = 1.0) = –19.0°.

IR (ATR) $\tilde{\nu}$ (cm^{-1}) = 3359, 2952, 1675, 1425, 1272.

MS (ESI): *m*/*z* = 259.1 $[M+Na]^+$, 495.2 $[2M+Na]^+$.

$C_{13}H_{20}N_2O$ calcd.: 295.1417

found: 295.1413, $[M+Na]^+$ (ESI-HRMS).

2*S*,5*S*-diastereomere **IV**:

^{1}H-NMR (600 MHz, $CDCl_3$): δ = 1.11 (s, 9 H), 2.30 (dd, *J* = 1.1, 0.4 Hz, 3 H), 2.65 (d, *J* = 0.7 Hz, 3 H), 3.30 (dd, *J* = 1.8, 0.7 Hz, 1H), 5.20 (d, *J* = 1.8 Hz, 1H), 5.97 (dq, *J* = 3.1, 1.1 Hz, 1H), 6.36 (dd, *J* = 3.1, 0.4 Hz, 1 H).

^{13}C-NMR (75 MHz, $CDCl_3$): δ = 13.6, 26.1, 26.9, 33.6, 67.0, 69.9, 106.4, 110.9, 149.2, 153.4, 173.6.

$\boldsymbol{\alpha_D^{24.0}}$ ($CHCl_3$, c = 1.0) = –43.0°.

IR (ATR) $\tilde{\nu}$ (cm^{-1}) = 3343, 2952, 1664, 1416, 1022.

(2*R*,5*S*)-(–)-5-Benzyl-3-methyl-2-(1-naphthyl)-4-imidazolidinone (VI')

(2*S*,5*S*)-(–)-5-Benzyl-3-methyl-2-(1-naphthyl)-4-imidazolidinone (VI)

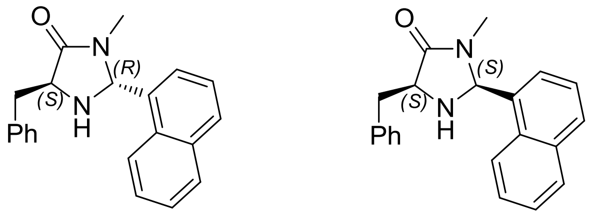

Recrystallized (*S*)-phenylalaninmethylamide (1.78 g, 10.0 mmol, 1.0 eq.), 1-naphthaladehyde (3.12 g, 2.70 mL, 20.0 mmol, 2.0 eq.), 4 Å molecular sieves (800 mg) and $Yb(OTf)_3$ (624 mg, 1.00 mmol, 10 mol%) in THF (40 mL) were reacted according to GP1 for 20 h. Silica gel column chromatography gave the faster eluting 2*R*,5*S*-diasteroemere **VI'** (1.35 g, 4.27 mmol, 43%, eluent = DCM) and the 2*S*,5*S*-diastereomere **VI** (1.42 g, 4.49 mmol, 45%, eluent = Et_2O) as colorless oils. The corresponding acid adducts were prepared by adding 1.0 eq. of the acid to a solution of the imidazolidinone in DCM and subsequent evaporation of the solvent *in vacuo*.

2*R*,5*S*-diastereomere **VI'**:

^{1}H-NMR (200 MHz, $CDCl_3$): δ = 2.69 (s, 3 H), 3.00–3.21 (m, 2 H), 4.01 (dddd, *J* = 5.3, 4.5, 1.5, 0.7 Hz, 1 H), 5.48 (d, *J* = 1.5 Hz, 1 H), 7.21–7.61 (m, 9 H), 7.78–7.92 (m, 2 H), 7.94–8.02 (m, 1 H).

^{13}C-NMR (75 MHz, $CDCl_3$): δ = 28.0, 38.6, 59.8, 74.8, 122.5, 123.1, 125.3, 126.1, 126.7, 126.8, 128.3, 128.9, 129.5, 129.8, 130.9, 133.7, 134.1, 137.7, 175.3.

$\boldsymbol{\alpha_D^{24.0}}$ ($CHCl_3$, c = 1.0) = –15.0°.

IR (ATR) $\tilde{\nu}$ (cm^{-1}) = 3306, 2920, 1688, 1397, 1095.

MS (ESI): *m*/*z* = 339.1 $[M+Na]^+$, 655.3 $[2M+Na]^+$.

$C_{21}H_{20}N_2O$ calcd.: 339.1468

found: 339.1472, $[M+Na]^+$ (ESI-HRMS).

2*S*,5*S*-diastereomere **VI**:

^{1}H-NMR (200 MHz, $CDCl_3$): δ = 1.96 (br s, 1 H), 2.66 (s, 3 H), 3.13 (dd, *J* = 14.1, 5.2 Hz, 1 H), 3.28 (dd, *J* = 14.1, 5.2 Hz, 1 H), 3.96 (br s, 1 H), 5.94 (br s, 1 H), 6.82 (br s, 1 H), 7.07–7.40 (m, 6 H), 7.39–7.63 (m, 2 H), 7.75–7.95 (m, 2 H), 8.01 (br s, 1 H).

^{13}C-NMR (75 MHz, $CDCl_3$): δ = 27.9, 36.6, 60.8, 122.3, 125.4, 126.0, 126.8, 126.9, 128.7, 128.9, 129.7, 131.1, 133.9, 136.5, 175.1 (one aliphatic and two aromatic CH-signals as well as one aromatic C_q-signal are missing).

$\boldsymbol{\alpha_D^{24.0}}$ ($CHCl_3$, c = 1.0) = –18.0°.

IR (ATR) $\tilde{\nu}$ (cm^{-1}) = 3324, 2919, 1690, 1393, 1096.

(2*S*,5*S*)-(+)-5-Benzyl-3-methyl-2-propyl-4-imidazolidinone (VII)

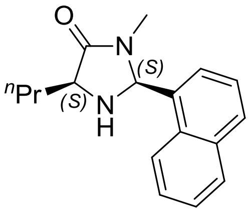

(*S*)-Norvalinmethylamide (521 mg, 4.00 µmol, 1.0 eq.), 1-naphthaldehyde (562 mg, 489 µL, 3.60 mmol, 0.9 eq.), 4 Å molecular sieves (480 mg) and $Yb(OTf)_3$ (248 mg, 400 µmol, 10 mol%) in THF (24 mL) were reacted according to GP1. Silica gel column chromatography (*n*-pentane:EtOAc = 4:1 → 1:1) gave the desired (*S,S*)-diastereomere **VII** (440 mg, 1.64 mmol, 46%) as pale yellow oil.

^{1}H-NMR (200 MHz, $CDCl_3$): δ = 0.94 (t, *J* = 7.2 Hz, 3 H), 1.34–1.71 (m, 3 H), 1.87 (br s, 1 H, N<u>H</u>), 1.89–2.13 (m, 1 H), 3.57–3.70 (m, 1 H), 6.01 (br s, 1 H), 7.40–7.61 (m, 4 H), 7.83–8.00 (m, 2 H), 8.15–8.29 (m, 1 H).

^{13}C-NMR (75 MHz, $CDCl_3$): δ = 13.9, 19.2, 28.1, 34.4, 59.8, 77.2, 122.6, 125.4, 126.2, 126.9, 129.0, 129.8, 131.2, 131.4, 133.7, 134.1, 176.5.

$\alpha_D^{24.0}$ ($CHCl_3$, c = 1.0) = +89.0°.

IR (ATR) $\tilde{\nu}$ (cm^{-1}) = 3321, 2957, 1689, 1396, 1322.

MS (ESI): *m*/*z* = 291.1 [M+Na]$^+$, 559.3 [2M+Na]$^+$.

$C_{17}H_{20}N_2O$ calcd.: 291.1468

found: 291.1468, [M+Na]$^+$ (ESI-HRMS).

(2*S*,5*S*)-(–)-5-Benzyl-3-methyl-2-(3,5-di-*tert*-butyl-phen-1-yl)-4-imidazolidinone (VII)

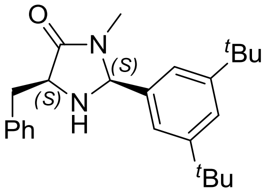

(*S*)-Phenylalaninmethylamide (178 mg, 1.00 mmol, 1.0 eq.), 3,5-di-*tert*-butylbenzyladehyde (240 mg,1.10 mmol, 1.1 eq.), 4 Å molecular sieves (80 mg) and $Yb(OTf)_3$ (62.1 mg, 100 µmol, 10 mol%) in THF (4.0 mL) were reacted according to GP1 for 72 h. Silica gel column chromatography (*n*-pentane:EtOAc = 4:1 → 1:1) gave the desired (*S*,*S*)-diastereomere **VII** (128 mg, 338 µmol, 34%) as white solid.

^{1}H-NMR (600 MHz, $CDCl_3$): δ = 1.24 (s, 18 H), 2.60 (s, 3 H), 3.18 (dd, *J* = 14.2, 5.3 Hz, 1 H), 3.27 (dd, *J* = 14.2, 5.3 Hz, 1 H), 3.85 (dd, *J* = 5.3, 5.3 Hz, 1 H), 5.14 (d, *J* = 1.7 Hz, 1 H), 6.85 (d, *J* = 1.7 Hz, 2 H), 7.21–7.31 (m, 5 H).

^{13}C-NMR (150 MHz, $CDCl_3$): δ = 27.6, 31.3, 34.8, 36.3, 60.6, 78.3, 121.1, 123.5, 127.1, 128.7, 129.7, 136.5, 137.0, 151.7, 175.1.

$\boldsymbol{\alpha_D^{24.0}}$ ($CHCl_3$, c = 1.0) = –90.0°.

IR (ATR) $\tilde{\nu}$ (cm^{-1}) = 2953, 1694, 1455, 1398, 1085.

MS (ESI): *m*/*z* = 401.3 $[M+Na]^+$, 779.5 $[2M+Na]^+$.

$C_{25}H_{34}N_2O$ calcd.: 401.2563

found: 401.2565, $[M+Na]^+$ (ESI-HRMS).

Preparation of the sulfenyl chlorides: The corresponding thiol (1.0 eq.) was added to a solution of NCS (1.25 eq.) in CH_2Cl_2 (0.2 mmol/mL) and stirred at ambient temperature for 30 min. The solvent was removed *in vacuo*, the residue taken up in *n*-pentane and filtrated. Removal of the solvent *in vacuo* gave the crude sulfenyl chloride, which was used without further purification.

General Procedure 2 (GP2): A *Schlenk* flask is charged with a magnetic stirrer and the organocatalyst (20 mol%), the atmosphere is changed to argon and the catalyst is dissolved in EtOAc or DME (0.1 mmol/mL with respect to the corresponding aldehyde). The reaction vessel is cooled to the given temperature. Subsequently cyclopropyl carbaldehyde (1.0 eq.) and chlorinated sulfenyl or selenyl reagent (1.2 eq.) are added and the resulting solution is stirred for the given time. After full consumption of the aldehyde EtOH (0.1 mmol/mL) and $NaBH_4$ (1.5 to 5.0 eq.) are added at the same temperature and the suspension is stirred for an additional time. After complete reduction the reaction is terminated *via* addition of a saturated aqueous solution of Rochelle's salt and allowed to warm to ambient temperature. The phases are separated, the aqueous layer is extracted with EtOAc, and the combined organic phases are washed with satured aqueous NaCl-solution, dried over Na_2SO_4 and the solvent is evaporated *in vacuo*. The diastereomeric ratios are determined *via* ^{1}H-NMR after column. Unless otherwise stated the major diastereomere is isolated in pure form.

The racemic products are prepared the same way by using a racemic mixture of the organocatalyst instead. **Note**: The cyclopropyl carbaldehydes may undergo autooxidation during storage, which can shutdown the catalyst activity and may lead to worse enantiomeric ratios.

Mosher Ester Formation: (*R*)-(–)-Mosher ester acid chloride (3.0 mg, 2.2 µl, 12 µmol, 1.2 eq.) and 4-DMAP (2.4 mg, 20 µmol, 2.0 eq.) are added to a solution of the 1,3-chalcohalogenated alcohol (10 µmol, 1.0 eq.) in CH_2Cl_2 (1.0 mL) and the resulting solution is stirred for 15 min at ambient temperature. The reaction mixture is terminated by the addition of aq. HCl (1.0 M, 1.0 mL). CH_2Cl_2 (2.0 mL) is added, phases are separated, the organic phase is washed with sat. aq. $NaHCO_3$ solution (2.5 mL), dried over Na_2SO_4 and the solvent is evaporated *in vacuo*. The enantiomeric ratios are determined of the corresponding crude Mosher esters *via* ^{19}F-NMR.

(*R*)-2-((1*R*,2*S*)-2-Chlorocyclohexyl)-2-(*p*-tolylthio)ethanol (3a)

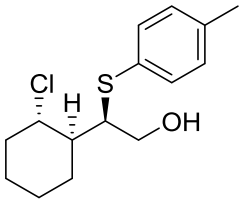

Neat *p*-tolylsulfenyl chloride (**2a**) (47.6 mg, 300 µmol, 1.2 eq.), *exo*-*meso*-bicyclo[4.1.0]heptane-7-carbaldehyde (**1a**) (31.0 mg, 250 µmol, 1.0 eq.) and **VI • DCA** (22.3 mg, 50.0 µmol, 20 mol%) in EtOAc (2.5 mL) were reacted according to GP2 for 90 min at –4 °C. Subsequent reduction with EtOH (2.5 mL) and $NaBH_4$ (47.3 mg, 5.0 eq.) showed full conversion after 15 min at –4 °C. Silica gel column chromatography (*n*-pentane:EtOAc = 10:1) gave the desired product **3a** (48.4 mg, 170 µmol, 68%, d.r. = 4.6:1) as white solid.

^{1}H-NMR (600 MHz, $CDCl_3$): δ = 1.22–1.38 (m, 3 H), 1.69–1.82 (m, 4 H), 1.90 (ddd, J = 9.3, 3.3. 3.3 Hz, 1 H), 2.05 (dddd, J = 11.5, 10.5, 3.8, 2.4 Hz, 1H), 2.33 (s, 3 H), 2.33–2.36 (m, 1 H), 3.72–3.76 (m, 2 H), 3.83 (ddd, J = 8.3, 6.2, 2.4 Hz, 1 H), 4.31 (ddd, J = 11.5, 10.5, 4.3 Hz, 1 H), 7.09–7.12 (m, 2 H), 7.42–7.45 (m, 2 H).

^{13}C-NMR (150 MHz, $CDCl_3$): δ = 21.1, 25.2, 26.3, 26.9, 37.6, 47.1, 55.3, 63.0, 64.0, 129.8, 131.4, 132.8, 137.5.

IR (ATR) $\tilde{\nu}$ (cm^{-1}) = 3320, 2938, 1491, 1217, 1005.

$\boldsymbol{\alpha_D^{24.0}}$ ($CHCl_3$, c = 1.0) = +30.0°.

MS (ESI): *m*/*z* = 307.1 $[M+Na]^+$, 591.2 $[2M+Na]^+$.

$C_{15}H_{21}ClOS$ calcd.: 307.0894

found: 307.0900, $[M+Na]^+$ (ESI-HRMS).

One recrystallization of the major diastereomere **3a** (940 mg, *e.r.* = 85:15) from *n*-hexane/Et_2O improved the *e.r.* to 97.5:2.5. The resulting crystals crystallize as racemic mixture, the further enantioenriched product (658 mg, 70%) stays in the mother liquor.

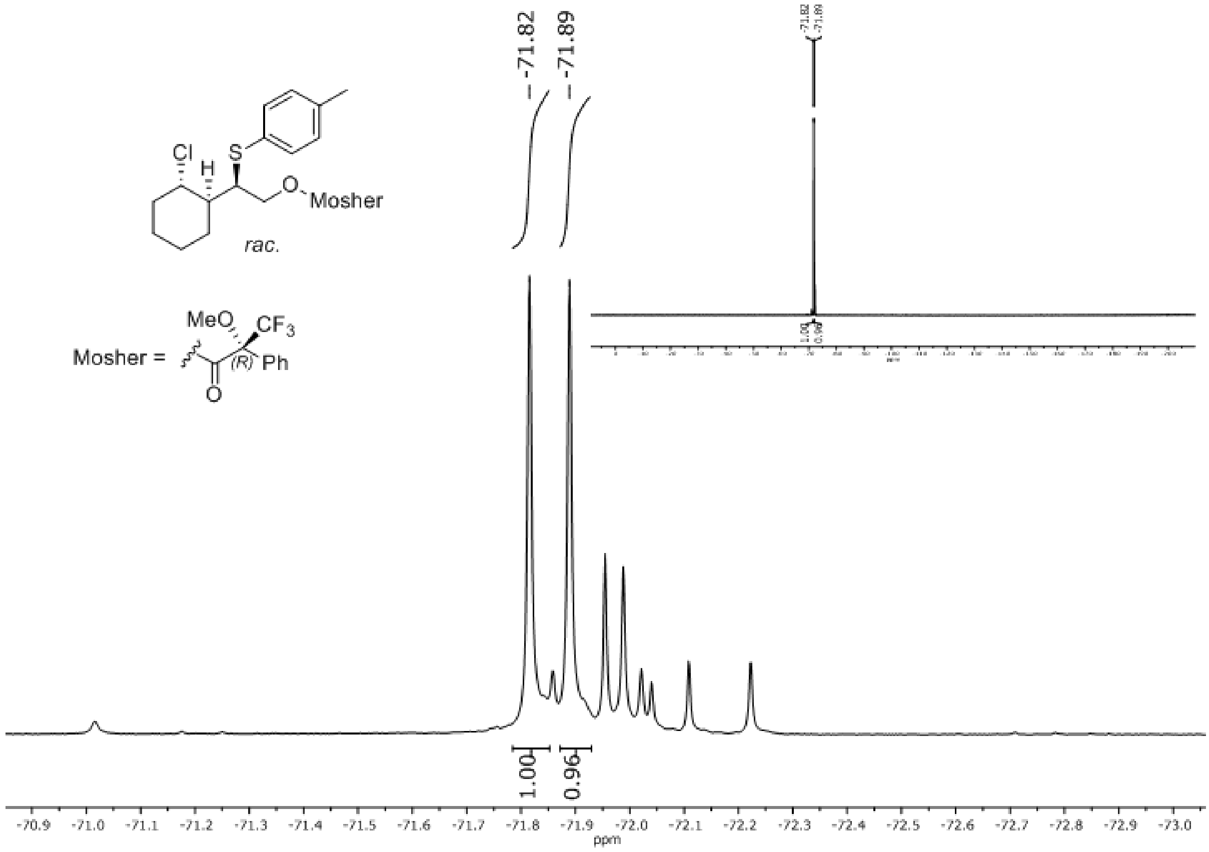

(^{19}F-NMR, 188 MHz, $CDCl_3$; top right: full spectra)

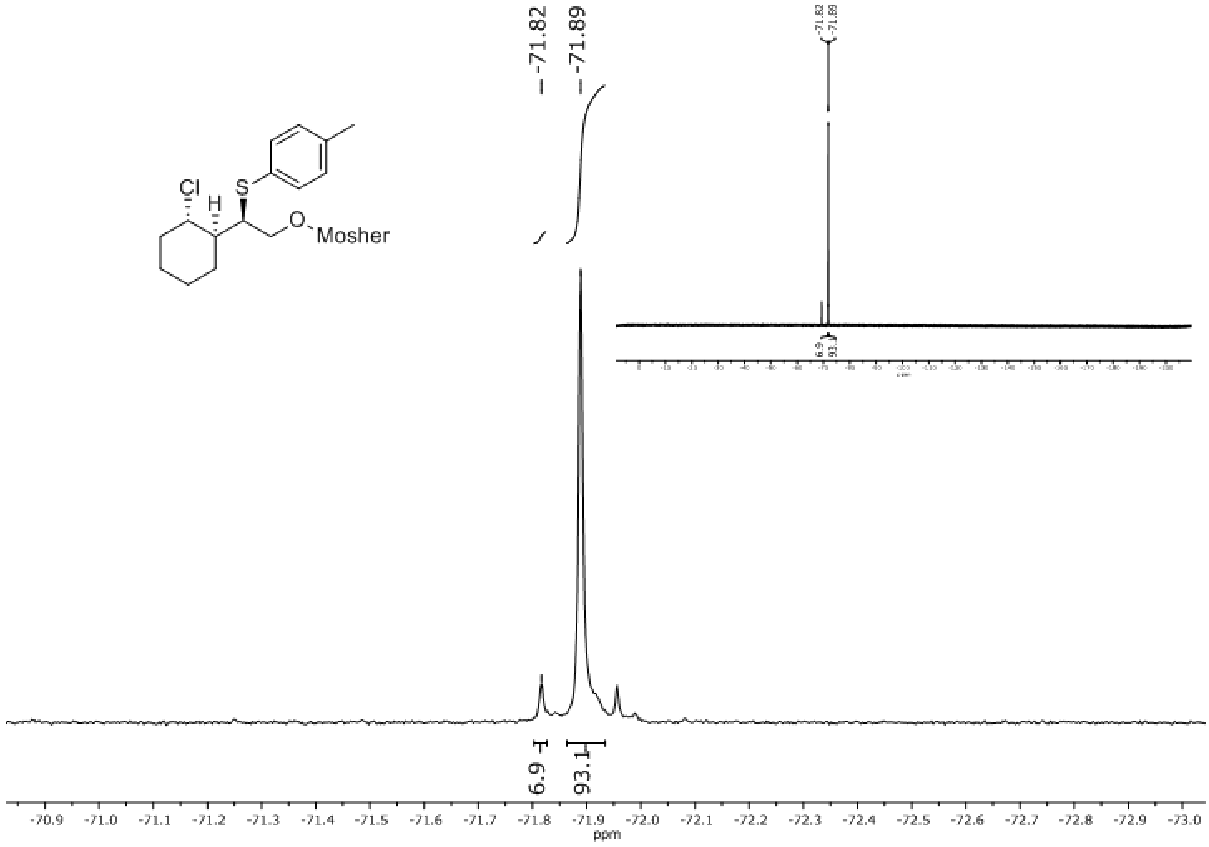

(^{19}F-NMR, 188 MHz, $CDCl_3$; top right: full spectra)

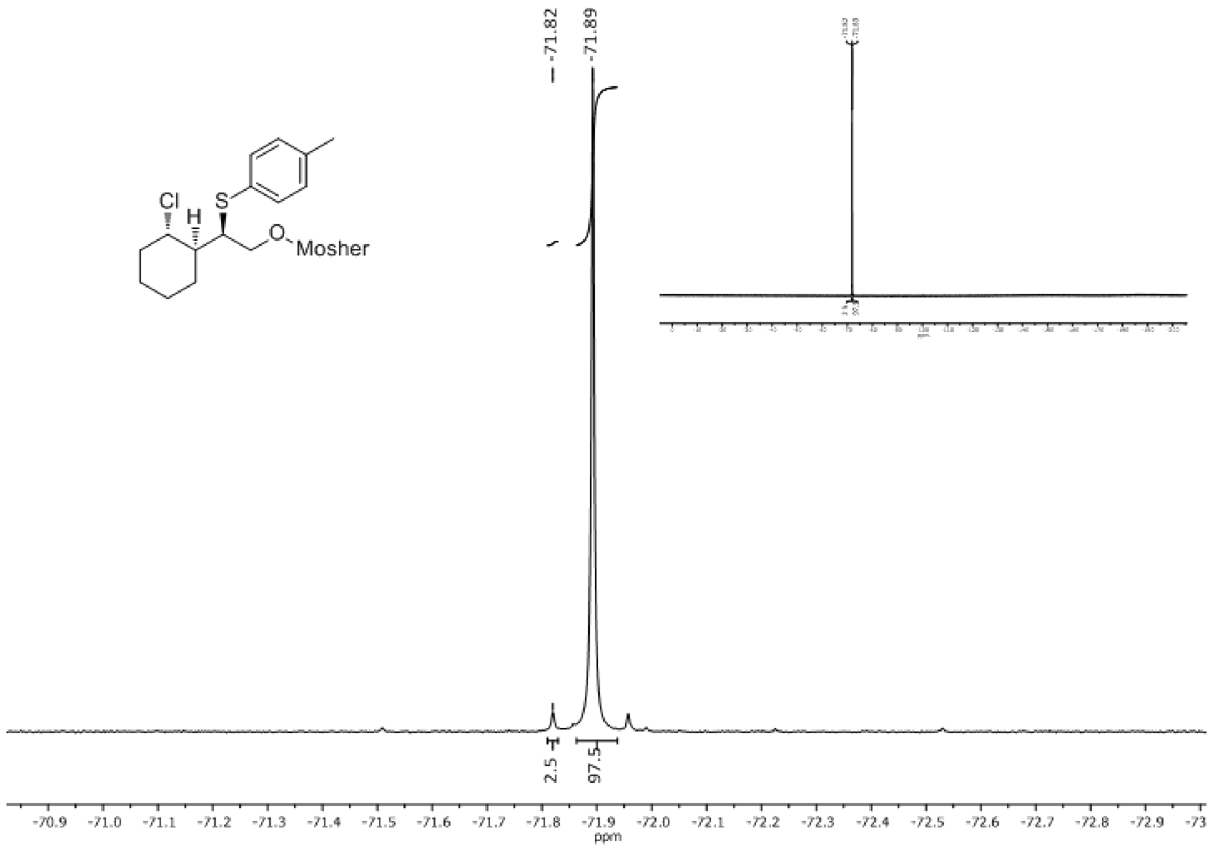

(^{19}F-NMR, 188 MHz, $CDCl_3$; top right: full spectra; after recrystallization)

(*R*)-2-((1*R*,2*S*)-2-Chlorocyclohexyl)-2-(*p*-anisolethio)ethanol (3b)

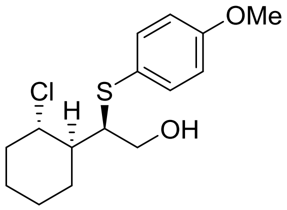

Neat *p*-anisolesulfenyl chloride (**2b**) (21.0 mg, 120 µmol, 1.2 eq.), *exo*-*meso*-bicyclo[4.1.0]heptane-7-carbaldehyde (**1a**) (12.4 mg, 100 µmol, 1.0 eq.) and **VI • DCA** (8.9 mg, 20 µmol, 20 mol%) in EtOAc (1.0 mL) were reacted according to GP2 for 90 min at –4 °C. Subsequent reduction with EtOH (1.0 mL) and $NaBH_4$ (18.9 mg, 5.0 eq.) showed full conversion after 15 min at –4 °C. Silica gel column chromatography (*n*-pentane:EtOAc = 5:1) gave the desired product **3b** (18.8 mg, 62.5 µmol, 63%, d.r. = 5.7:1) as colorless semi solid.

^{1}H-NMR (600 MHz, $CDCl_3$): δ = 1.20–1.38 (m, 3 H), 1.69–1.93 (m, 5 H), 2.04 (dddd, *J* = 11.6, 10.5, 4.1, 1.7 Hz, 1 H), 2.30–2.37 (m, 1 H), 3.70–3.76 (m, 3 H), 3.80 (s, 3 H), 4.34 (ddd, *J* = 11.6, 10.5, 4.1 Hz, 1 H), 6.84 (d, *J* = 8.9 Hz, 2 H), 7.49 (d, *J* = 8.9 Hz, 2 H).
^{13}C-NMR (150 MHz, $CDCl_3$): δ = 25.2, 26.3, 26.9, 37.5, 47.0, 55.3, 56.0, 63.1, 63.8, 114.6, 125.4, 135.2, 159.5.
IR (ATR) $\tilde{\nu}$ (cm^{-1}) = 3389, 2936, 1492, 1243, 1031.
$\boldsymbol{\alpha_D^{24.0}}$ ($CHCl_3$, c = 1.0) = +25.0°.
MS (ESI): *m*/*z* = 323.1 $[M+Na]^+$, 623.2 $[2M+Na]^+$.
$C_{15}H_{21}ClO_2S$ calcd.: 323.0843
found: 323.0845, $[M+Na]^+$ (ESI-HRMS)

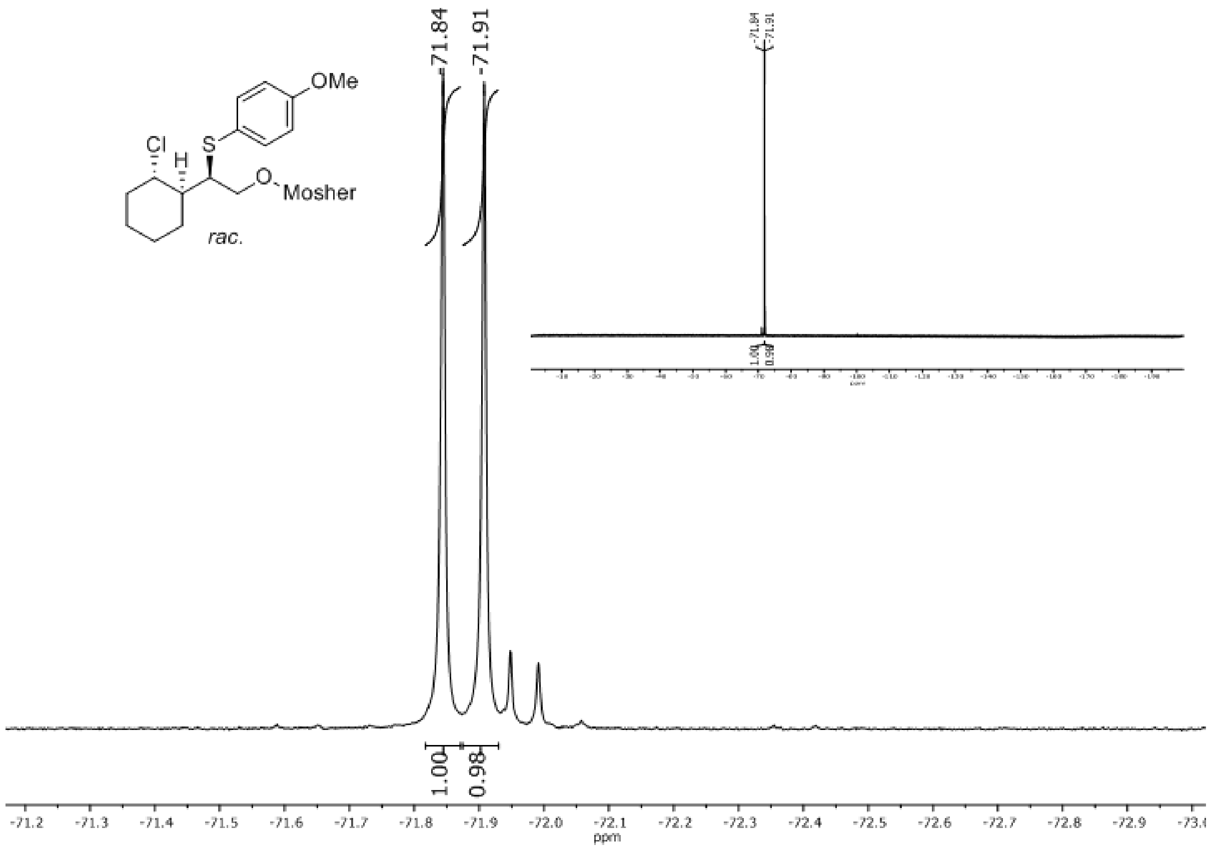

(^{19}F-NMR, 188 MHz, $CDCl_3$; top right: full spectra)

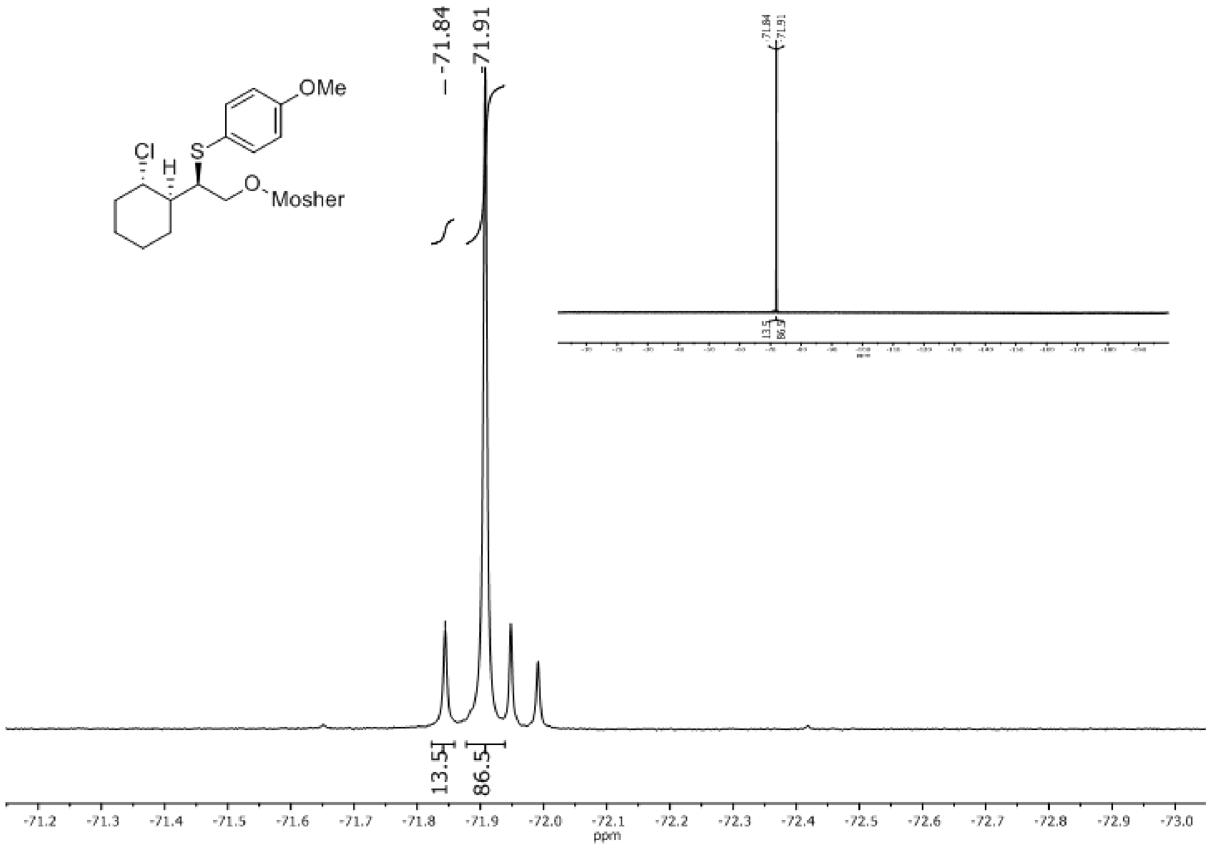

(^{19}F-NMR, 188 MHz, $CDCl_3$; top right: full spectra)

(*R*)-2-((1*R*,2*S*)-2-Chlorocyclohexyl)-2-(*p*-bromophenylthio)ethanol (3c)

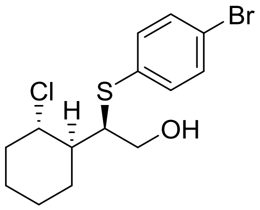

Neat *p*-bromophenylsulfenyl chloride (**2c**) (26.8 mg, 120 µmol, 1.2 eq.), *exo-meso*-bicyclo[4.1.0]heptane-7-carbaldehyde (**1a**) (12.4 mg, 100 µmol, 1.0 eq.) and **VI • DCA** (8.9 mg, 20 µmol, 20 mol%) in EtOAc (1.0 mL) were reacted according to GP2 for 90 min at –4 °C. Subsequent reduction with EtOH (1.0 mL) and $NaBH_4$ (18.9 mg, 5.0 eq.) showed full conversion after 15 min at –4 °C. Silica gel column chromatography (*n*-pentane:EtOAc = 10:1) gave the desired product **3c** (29.5 mg, 64.3 µmol, 84%, d.r. = 3.5:1) as white solid. Crystallization from DCM and *n*-hexane gave the pure major diastereomere.

^{1}H-NMR (600 MHz, $CDCl_3$): δ = 1.15–1.44 (m, 4 H), 1.67–1.85 (m, 3 H), 1.84–1.92 (m, 1 H), 2.07 (dddd, *J* = 11.6, 10.6, 4.0, 2.4 Hz, 1 H), 2.30–2.38 (m, 1 H), 3.69–3.80 (m, 2 H), 3.87 (ddd, *J* = 7.2, 7.2, 2.4 Hz, 1 H), 4.25 (ddd, *J* = 11.6, 10.6, 4.0 Hz, 1 H), 7.23–7.28 (m, 2 H), 7.38–7.46 (m, 2 H).
^{13}C-NMR (150 MHz, $CDCl_3$): δ = 25.2, 26.3, 26.9, 37.5, 46.9, 55.0, 62.9, 63.9, 121.3, 132.1, 133.7, 134.6.
IR (ATR) $\tilde{\nu}$ (cm^{-1}) = 3351, 2936, 1472, 1089, 1030.
$\boldsymbol{\alpha_D^{24.0}}$ ($CHCl_3$, c = 1.0) = +32.0°.
MS (ESI): *m*/*z* = 371.0 $[M+Na]^+$.

**$C_{14}H_{18}BrClOS$**	calcd.: 370.9843
	found: 370.9845, $[M+Na]^+$ (ESI-HRMS)

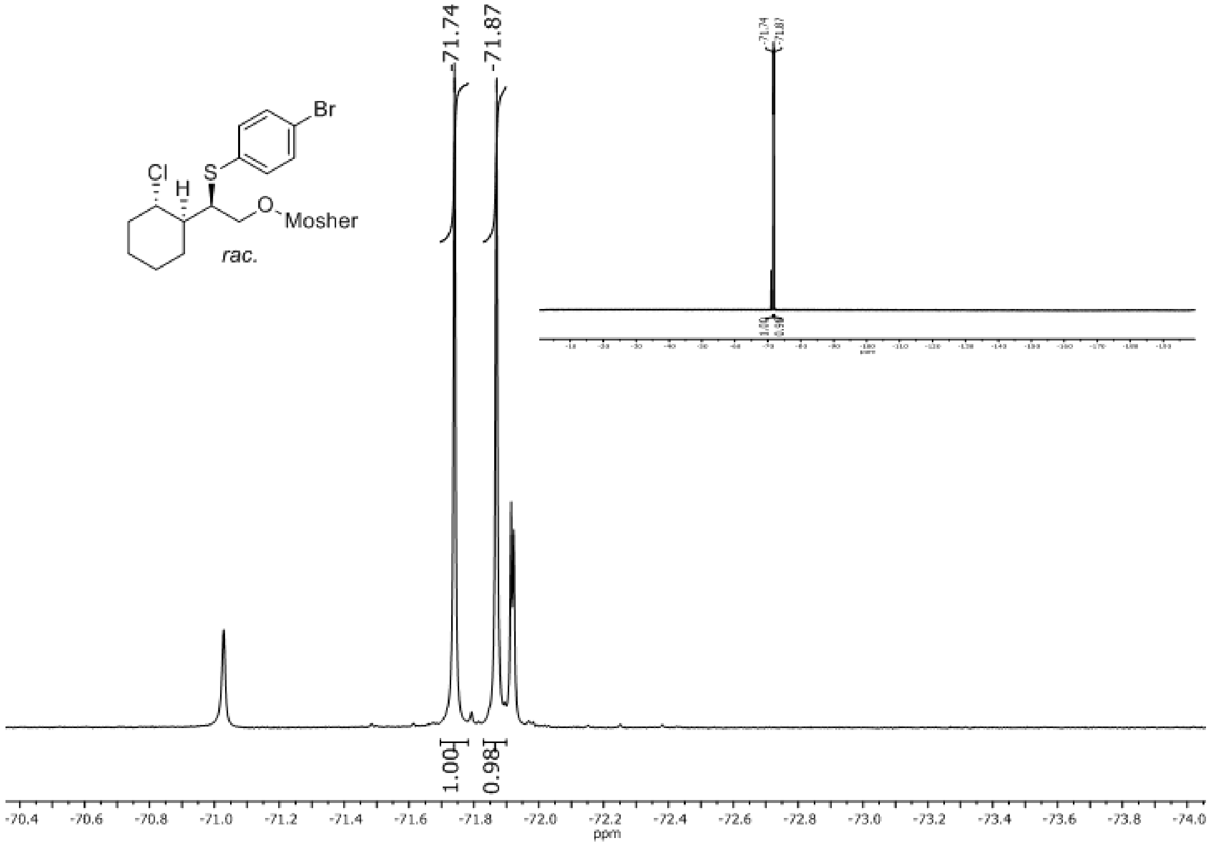

(^{19}F-NMR, 188 MHz, CDCl$_3$; top right: full spectra)

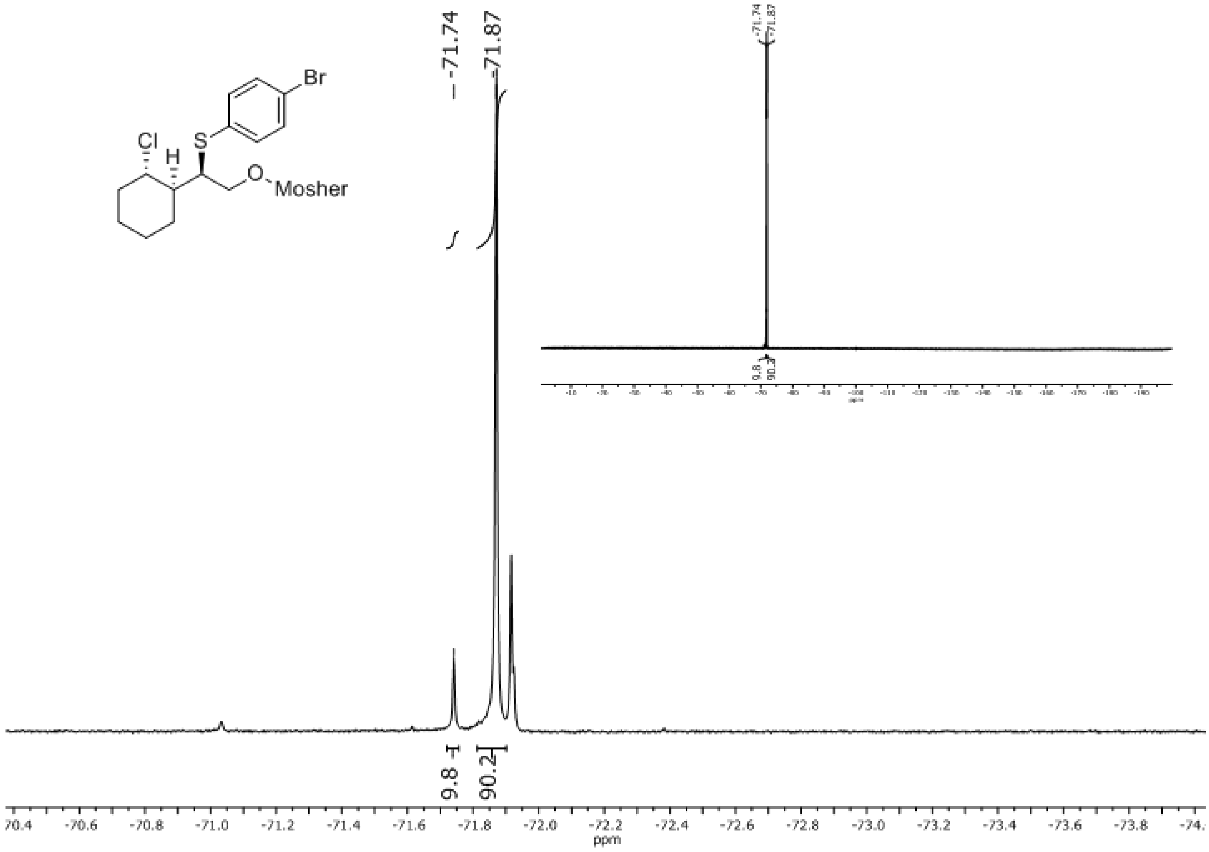

(^{19}F-NMR, 188 MHz, CDCl$_3$; top right: full spectra)

(*R*)-2-((1*R*,2*S*)-2-Chlorocyclohexyl)-2-(*p*-nitrophenylthio)ethanol (3d)

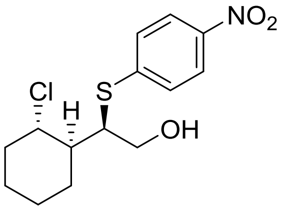

Neat *p*-nitrophenylsulfenyl chloride (**2d**) (45.5 mg, 240 µmol, 1.2 eq.), *exo*-*meso*-bicyclo[4.1.0]heptane-7-carbaldehyde (**1a**) (24.8 mg, 200 µmol, 1.0 eq.) and **VI • DCA** (17.8 mg, 40 µmol, 20 mol%) in EtOAc (2.0 mL) were reacted according to GP2 for 120 min at –4 °C. Subsequent reduction with EtOH (2.0 mL) and $NaBH_4$ (37.8 mg, 5.0 eq.) showed full conversion after 15 min at –4 °C. Silica gel column chromatography (*n*-pentane:EtOAc = 10:1) gave the desired product **3d** (39.3 mg, 124 µmol, 62%, d.r. = 3.1:1) as slight yellow oil (mixture of diastereomeres, no sepearation on column possible).

^{1}H-NMR (300 MHz, $CDCl_3$): δ = 1.23–1.38 (m, 3 H), 1.67–1.93 (m, 5 H), 2.07–2.19 (m, 1 H), 2.25–2.38 (m, 1 H), 3.73–3.88 (m, 2 H), 4.00–4.25 (m, 2 H), 7.49–7.55 (m) and 7.57–7.64 (m, together 2 H), 8.10–8.18 (m, 2 H).
^{13}C-NMR (150 MHz, $CDCl_3$)
major diastereomere: δ = 25.2, 26.2, 27.0, 37.4, 46.7, 53.0, 62.8, 64.1, 124.0, 129.2, 145.9, 145.9.
minor diastereomere: δ = 25.5, 26.4, 27.8, 38.0, 49.6, 51.7, 61.4, 63.3, 124.1, 127.8, 145.9, 146.0.
MS (ESI): *m*/*z* = 338.1 [M+Na]$^+$.
$C_{14}H_{18}ClNO_3S$ calcd.: 338.0588
found: 338.0589, [M+Na]$^+$ (ESI-HRMS)

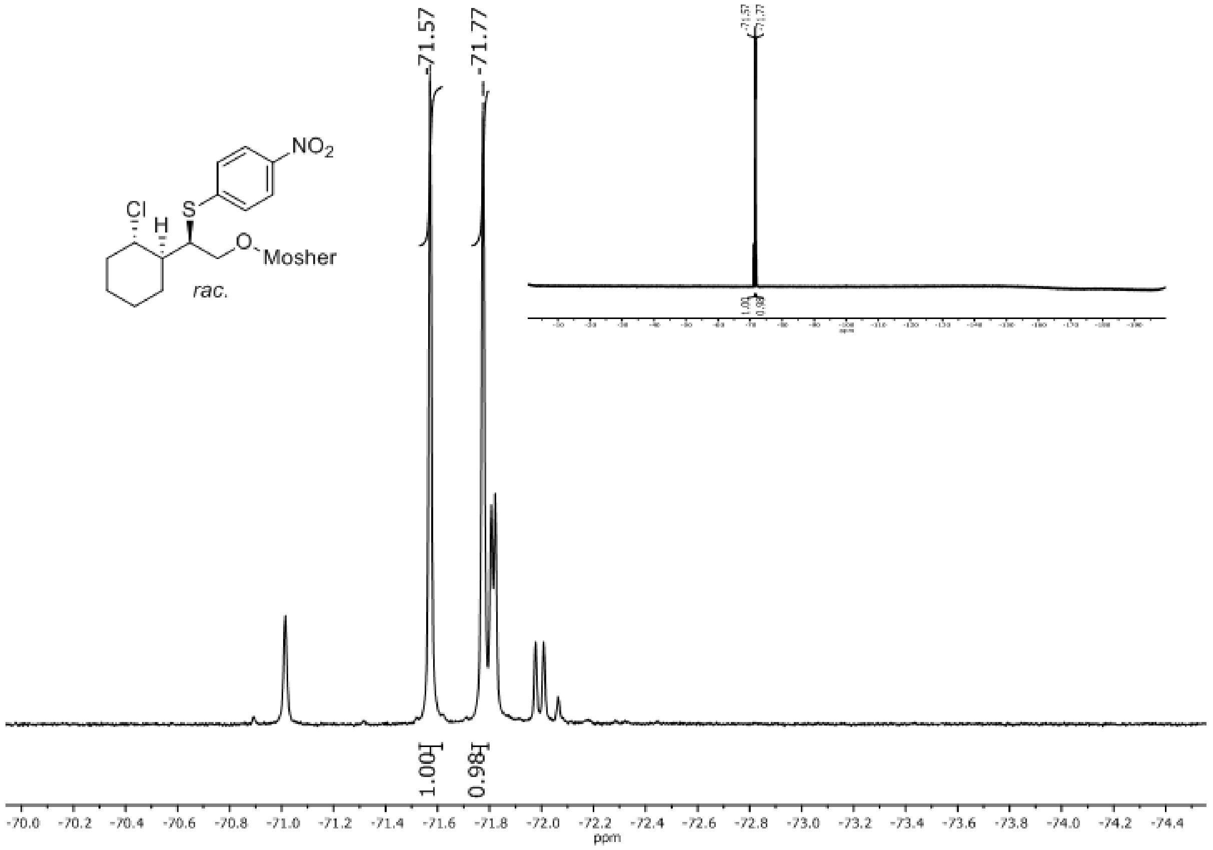

(^{19}F-NMR, 188 MHz, $CDCl_3$; top right: full spectra)

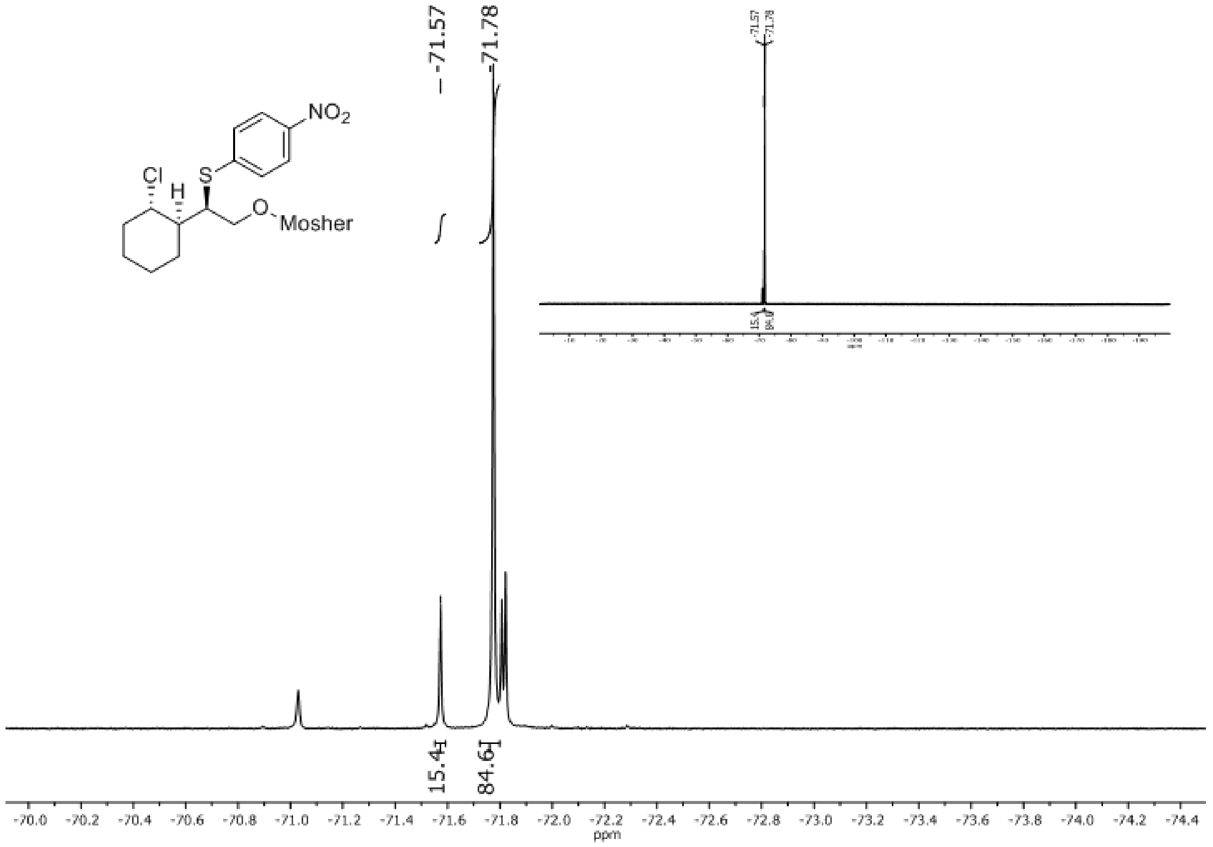

(^{19}F-NMR, 188 MHz, $CDCl_3$; top right: full spectra)

(*R*)-2-((1*R*,2*S*)-2-Chlorocyclohexyl)-2-(*p*-fluorophenylthio)ethanol (3e)

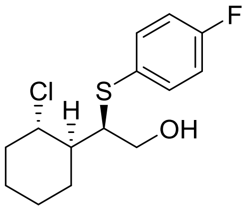

Neat *p*-fluorophenylsulfenyl chloride (**2e**) (19.4 mg, 120 µmol, 1.2 eq.), *exo*-*meso*-bicyclo[4.1.0]heptane-7-carbaldehyde (**1a**) (12.4 mg, 100 µmol, 1.0 eq.) and **VI • DCA** (8.9 mg, 20 µmol, 20 mol%) in EtOAc (1.0 mL) were reacted according to GP2 for 90 min at –4 °C. Subsequent reduction with EtOH (1.0 mL) and $NaBH_4$ (18.9 mg, 5.0 eq.) showed full conversion after 15 min at –4 °C. Silica gel column chromatography (*n*-pentane:EtOAc = 10:1) gave the desired product **3e** (17.5 mg, 60.6 µmol, 61%, d.r. = 2.5:1) as colorless oil (mixture of diastereomeres, no sepearation on column possible).

^{1}H-NMR (300 MHz, $CDCl_3$): δ = 1.19–1.40 (m, 3 H), 1.61–1.83 (m, 4 H), 1.87–1.92 (m, 1 H), 1.96–2.09 (m, 1 H), 2.22–2.37 (m, 1 H), 3.68 – 3.71 (m) and 3.73–3.77 (m, together 2 H), 3.76–3.84 (m, 1 H), 4.04 (ddd, *J* = 10.8, 10.8, 4.2 Hz) and 4.27–4.32 (m, together 1 H), 6.97–7.04 (m, 2 H), 7.44–7.48 (m) and 7.51–7.55 (m, together 2 H).

^{13}C-NMR (150 MHz, $CDCl_3$)

major diastereomere: δ = 25.2, 26.3, 26.9, 37.5, 47.0, 55.8, 63.0, 63.8, 116.2 (d, ^{2}J = 21.9 Hz), 130.3 (d, ^{4}J = 3.4 Hz), 134.9 (d, ^{3}J = 8.2 Hz), 162.4 (d, ^{1}J = 247.7 Hz).

minor diastereomere: δ = 25.4, 26.2, 27.7, 37.7, 49.3, 55.2, 61.4, 63.2, 116.3 (d, ^{2}J = 21.9 Hz), 129.8 (d, ^{4}J = 3.4 Hz), 134.0 (d, ^{3}J = 8.2 Hz), 162.3 (d, ^{1}J = 247.7 Hz).

^{19}F-NMR (188 MHz, $CDCl_3$)

major diastereomere: δ = –114.5.

minor diastereomere: δ = –114.6.

MS (ESI): *m*/*z* = 311.1 $[M+Na]^+$.

$C_{14}H_{18}ClFOS$ calcd.: 311.0643

found: 311.0648, $[M+Na]^+$ (ESI-HRMS)

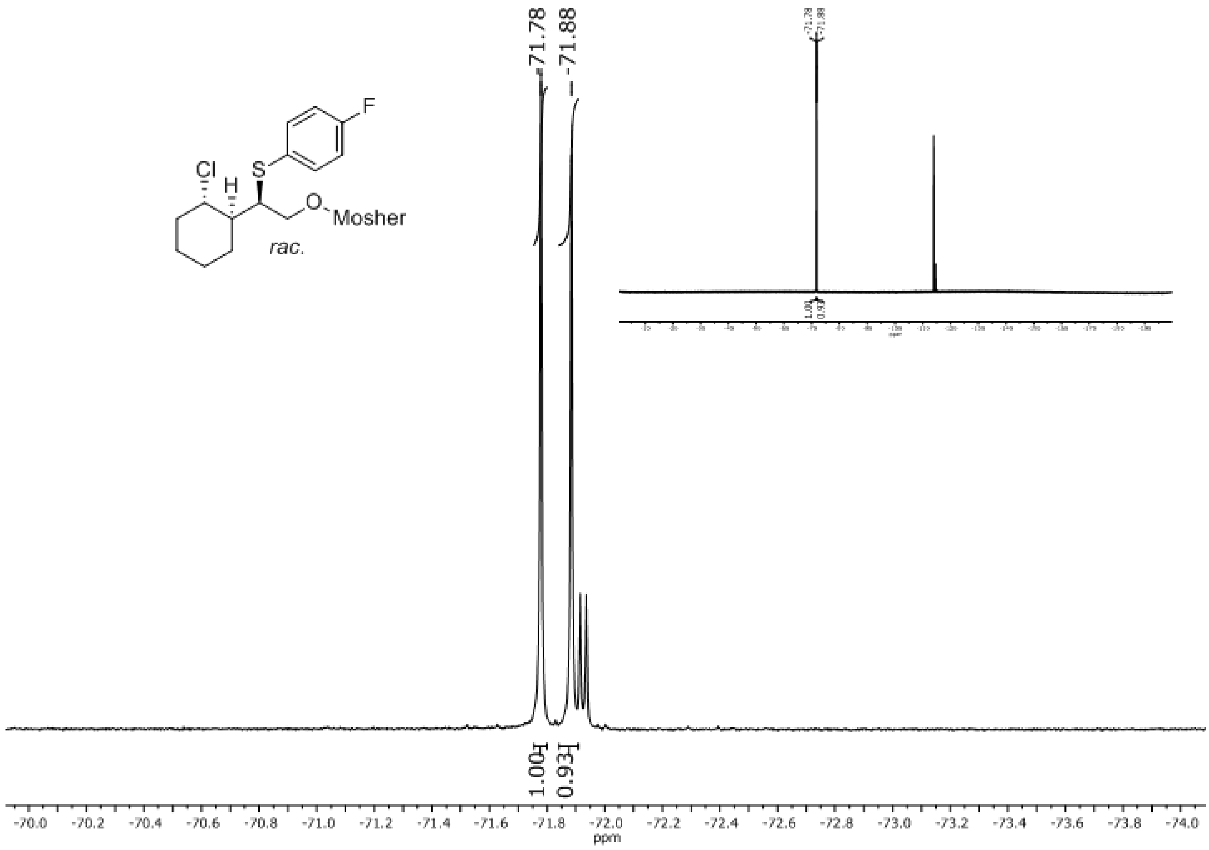

(^{19}F-NMR, 188 MHz, $CDCl_3$; top right: full spectra)

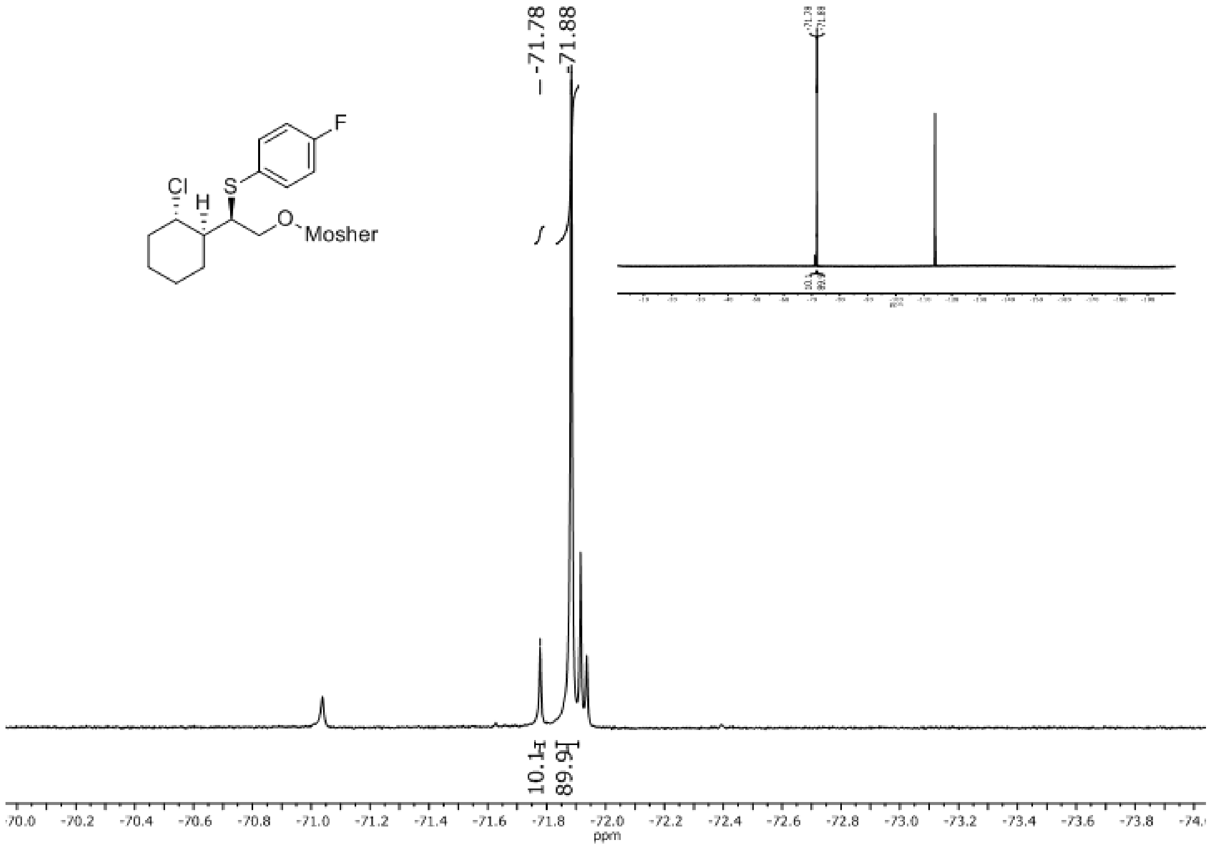

(^{19}F-NMR, 188 MHz, $CDCl_3$; top right: full spectra)

(*R*)-2-((1*R*,2*S*)-2-Chlorocyclohexyl)-2-(cyclohexylthio)ethanol (3f)

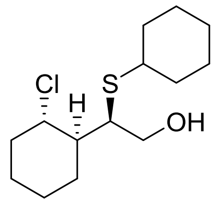

Neat cyclohexylsulfenyl chloride (**2f**) (18.1 mg, 120 µmol, 1.2 eq.), *exo*-*meso*-bicyclo[4.1.0]heptane-7-carbaldehyde (**1a**) (12.4 mg, 100 µmol, 1.0 eq.) and **VI • DCA** (8.9 mg, 20 µmol, 20 mol%) in EtOAc (1.0 mL) were reacted according to GP2 for 90 min at –4 °C. Subsequent reduction with EtOH (1.0 mL) and $NaBH_4$ (18.9 mg, 5.0 eq.) showed full conversion after 15 min at –4 °C. Silica gel column chromatography (*n*-pentane:EtOAc = 15:1) gave the desired product **3f** (11.6 mg, 41.9 µmol, 42%, d.r. = 15:1) as colorless oil.

^{1}H-NMR (600 MHz, $CDCl_3$): δ = 1.18–1.38 (m, 8 H), 1.67–1.81 (m, 7 H), 1.90 (dddd, *J* = 10.3, 10.3, 3.4, 2.2 Hz, 1 H), 1.98–2.11 (m, 3 H), 2.31 (dddd, *J* = 11.4, 4.6, 3.4, 1.6 Hz, 1 H), 2.75 (ddd, *J* = 10.4, 4.6, 3.9 Hz, 1 H), 3.48 (ddd, *J* = 7.3, 7.3, 2.2 Hz, 1 H), 3.58–3.70 (m, 2 H), 4.17 (ddd, *J* = 11.4, 10.4, 4.6 Hz, 1 H).

^{13}C-NMR (150 MHz, $CDCl_3$): δ = 25.1, 25.7, 26.1, 26.1, 26.2, 26.9, 34.2, 34.8, 37.6, 44.9, 47.5, 49.4, 63.2, 64.5.

IR (ATR) $\tilde{\nu}$ (cm^{-1}) = 3353, 2927, 2853, 1447, 1054.

$\boldsymbol{\alpha_D^{24.0}}$ ($CHCl_3$, c = 1.0) = +11.0°.

MS (ESI): *m*/*z* = 299.1 $[M+Na]^+$, 575.3 $[2M+Na]^+$.

$C_{14}H_{25}ClOS$ calcd.: 299.1207

found: 299.1210, $[M+Na]^+$ (ESI-HRMS)

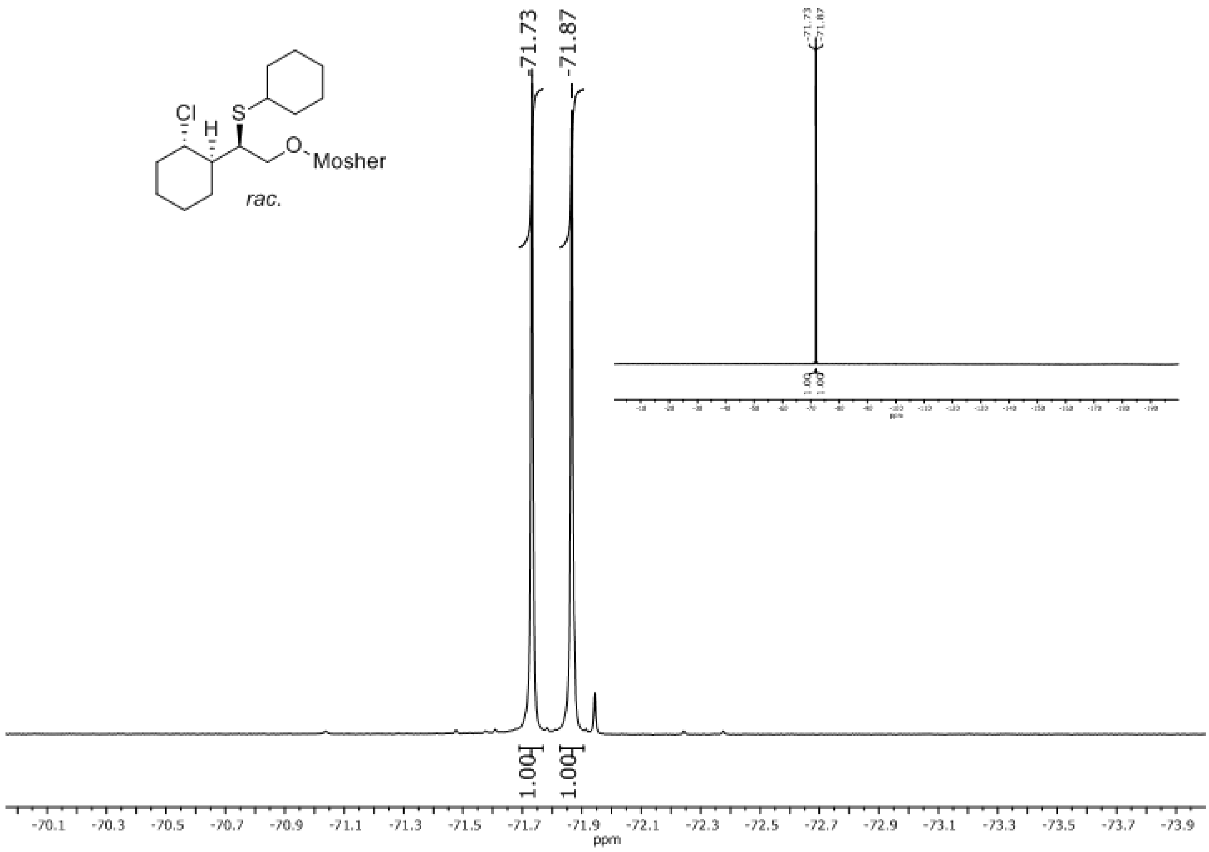

(^{19}F-NMR, 188 MHz, $CDCl_3$; top right: full spectra)

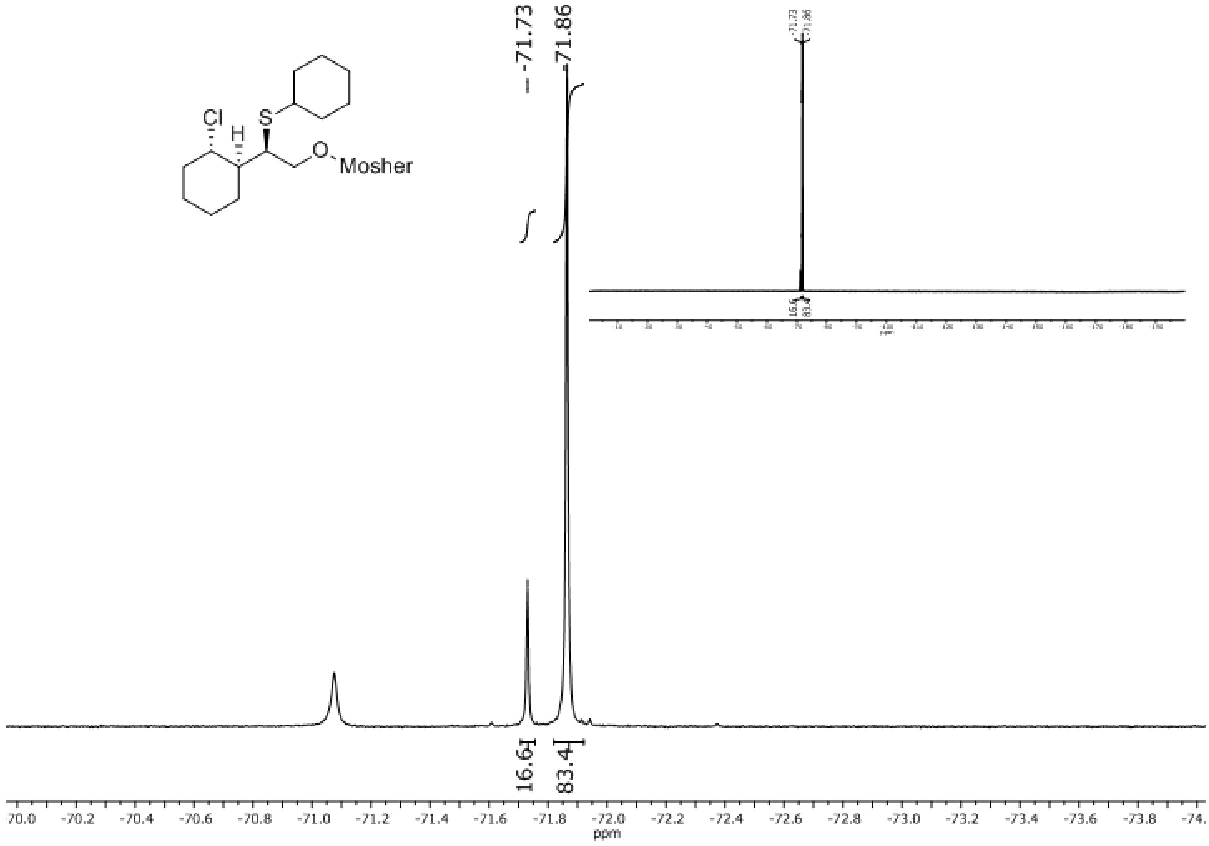

(^{19}F-NMR, 188 MHz, $CDCl_3$; top right: full spectra)

(*R*)-2-((1*R*,2*S*)-2-Chlorocyclohexyl)-2-(*tert*-butyldisulfanyl)ethanol (3g)

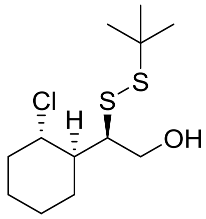

Neat *tert*-butylsulfenyl chloride (**2g**) (24.9 mg, 200 µmol, 2.0 eq.), *exo*-*meso*-bicyclo[4.1.0]heptane-7-carbaldehyde (**1a**) (12.4 mg, 100 µmol, 1.0 eq.) and **VI • DCA** (8.9 mg, 20 µmol, 20 mol%) in EtOAc (1.0 mL) were reacted according to GP2 for 90 min at –4 °C. Subsequent reduction with EtOH (1.0 mL) and $NaBH_4$ (18.9 mg, 5.0 eq.) showed full conversion after 15 min at –4 °C. Silica gel column chromatography (*n*-pentane:EtOAc = 20:1) gave the desired product **3g** (17.2 mg, 60.8 µmol, 61%, d.r. = 4.2:1) as white solid.

^{1}H-NMR (600 MHz, $CDCl_3$): δ = 1.22–1.35 (m, 4 H), 1.38 (s, 9 H), 1.67–1.81 (m, 3 H), 1.84–1.90 (m, 1 H), 2.06 (dddd, *J* = 12.0, 10.6, 3.7, 2.3 Hz, 1 H), 2.29–2.36 (m, 1 H), 3.60 (ddd, *J* = 7.9, 5.4, 2.3 Hz, 1 H), 3.76–3.83 (m, 1 H), 3.96 (ddd, *J* = 11.1, 5.3, 5.3 Hz, 1 H), 4.20 (ddd, *J* = 11.1, 4.4, 4.4 Hz, 1 H).

^{13}C-NMR (150 MHz, $CDCl_3$): δ = 25.3, 26.2, 26.6, 29.9, 37.8, 47.3, 47.9, 56.0, 62.7, 64.4.

IR (ATR) $\tilde{\nu}$ (cm^{-1}) = 3366, 2937, 2859, 1451, 1033.

$\boldsymbol{\alpha_D^{24.0}}$ ($CHCl_3$, c = 1.0) = +37.0°.

MS (ESI): *m*/*z* = 305.1 $[M+Na]^+$, 587.2 $[2M+Na]^+$.

$C_{12}H_{23}ClOS_2$ calcd.: 305.0771

found: 305.0774, $[M+Na]^+$ (ESI-HRMS)

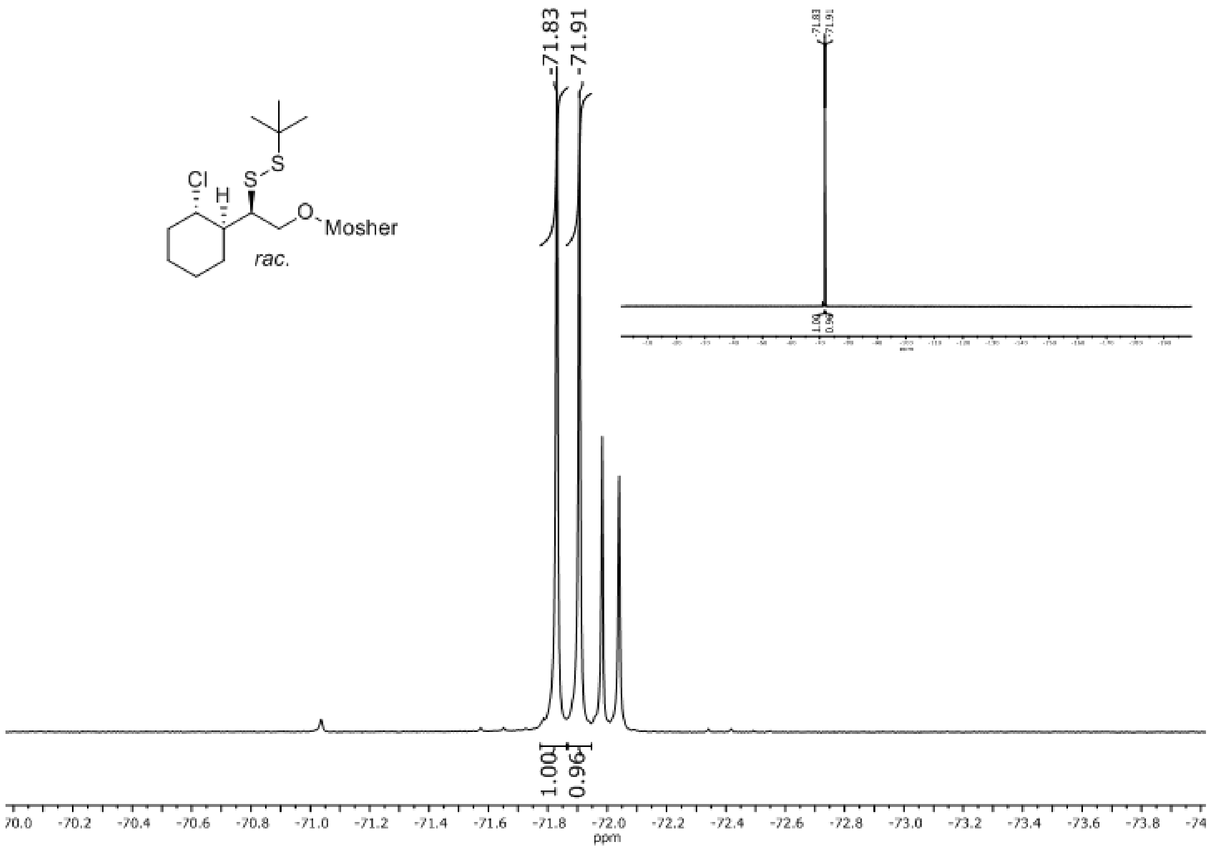

(^{19}F-NMR, 188 MHz, $CDCl_3$; top right: full spectra)

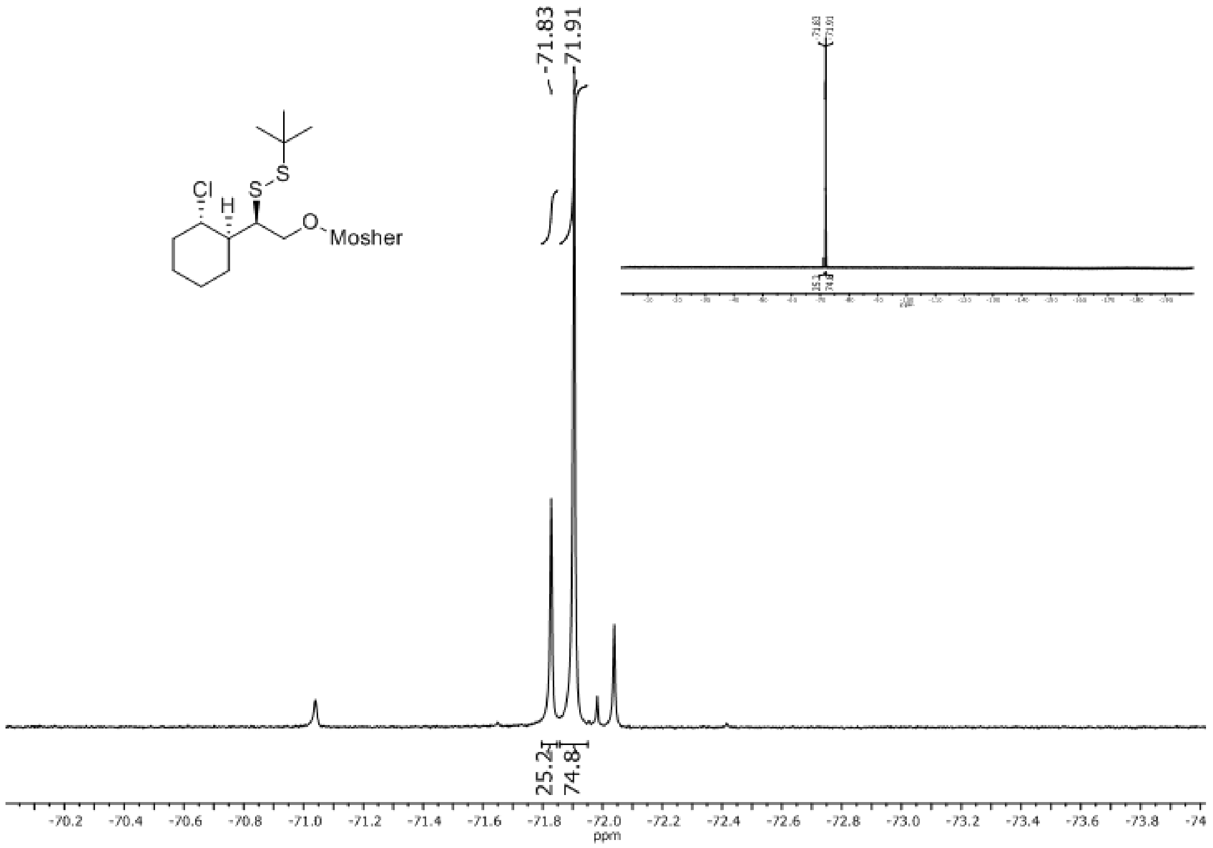

(^{19}F-NMR, 188 MHz, $CDCl_3$; top right: full spectra)

(*R*)-2-((1*R*,2*S*)-2-Chlorocyclohexyl)-2-(methoxycarbonylthio)ethanol (3h)

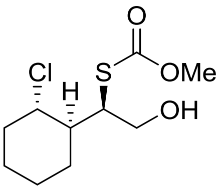

Neat methoxycarbonylsulfenyl chloride (**2h**) (15.2 mg, 10.9 µL,120 µmol, 1.2 eq.), *exo*-*meso*-bicyclo[4.1.0]heptane-7-carbaldehyde (**1a**) (12.4 mg, 100 µmol, 1.0 eq.) and **VI • DCA** (8.9 mg, 20 µmol, 20 mol%) in EtOAc (1.0 mL) were reacted according to GP2 for 90 min at –4 °C. Subsequent reduction with EtOH (1.0 mL) and $NaBH_4$ (5.7 mg, 1.5 eq.) showed full conversion after 60 min at–4 °C. Silica gel column chromatography (DCM) gave the desired product **3h** (14.2 mg, 56.2 µmol, 56%, d.r. = 4.2:1) as colorless oil.

^{1}H-NMR (600 MHz, $CDCl_3$): δ = 1.04 (ddd, *J* = 12.5, 12.5, 3.8 Hz, 1 H), 1.22–1.34 (m, 3 H), 1.67–1.80 (m, 3 H), 1.85–1.92 (m, 1 H), 2.11 (dddd, *J* = 12.5, 10.9, 3.8, 2.5 Hz, 1 H), 2.28–2.33 (m, 1 H), 3.77–3.83 (m, 2 H), 3.84 (s, 3 H), 3.98 (ddd, *J* = 10.9, 10.9, 4.3 Hz, 1 H), 4.23 (ddd, *J* = 7.3, 7.3, 2.5 Hz, 1 H).

^{13}C-NMR (150 MHz, $CDCl_3$): δ = 25.2, 26.2, 28.0, 37.5, 45.8, 50.0, 54.6, 62.2, 64.6, 170.9.

IR (ATR) $\tilde{\nu}$ (cm^{-1}) = 3408, 2937, 1714, 1448, 1147.

$\boldsymbol{\alpha_D^{24.0}}$ ($CHCl_3$, c = 1.0) = –14.0°.

MS (ESI): *m*/*z* = 275.0 $[M+Na]^+$, 527.1 $[2M+Na]^+$.

$C_{10}H_{17}ClO_3S$ calcd.: 275.0479

found: 275.0483, $[M+Na]^+$ (ESI-HRMS)

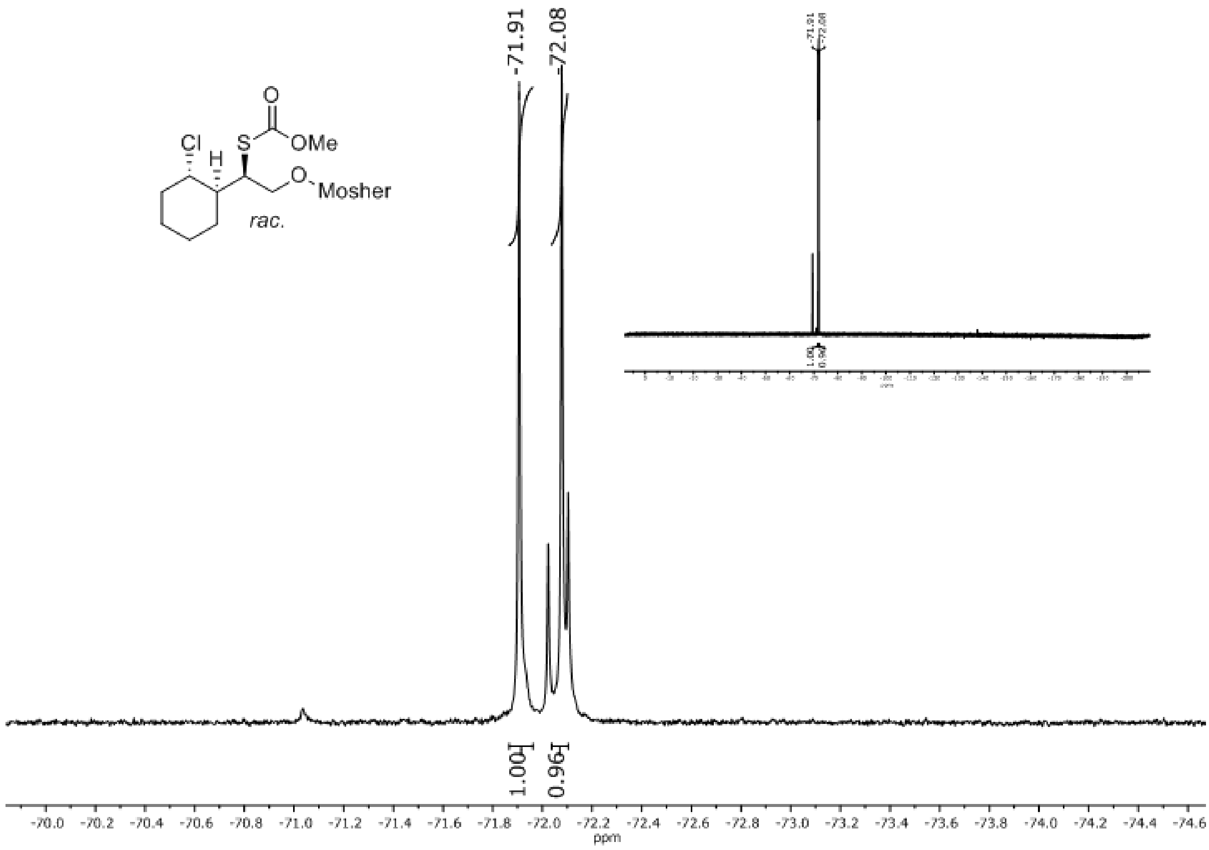

(^{19}F-NMR, 188 MHz, $CDCl_3$; top right: full spectra)

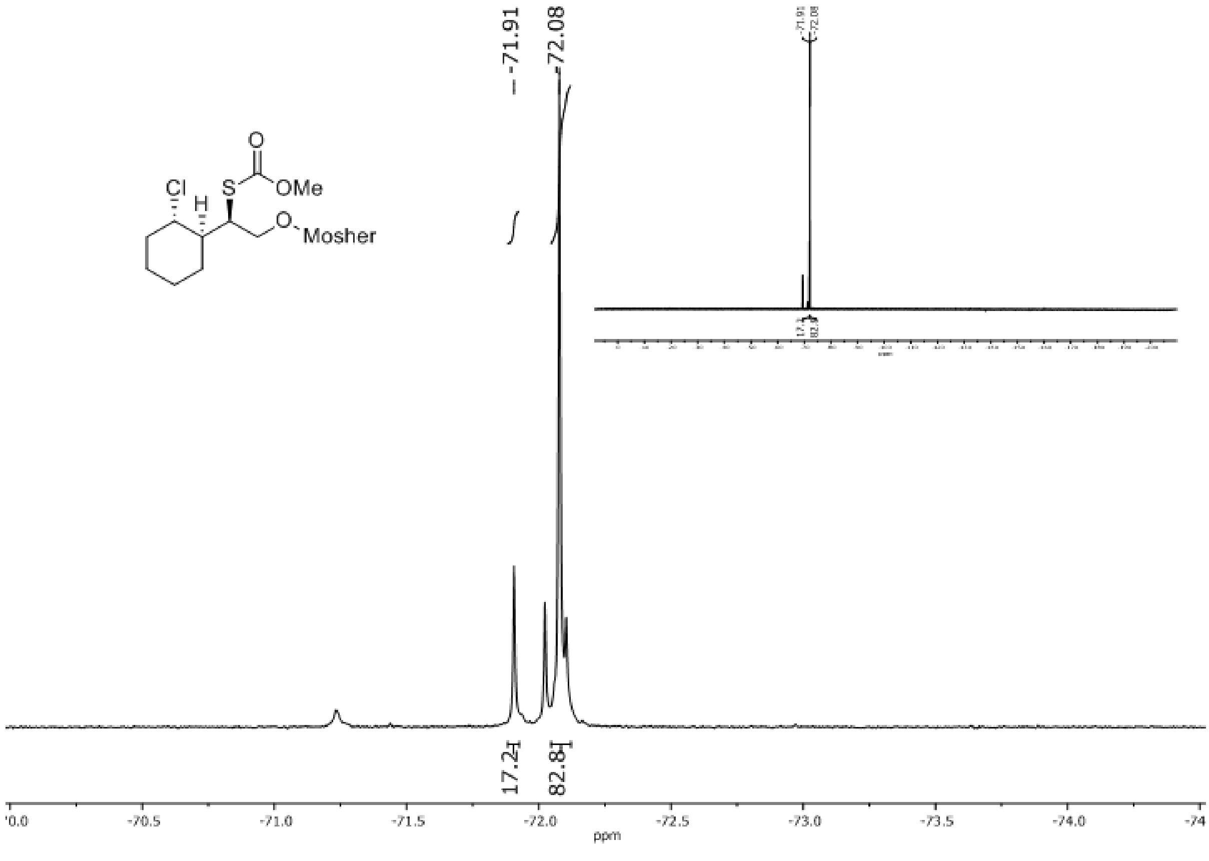

(^{19}F-NMR, 188 MHz, $CDCl_3$; top right: full spectra)

(*R*)-2-((1*R*,2*S*)-2-Chlorocyclohexyl)-2-(phenylseleno)ethanol (3i)

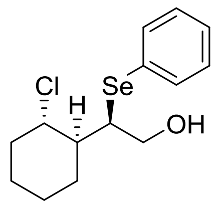

Neat phenylselenyl chloride (**2i**) (23.0 mg, 120 µmol, 1.2 eq.), *exo*-*meso*-bicyclo[4.1.0]heptane-7-carbaldehyde (**1a**) (12.4 mg, 100 µmol, 1.0 eq.) and **VI • DCA** (8.9 mg, 20 µmol, 20 mol%) in EtOAc (1.0 mL) were reacted according to GP2 for 90 min at –4 °C. Subsequent reduction with EtOH (1.0 mL) and $NaBH_4$ (18.9 mg, 5.0 eq.) showed full conversion after 15 min at –4 °C. Silica gel column chromatography (*n*-pentane:EtOAc = 10:1) gave the desired product **3i** (22.8 mg, 71.7 µmol, 72%, d.r. = 5.7:1) as colorless oil.

^{1}H-NMR (300 MHz, $CDCl_3$): δ = 1.07–1.41 (m, 4 H), 1.70–1.87 (m, 3 H), 1.93–2.07 (m, 2 H), 2.26–2.41 (m, 1 H), 3.77–3.89 (m, 2 H), 3.94 (ddd, *J* = 8.3, 6.3, 2.1 Hz, 1 H), 4.30 (ddd, *J* = 11.3, 10.1, 4.3 Hz, 1 H), 7.24–7.30 (m, 3 H), 7.63–7.69 (m, 2 H).

^{13}C-NMR (75 MHz, $CDCl_3$): δ = 25.2, 26.3, 28.3, 37.5, 47.1, 53.8, 63.7, 64.5, 127.7, 129.2, 129.2, 134.6.

IR (ATR) $\tilde{\nu}$ (cm^{-1}) = 3367, 2934, 1440, 1217, 1052.

$\boldsymbol{\alpha_D^{24.0}}$ ($CHCl_3$, c = 1.0) = +43.0°.

MS (ESI): *m*/*z* = 341.0 [M+Na]$^+$, 659.0 [2M+Na]$^+$.

$C_{14}H_{19}ClOSe$ calcd.: 341.0182

found: 341.0181, [M+Na]$^+$ (ESI-HRMS)

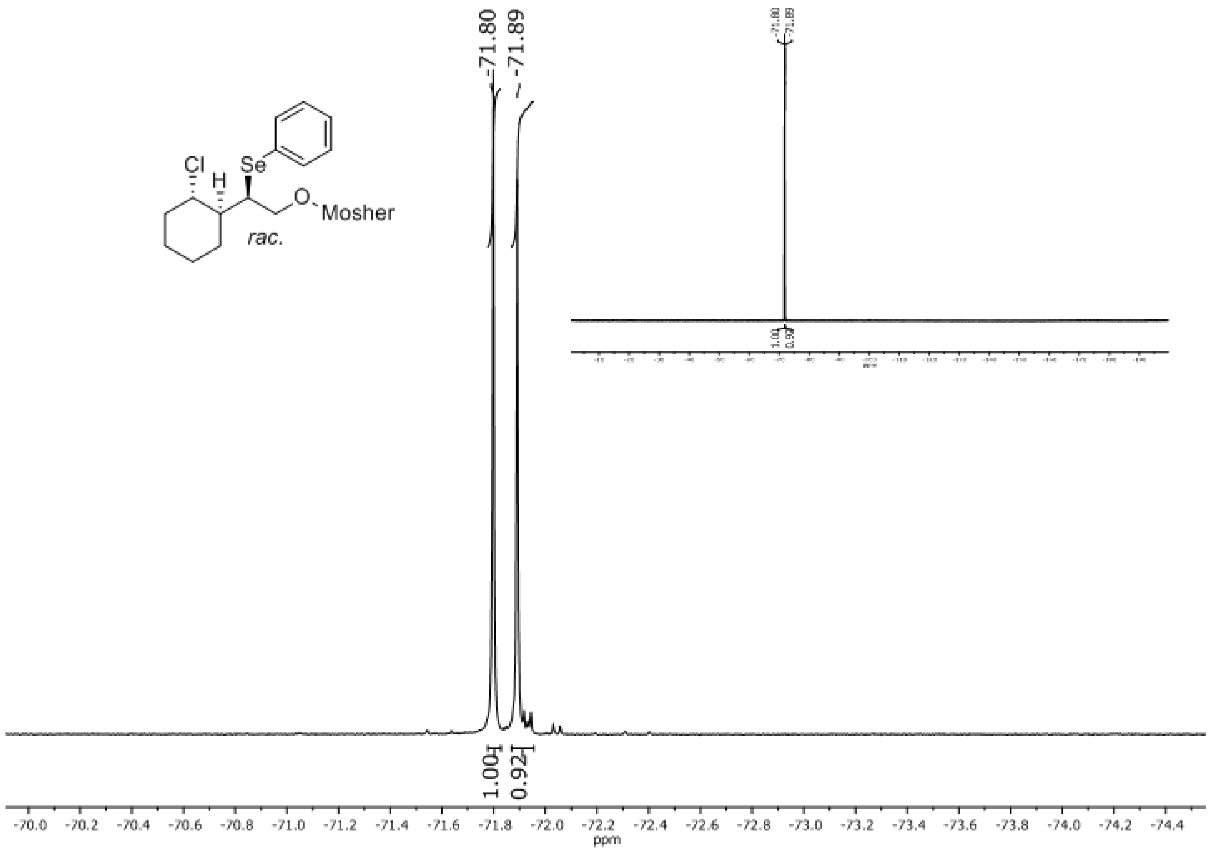

(^{19}F-NMR, 188 MHz, $CDCl_3$; top right: full spectra)

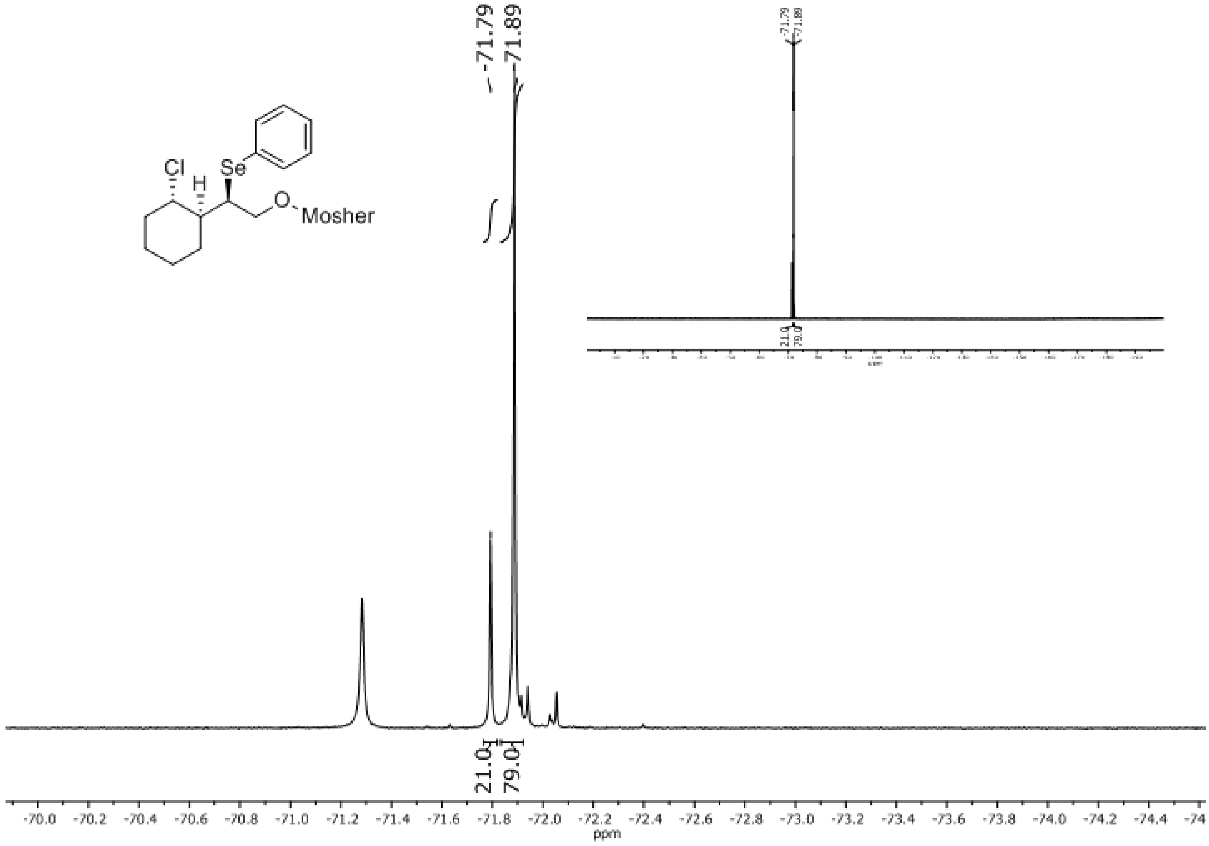

(^{19}F-NMR, 188 MHz, $CDCl_3$; top right: full spectra)

(2*R*,3*R*,4*S*)-4-Chloro-3-ethyl-2-(*p*-tolylthio)hexan-1-ol (4b)

(2*S*,3*R*,4*S*)-4-Chloro-3-ethyl-2-(*p*-tolylthio)hexan-1-ol (4b')

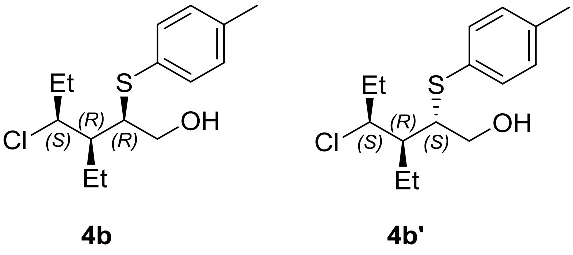

Neat *p*-toylsulfenyl chloride (**2a**) (38.1 mg, 240 µmol, 1.2 eq.), *exo*-*meso*-2,3-diethylcyclopropane-carbaldehyde (**1b**) (25.2 mg, 200 µmol, 1.0 eq.) and **VI • DCA** (17.8 mg, 40 µmol, 20 mol%) in EtOAc (2.0 mL) were reacted according to GP2 for 20 h at –4 °C. Subsequent reduction with EtOH (2.0 mL) and $NaBH_4$ (37.8 mg, 5.0 eq.) showed full conversion after 30 min at –4 °C. Silica gel column chromatography (*n*-pentane:EtOAc = 15:1) gave the desired products **4b** (19.8 mg, 69.0 µmol, 35%) and the faster eluting **4b'** (19.8 mg, 69.0 µmol, 35%, overall yield 69%) as colorless oils.

(2*R*,3*R*,4*S*)-4-Chloro-3-ethyl-2-(*p*-tolylthio)hexan-1-ol (4b)

^{1}H-NMR (200 MHz, $CDCl_3$): δ = 1.01 (t, *J* = 7.5 Hz, 3 H), 1.10 (t, *J* = 7.2 Hz, 3 H), 1.56–1.84 (m, 3 H), 1.86–2.06 (m, 3 H), 2.33 (s, 3 H), 3.58–3.79 (m, 3 H), 4.21 (ddd, *J* = 10.2, 7.2, 3.2 Hz, 1 H), 7.07–7.16 (m, 2 H), 7.33–7.49 (m, 2 H).

^{13}C-NMR (150 MHz, $CDCl_3$): δ = 11.6, 13.8, 21.1, 21.7, 28.8, 47.3, 56.3, 64.0, 67.9, 129.9, 131.3, 132.7, 137.5.

IR (ATR) $\tilde{\nu}$ (cm^{-1}) = 3404, 2964, 2933, 1492, 1015.

$\boldsymbol{\alpha_D^{24.0}}$ ($CHCl_3$, c = 1.0) = +18.0°.

MS (ESI): *m*/*z* = 309.1 $[M+Na]^+$, 595.2 $[2M+Na]^+$.

$C_{15}H_{23}ClOS$ calcd.: 309.1050

found: 309.1055, $[M+Na]^+$ (ESI-HRMS)

(2*S*,3*R*,4*S*)-4-Chloro-3-ethyl-2-(*p*-tolylthio)hexan-1-ol (4b')

^{1}H-NMR (600 MHz, $CDCl_3$): δ = 0.95 (t, *J* = 7.4 Hz, 3 H), 0.99 (t, *J* = 7.2 Hz, 3 H), 1.58–1.77 (m, 4 H), 2.01 (ddd, *J* = 7.7, 4.9, 4.9 Hz, 1 H), 2.31 (dd, *J* = 7.7, 4.9 Hz, 1 H), 2.34 (s, 3 H), 3.41 (dt, *J* = 7.7, 4.9 Hz, 1 H), 3.62 (ddd, *J* = 11.8, 7.7, 4.9 Hz, 1 H), 3.95 (ddd, *J* = 11.8, 7.7, 4.9 Hz, 1 H), 4.17 (ddd, *J* = 8.9, 4.7, 4.7 Hz, 1 H), 7.11–7.14 (m, 2 H), 7.35–7.38 (m, 2 H).

^{13}C-NMR (150 MHz, $CDCl_3$): δ = 11.8, 13.1, 20.7, 21.1, 28.6, 49.0, 55.7, 62.1, 67.1, 130.0, 130.4, 133.1, 138.0.

IR (ATR) $\tilde{\nu}$ (cm^{-1}) = 3423, 2965, 2934, 1492, 1019.

$\boldsymbol{\alpha_D^{24.0}}$ ($CHCl_3$, c = 1.0) = –5.4°.

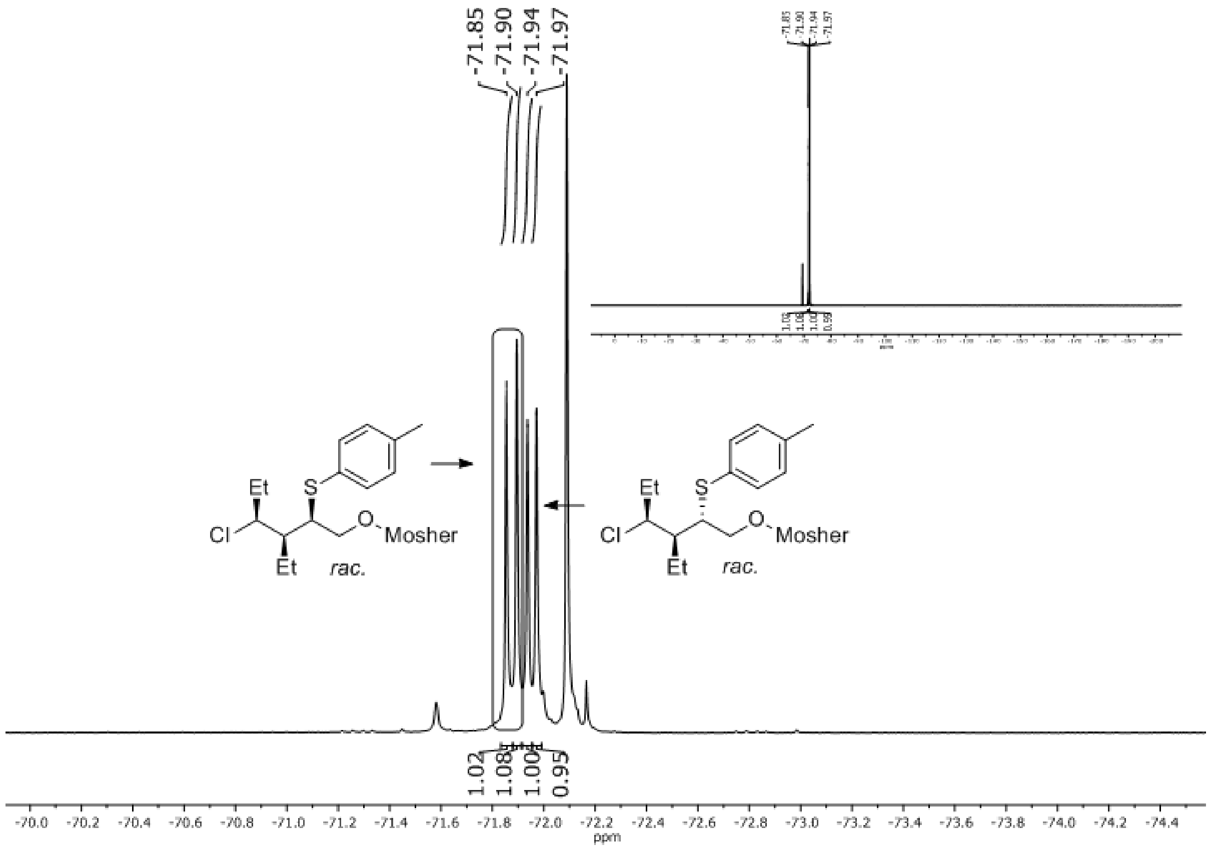

(^{19}F-NMR, 188 MHz, $CDCl_3$; top right: full spectra; the peaks within the box (71.85 and 71.90) belong to **4b**)

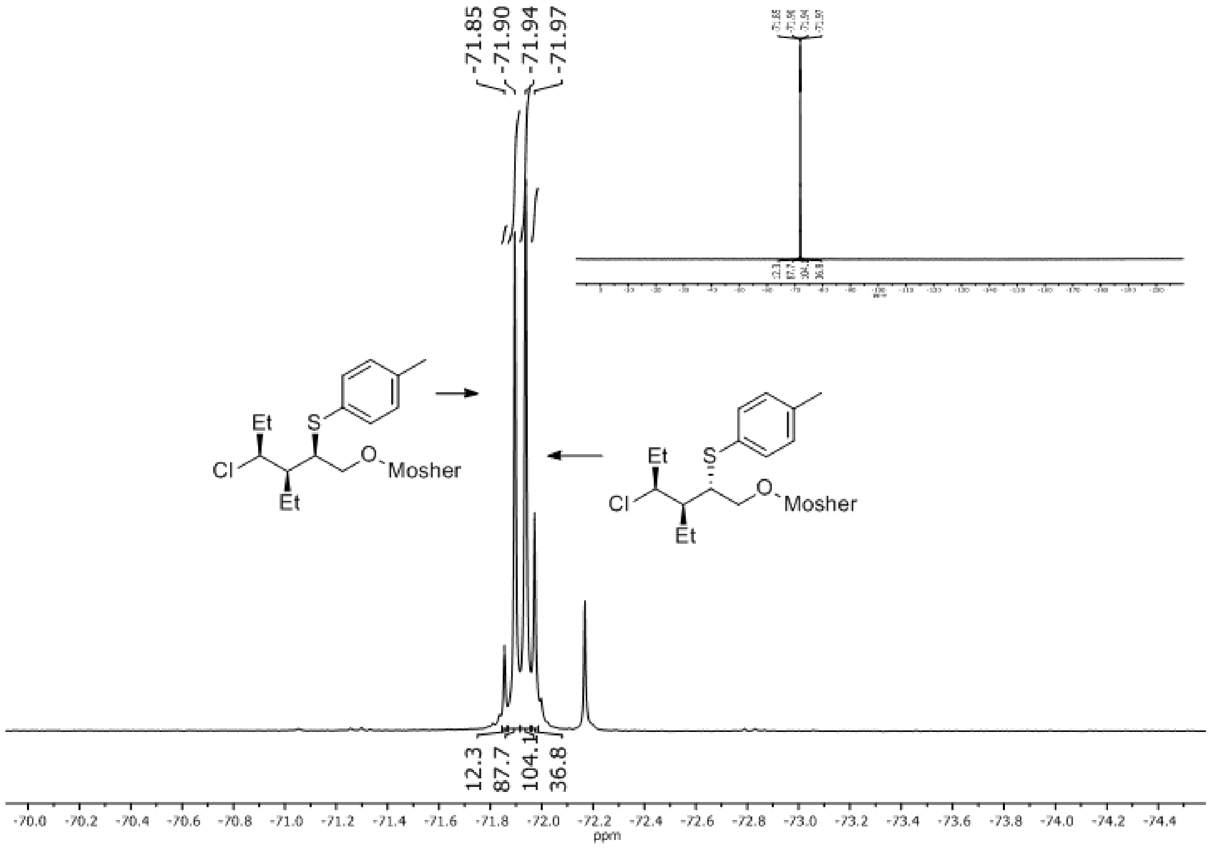

(^{19}F-NMR, 188 MHz, $CDCl_3$; top right: full spectra; e.r. of **4b’** is 73.9:26.1)

(2*R*,3*R*,4*R*)-4-Chloro-3,4-diphenyl-2-(*p*-tolylthio)butan-1-ol (4c)

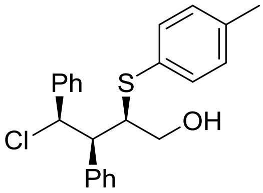

Neat *p*-toylsulfenyl chloride (**2a**) (19.0 mg, 120 µmol, 1.2 eq.), *exo-meso*-2,3-diphenylcyclopropane-carbaldehyde (**1c**) (22.2 mg, 100 µmol, 1.0 eq.) and **IV** (5.4 mg, 20 µmol, 20 mol%) in EtOAc (1.0 mL) were reacted according to GP2 for 5 h at ambient temperature. Subsequent reduction with EtOH (1.0 mL) and $NaBH_4$ (18.9 mg, 5.0 eq.) showed full conversion after 15 min at ambient temperature. Silica gel column chromatography (*n*-pentane:EtOAc = 10:1) gave the desired product **4c** (26.8 mg, 70.0 µmol, 70%, d.r. = 5.2:1) as white solid.

^{1}H-NMR (300 MHz, $CDCl_3$): δ = 3.43 (dd, *J* = 9.8, 9.8 Hz, 1 H), 3.65 (dd, *J* = 11.3, 5.8 Hz, 1 H), 3.97 (dd, *J* = 11.3, 3.5 Hz, 1 H), 4.18 (ddd, *J* = 9.8, 5.8, 3.5 Hz, 1 H), 5.83 (d, *J* = 11.3 Hz, 1 H), 7.05–7.20 (m, 10 H), 7.24–7.31 (m, 2 H), 7.48–7.58 (m, 2 H).
^{13}C-NMR (75 MHz, $CDCl_3$): δ = 21.1, 53.0, 55.2, 64.2, 64.8, 127.3, 127.9, 128.0, 128.1, 128.2, 129.5, 129.9, 131.3, 133.0, 136.4, 137.7, 140.2.
IR (ATR) $\tilde{\nu}$ (cm^{-1}) = 3412, 2923, 1711, 1492, 1000.
$\boldsymbol{\alpha_D^{24.0}}$ ($CHCl_3$, c = 2.0) = +12.0°.
MS (ESI): *m*/*z* = 405.1 $[M+Na]^+$.
$C_{23}H_{23}ClOS$ calcd.: 405.1050
found: 405.1053, $[M+Na]^+$ (ESI-HRMS)

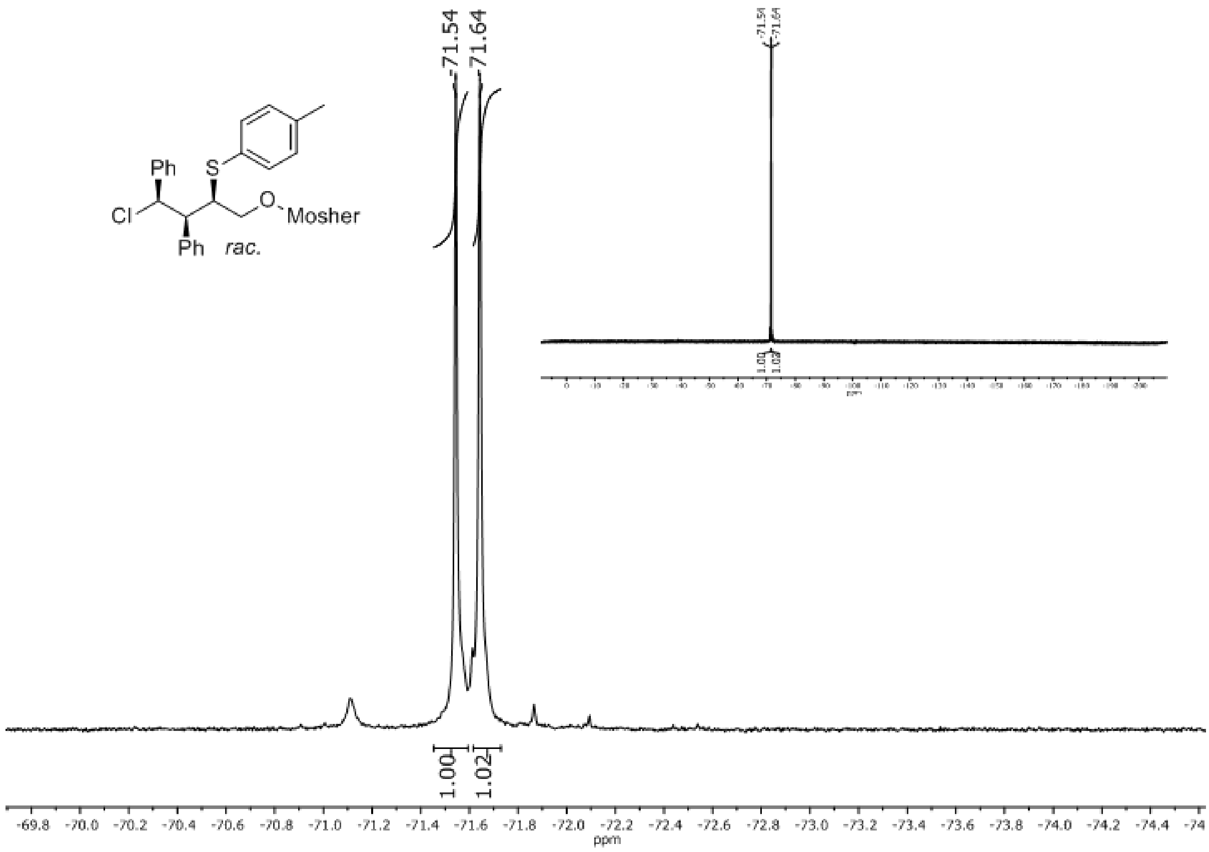

(^{19}F-NMR, 188 MHz, $CDCl_3$; top right: full spectra)

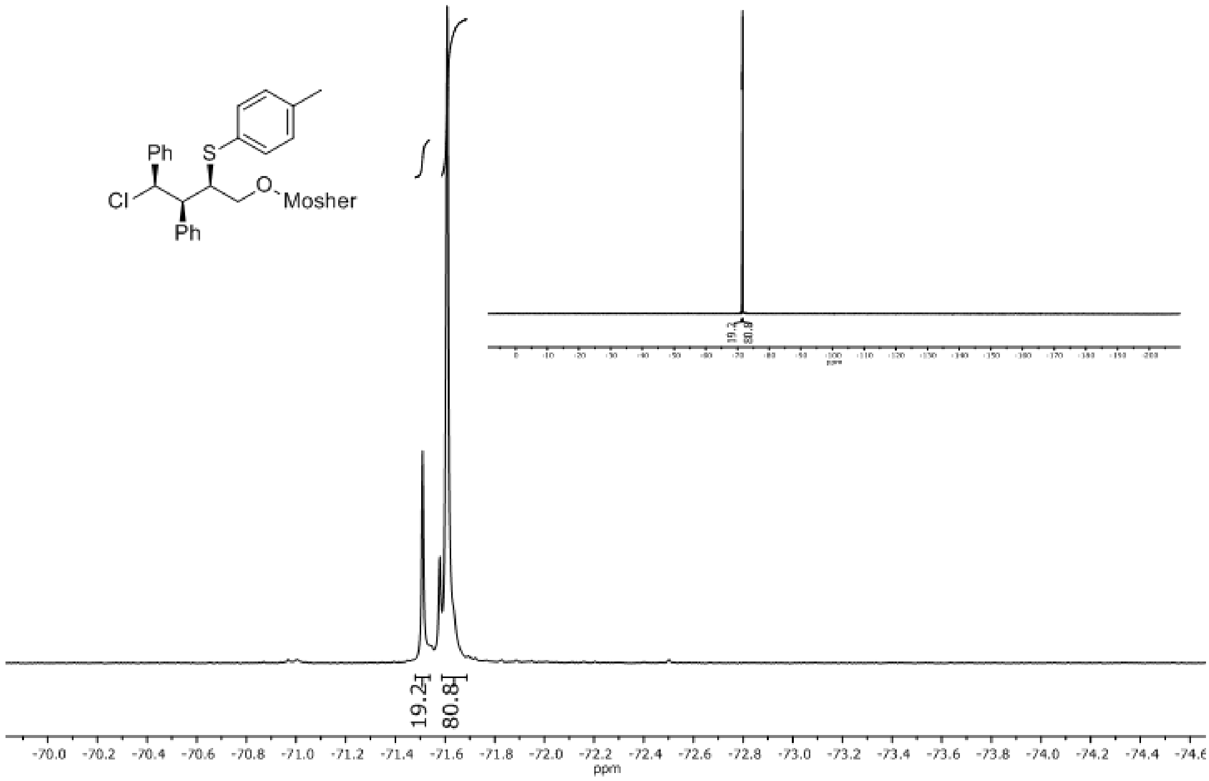

(^{19}F-NMR, 188 MHz, $CDCl_3$; top right: full spectra)

(*R*)-2-((3*R*,4*R*)-4-Chlorotetrahydrofuran-3-yl)-2-(*p*-tolylthio)ethanol (4d)

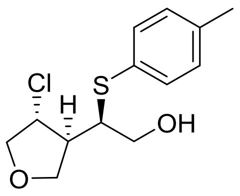

Neat *p*-toylsulfenyl chloride (**2a**) (19.0 mg, 120 µmol, 1.2 eq.), *exo*-*meso*-3-oxabicyclo[3.1.0]hexane-6-carbaldehyde (**1d**) (11.2 mg, 100 µmol, 1.0 eq.) and **V • DCA** (7.9 mg, 20 µmol, 20 mol%) in DME (1.0 mL) were reacted according to GP2 for 72 h at ambient temperature. Subsequent reduction with EtOH (1.0 mL) and $NaBH_4$ (18.9 mg, 5.0 eq.) showed full conversion after 15 min at ambient temperature. Silica gel column chromatography (*n*-pentane:EtOAc = 4:1) gave the desired product **4d** (17.8 mg, 65.2 µmol, 65%, d.r. = 2.1:1) as colorless oil.

^{1}H-NMR (300 MHz, $CDCl_3$): δ = 2.28 (dd, *J* = 6.8, 6.8 Hz, 1 H), 2.34 (s, 3 H), 2.67–2.77 (m, 1 H), 3.07 (ddd, *J* = 8.5, 6.1, 5.1 Hz, 1 H), 3.59 (ddd, *J* = 12.0, 6.1, 6.1 Hz, 1 H), 3.73 (ddd, *J* = 12.0, 7.1, 5.1 Hz, 1 H), 3.80 (dd, *J* = 9.3, 5.1 Hz, 1 H), 3.92 (dd, *J* = 10.3, 3.6 Hz, 1 H), 4.11 (dd, *J* = 9.3, 7.1 Hz, 1 H), 4.17 (dd, *J* = 10.3, 5.8 Hz, 1 H), 4.64 (ddd, *J* = 5.8, 3.6, 3.6 Hz, 1 H), 7.12–7.17 (m, 2 H), 7.35– 7.40 (m, 2 H).

^{13}C-NMR (75 MHz, $CDCl_3$): δ = 21.1, 51.2, 54.3, 59.7, 62.2, 70.3, 75.8, 128.6, 130.1, 133.6, 138.5.

IR (ATR) $\tilde{\nu}$ (cm^{-1}) = 3425, 2924, 2867, 1687, 1066.

$\boldsymbol{\alpha_D^{24.0}}$ ($CHCl_3$, c = 2.0) = –4.0°.

MS (ESI): *m*/*z* = 295.1 [M+Na]$^+$.

$C_{13}H_{17}ClO_2S$ calcd.: 295.0530

found: 295.0531, [M+Na]$^+$ (ESI-HRMS)

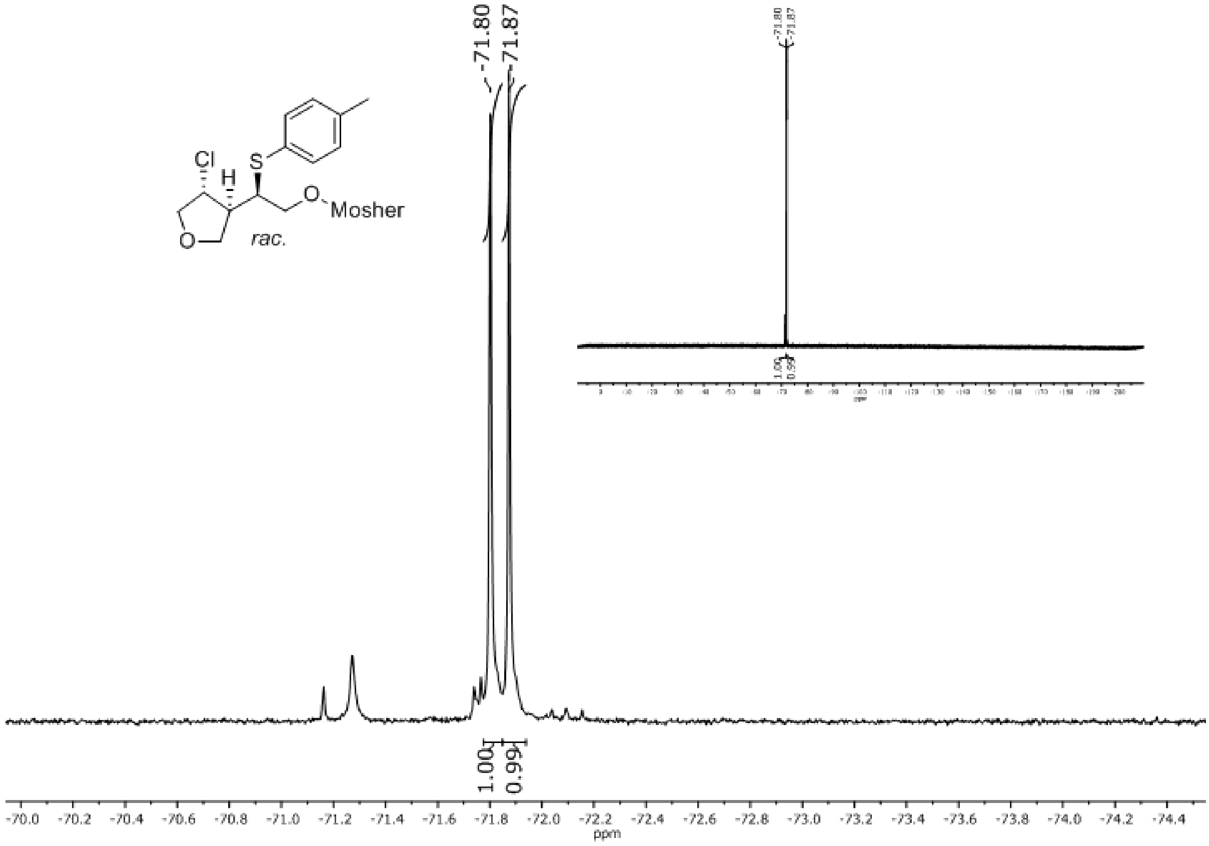

(^{19}F-NMR, 188 MHz, $CDCl_3$; top right: full spectra)

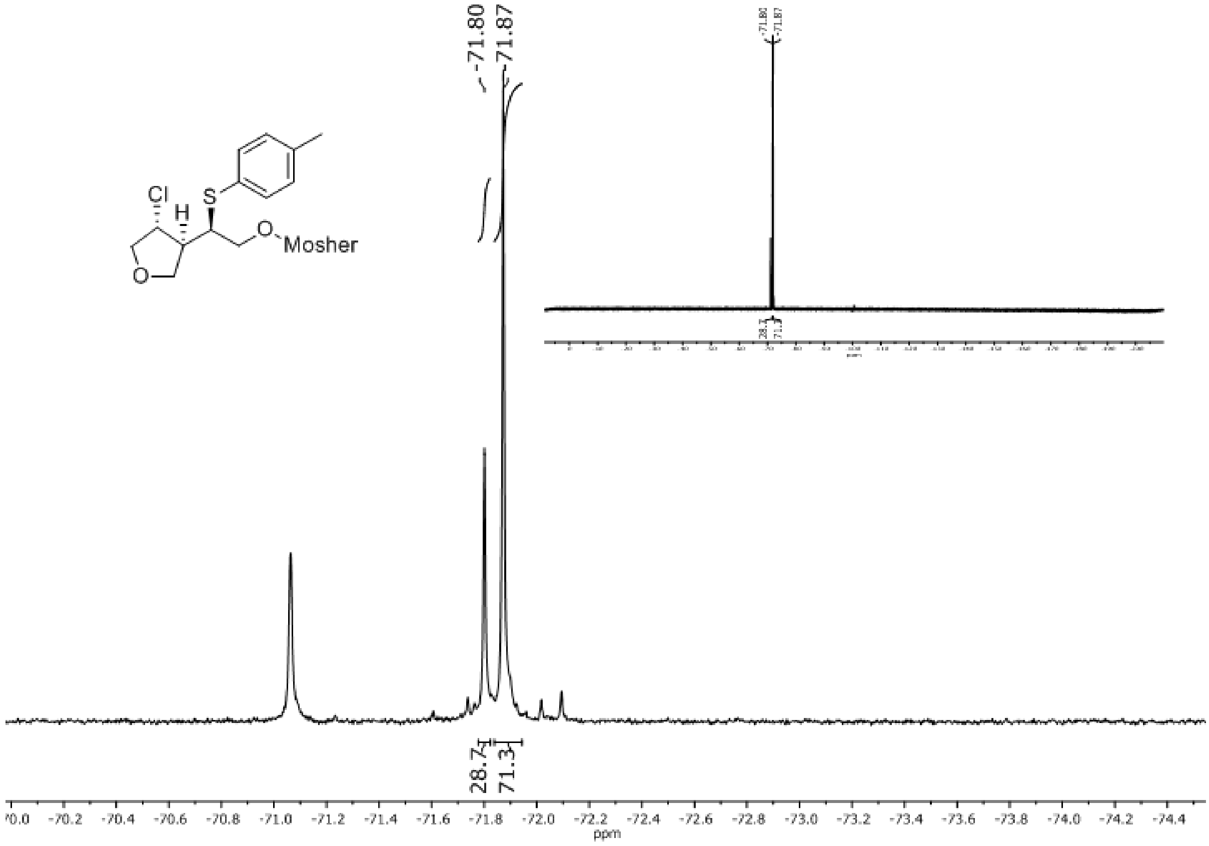

(^{19}F-NMR, 188 MHz, $CDCl_3$; top right: full spectra)

(*R*)-2-((3*R*,4*R*)-4-Chloro-*N*-Boc-pyrroldin-3-yl)-2-(*p*-tolylthio)ethanol (4e)

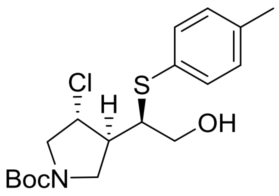

Neat *p*-toylsulfenyl chloride (**2a**) (19.0 mg, 120 µmol, 1.2 eq.), *exo*-*meso*-3-*N*-Boc-azabicyclo[3.1.0]hexane-6-carbaldehyde (**1e**) (21.1 mg, 100 µmol, 1.0 eq.) and **VI** (6.3 mg, 20 µmol, 20 mol%) in DME (2.0 mL) were reacted according to GP2 for 72 h at ambient temperature. Subsequent reduction with EtOH (2.0 mL) and $NaBH_4$ (18.9 mg, 5.0 eq.) showed full conversion after 15 min at ambient temperature. Silica gel column chromatography (*n*-pentane:EtOAc = 4:1) gave the desired product **4e** (17.9 mg, 48.1 µmol, 48%, d.r. = 1.6:1) as colorless oil (mixture of diastereomeres, no sepearation on column possible).

^{1}H-NMR (300 MHz, $CDCl_3$): δ = 1.46 (s) and 1.47 (s, together 9 H), 2.17–2.38 (m, 1 H) 2.34 (s, 3 H), 2.56–2.71 (m, 1 H), 2.99–3.12 (m) and 3.27 (dd, *J* = 10.5, 10.5 Hz, together 1 H), 3.32–3.57 (m, 2 H), 3.59–4.05 (m, 4 H), 4.49 (ddd, *J* = 8.3, 8.3, 8.3 Hz) and 4.72 (ddd, *J* = 5.9, 4.5, 4.5 Hz, together 1 H), 7.09–7.19 (m, 2 H), 7.34–7.41 (m, 2 H).

^{13}C-NMR (75 MHz, $CDCl_3$): δ = 21.1, 28.4, 53.4, 53.5, 64.1, 77.2, 77.2, 80.0, 80.0, 130.1, 130.2, 133.0, 133.5, 153.9 (^{13}C-NMR shows just one set of signals).

MS (ESI): *m*/*z* = 394.1 $[M+Na]^+$, 765.3 $[2M+Na]^+$.

$C_{18}H_{26}ClO_3NS$ calcd.: 394.1214

found: 394.1215, $[M+Na]^+$ (ESI-HRMS)

Ferrocenyl Ester Adduct 5

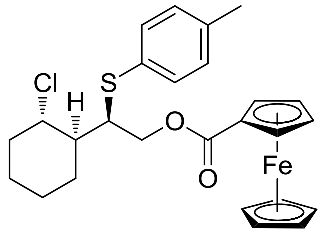

4-DMAP (18.3 mg, 150 µmol, 1.5 eq.) was added to a solution of (*R*)-2-((1*R*,2*S*)-2-Chlorocyclohexyl)-2-(*p*-tolylthio)ethanol (**3a**) (28.4 mg, 100 µmol, 1.0 eq.) and ferrocene carboxylic acid chloride (30.0 mg, 120 µmol, 1.2 eq.) in CH_2Cl_2 (1.0 mL) and the resulting mixture was stirred for 2 h at ambient temperature. Sat. aq. NH_4Cl-solution (1.0 mL) was added to the mixture and the aqueous phase was extracted with CH_2Cl_2 (5.0 mL). The combined organic phases were washed with sat. aq. $NaHCO_3$-solution (2.0 mL) and dried over Na_2SO_4. Silica gel column chromatography (*n*-pentane:EtOAc = 80:1) gave the desired product **5** (37.5 mg, 75.5 µmol, 76%) as orange solid.

^{1}H-NMR (600 MHz, $CDCl_3$): δ = 1.24–1.43 (m, 3 H), 1.73–1.85 (m, 3 H), 2.01 (ddt, *J* = 9.9, 6.4, 2.4 Hz, 1 H), 2.05–2.10 (m, 1 H), 2.33 (s, 3 H), 2.36–2.41 (m, 1 H), 4.06 (ddd, *J* = 10.8, 5.5, 2.4 Hz, 1 H), 4.19 (s, 5 H), 4.33 (dd, *J* = 10.8, 5.5 Hz, 1 H), 4.38 (dd, *J* = 2.4, 1.7 Hz, 2 H), 4.39–4.44 (m, 2 H), 4.71–4.78 (m, 2 H), 7.08–7.17 (m, 2 H), 7.44–7.52 (m, 2 H).

^{13}C-NMR (150 MHz, $CDCl_3$): δ = 21.1, 25.4, 26.3, 26.4, 37.7, 46.3, 50.7, 62.7, 63.9, 69.8, 70.1, 70.2, 70.8, 71.3, 129.9, 131.1, 132.7, 137.5, 171.4.

IR (ATR) $\tilde{\nu}$ (cm^{-1}) = 2939, 1713, 1460, 1273, 1146.

$\boldsymbol{\alpha_D^{20.8}}$ ($CHCl_3$, c = 2.0) = –52.5°.

MS (ESI): *m*/*z* = 496.1 [M+Na]$^+$.

$C_{26}H_{29}ClFeO_2S$ calcd.: 496.0921

found: 496.0915, [M]$^+$ (ESI-HRMS)

^{1}H-, ^{13}C- and ^{19}F-NMR-Spectra of all New Compounds

(2*R*,5*S*)-(–)-5-*tert*-butyl-3-methyl-2-(5-methylanfuran-2-yl)-4-imidazolidinone (IV')

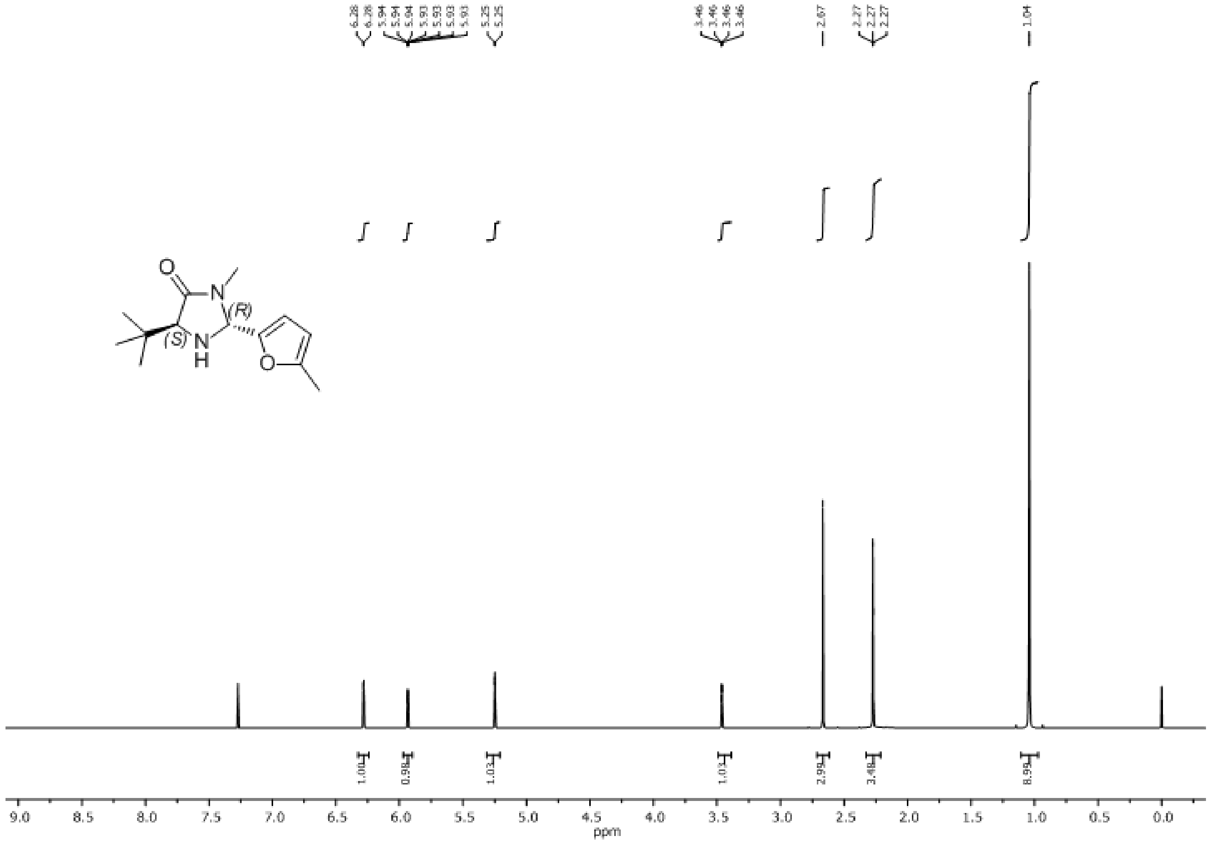

(^{1}H-NMR, 600 MHz, $CDCl_3$)

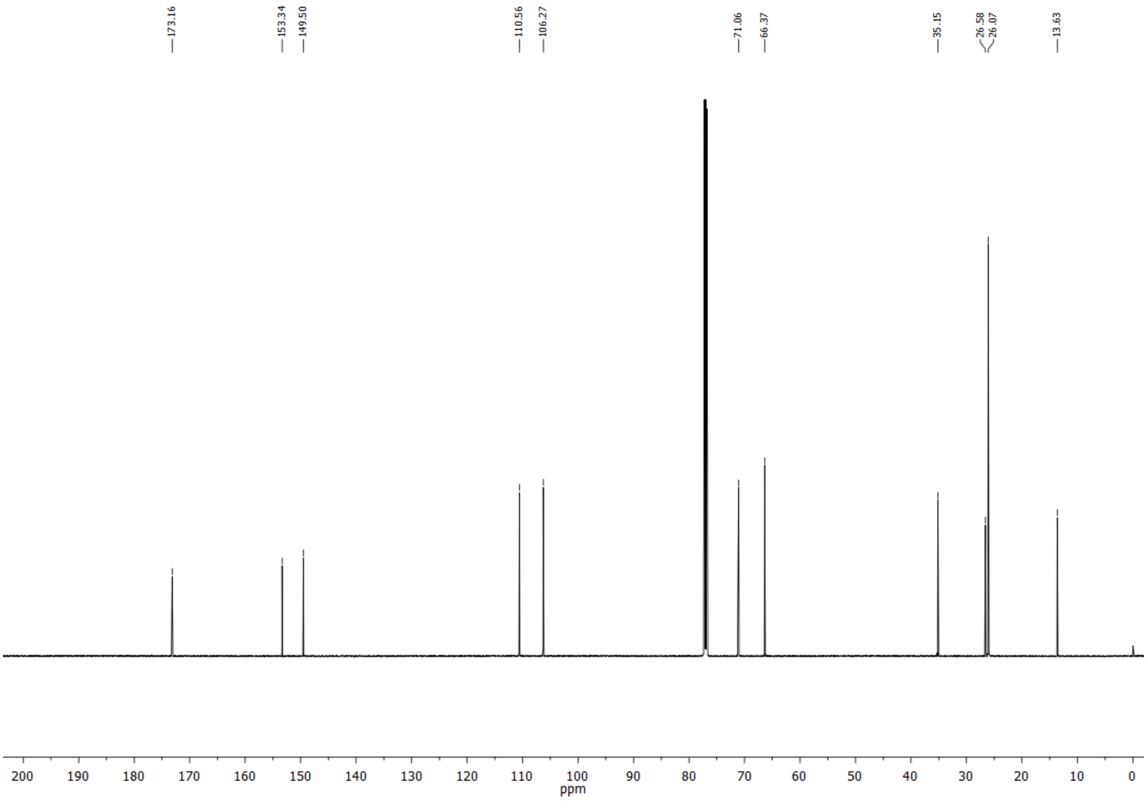

(^{13}C-NMR, 150 MHz, $CDCl_3$)

(2*R*,5*S*)-(–)-5-*tert*-butyl-3-methyl-2-(5-methylanfuran-2-yl)-4-imidazolidinone (IV)

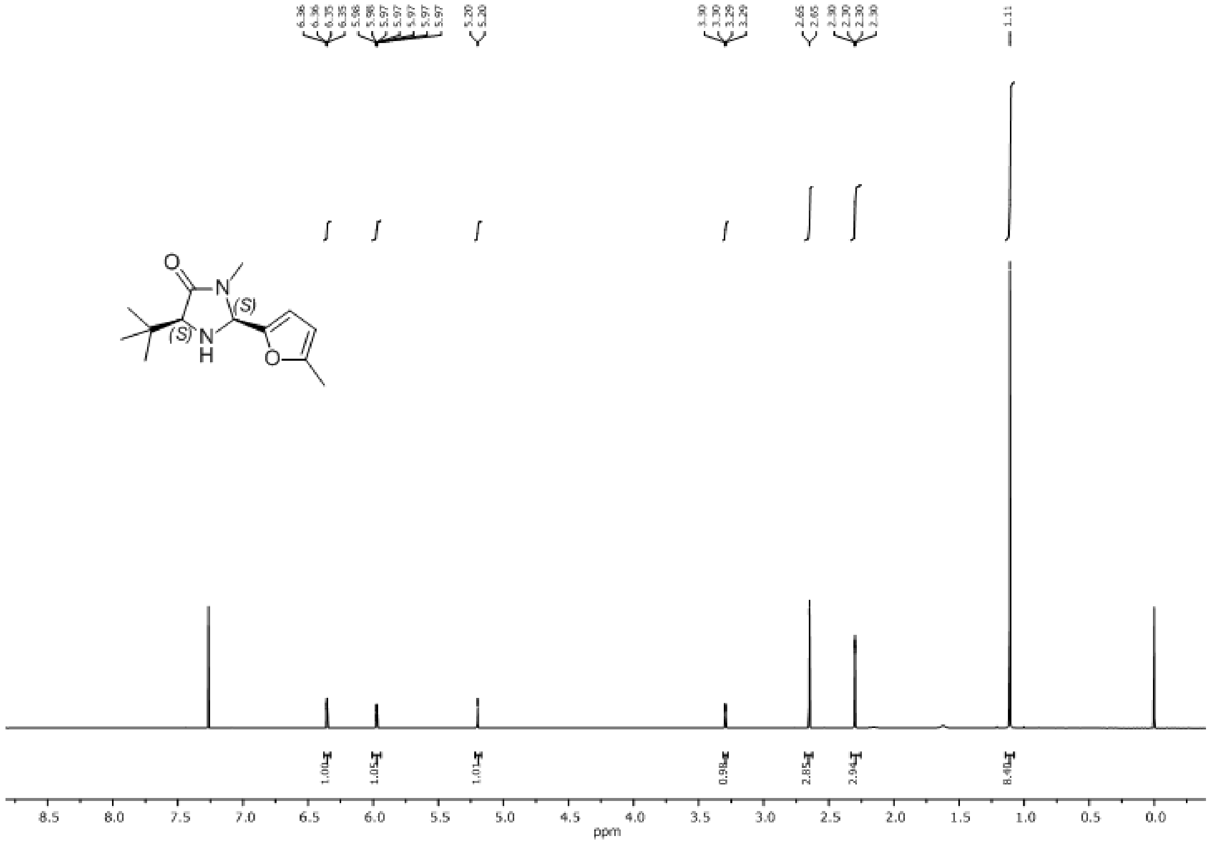

(^{1}H-NMR, 600 MHz, $CDCl_3$)

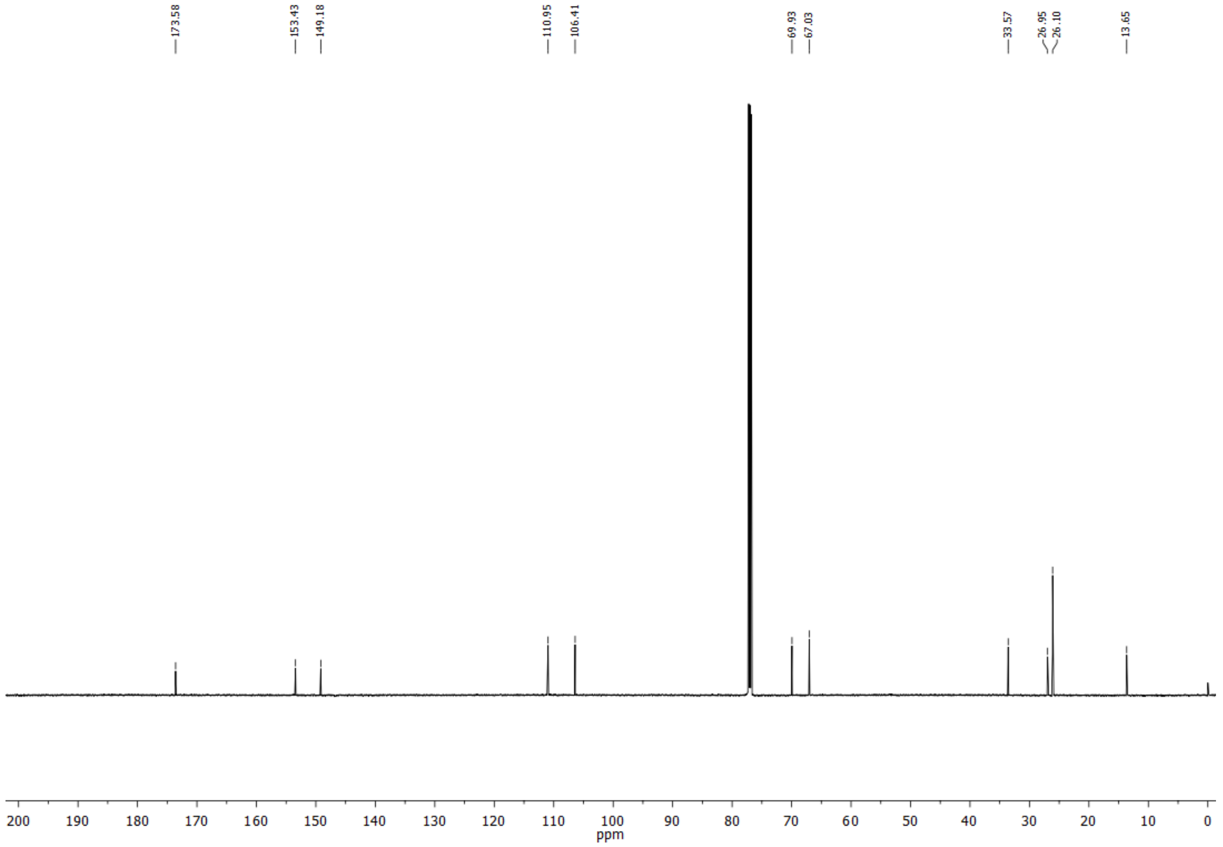

(^{13}C-NMR, 150 MHz, $CDCl_3$)

(2*R*,5*S*)-(–)-5-Benzyl-3-methyl-2-(1-naphthyl)-4-imidazolidinone (VI')

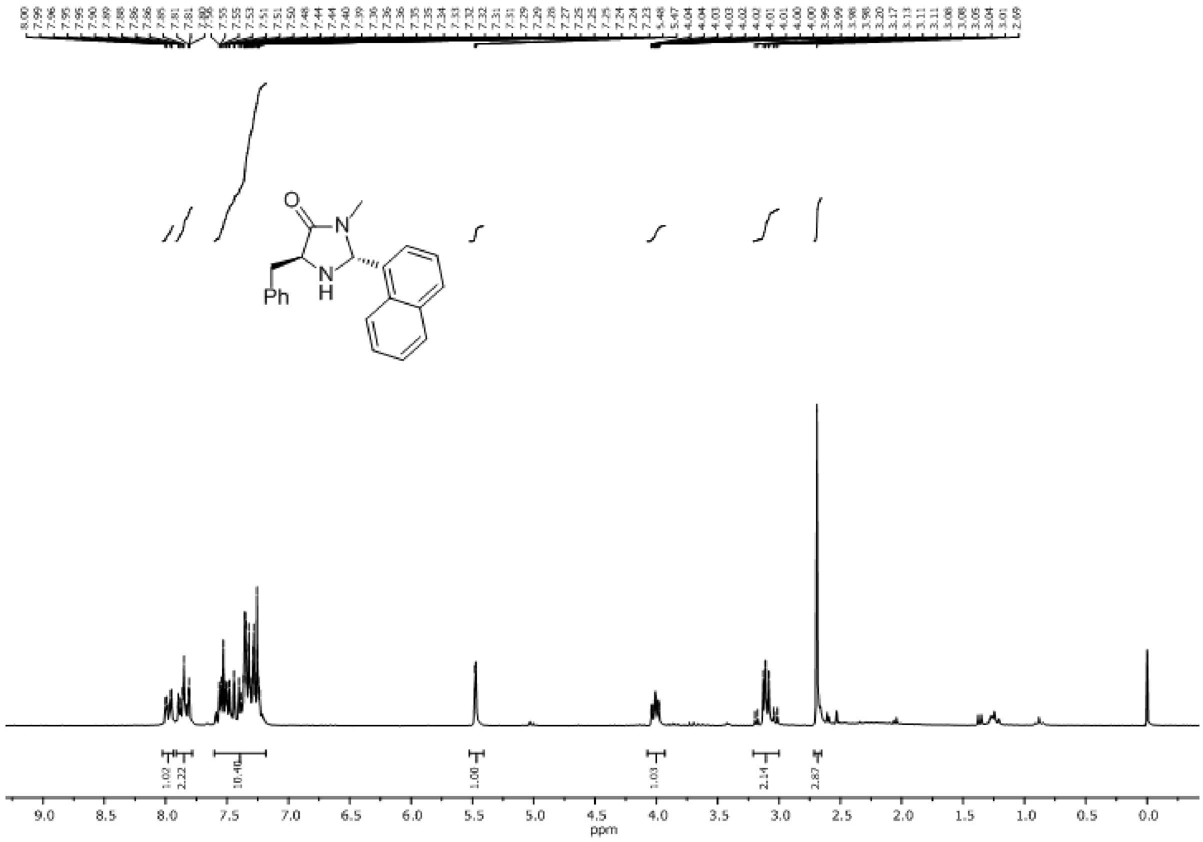

(^{1}H-NMR, 200 MHz, $CDCl_3$)

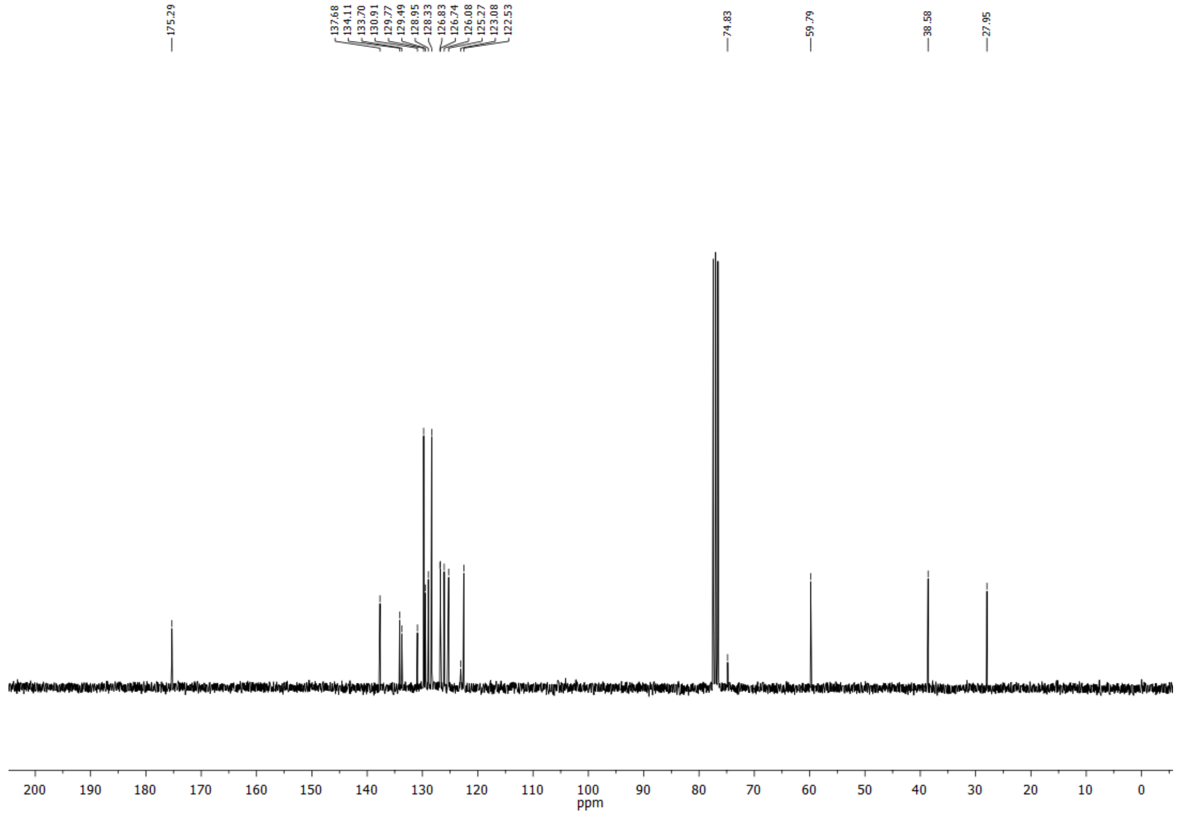

(^{13}C-NMR, 75 MHz, $CDCl_3$)

(2*S*,5*S*)-(–)-5-Benzyl-3-methyl-2-(1-naphthyl)-4-imidazolidinone (VI)

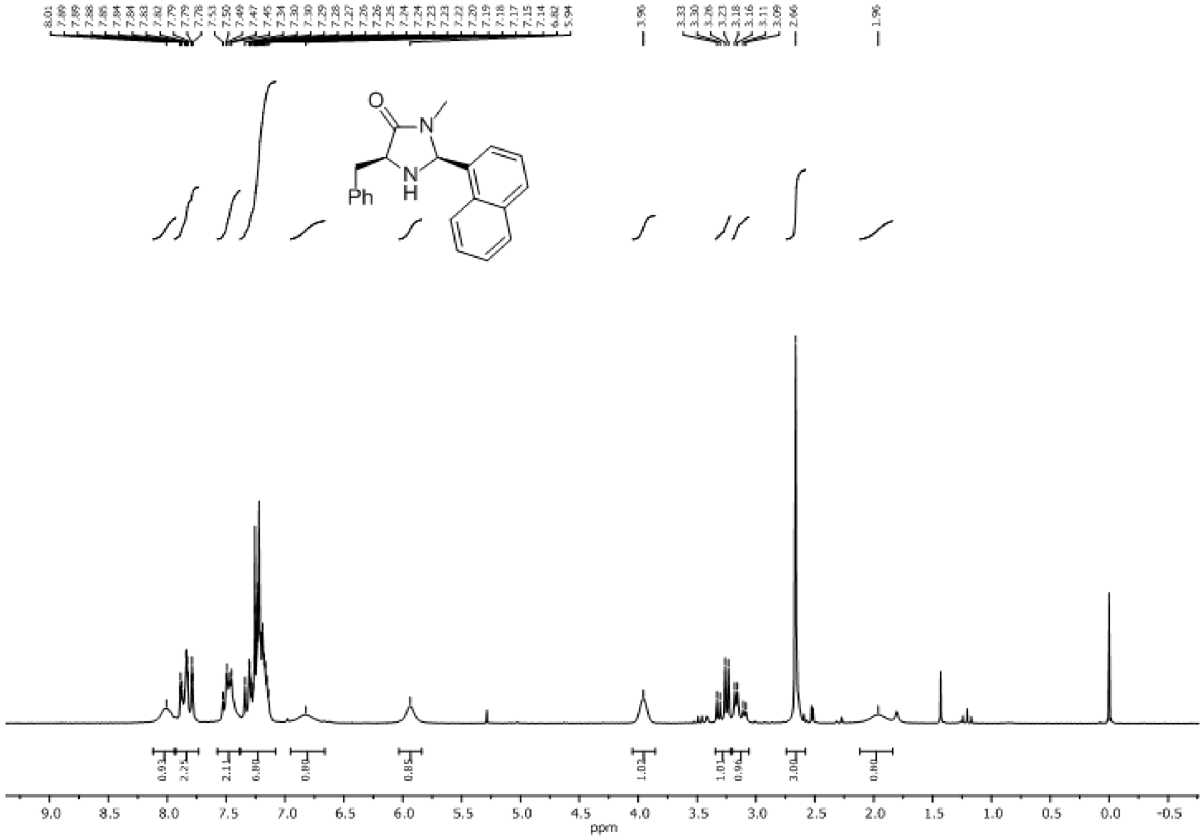

(^{1}H-NMR, 200 MHz, $CDCl_3$)

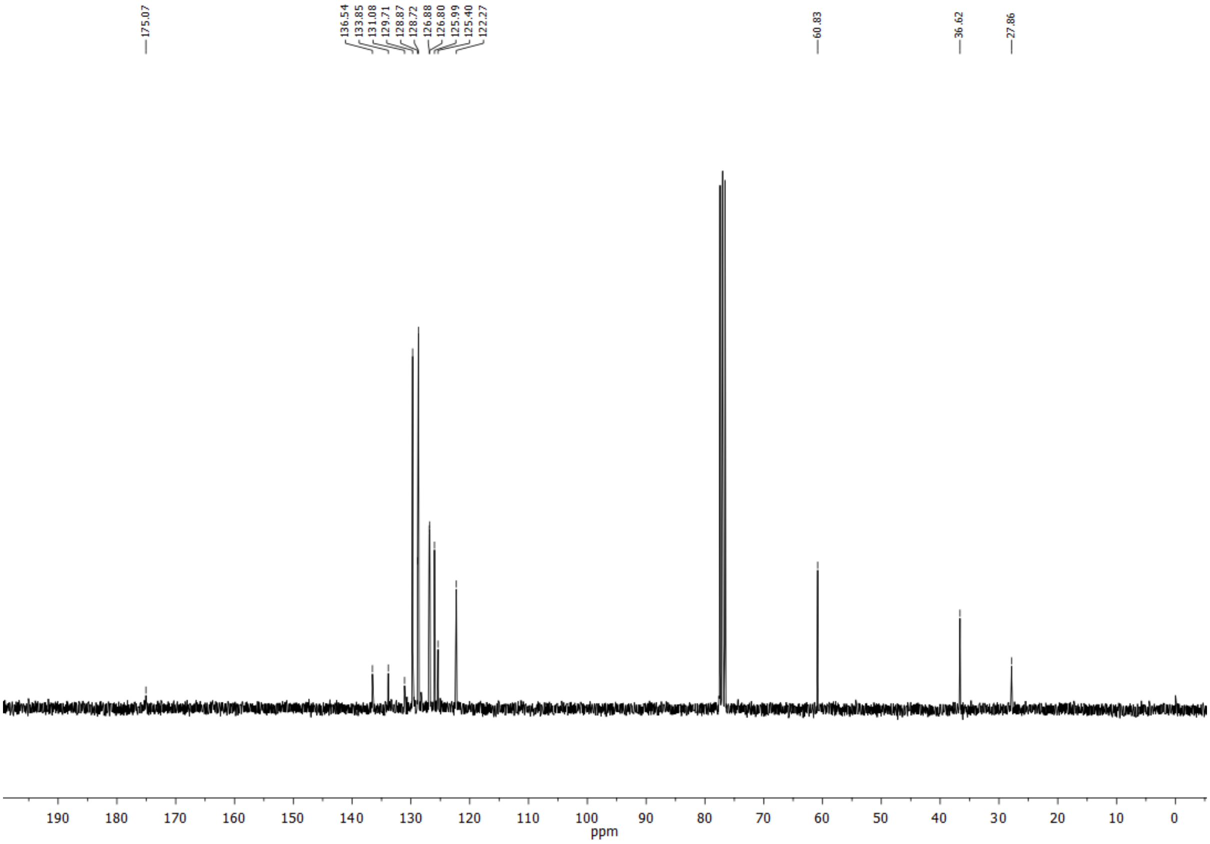

(^{13}C-NMR, 75 MHz, $CDCl_3$)

(2*S*,5*S*)-(+)-5-Benzyl-3-methyl-2-propyl-4-imidazolidinone (XX)

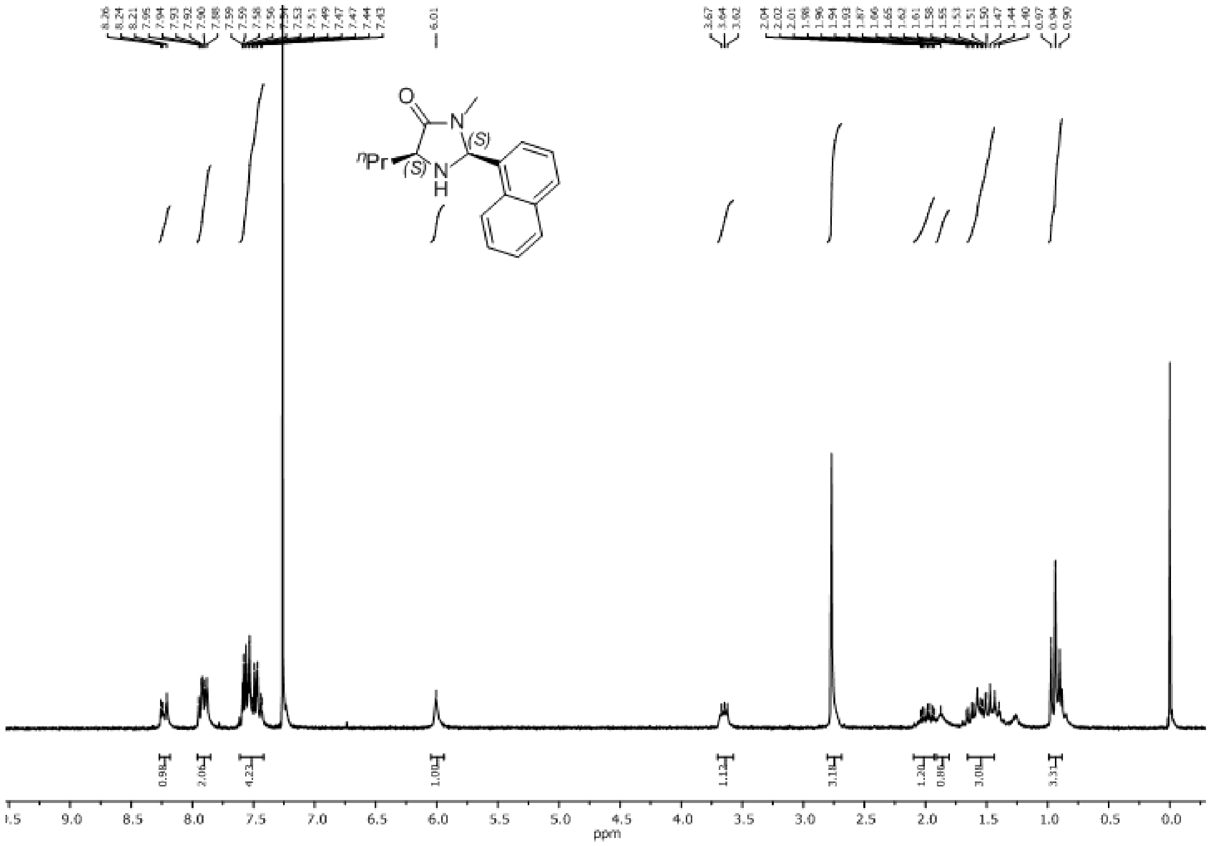

(^{1}H-NMR, 200 MHz, $CDCl_3$)

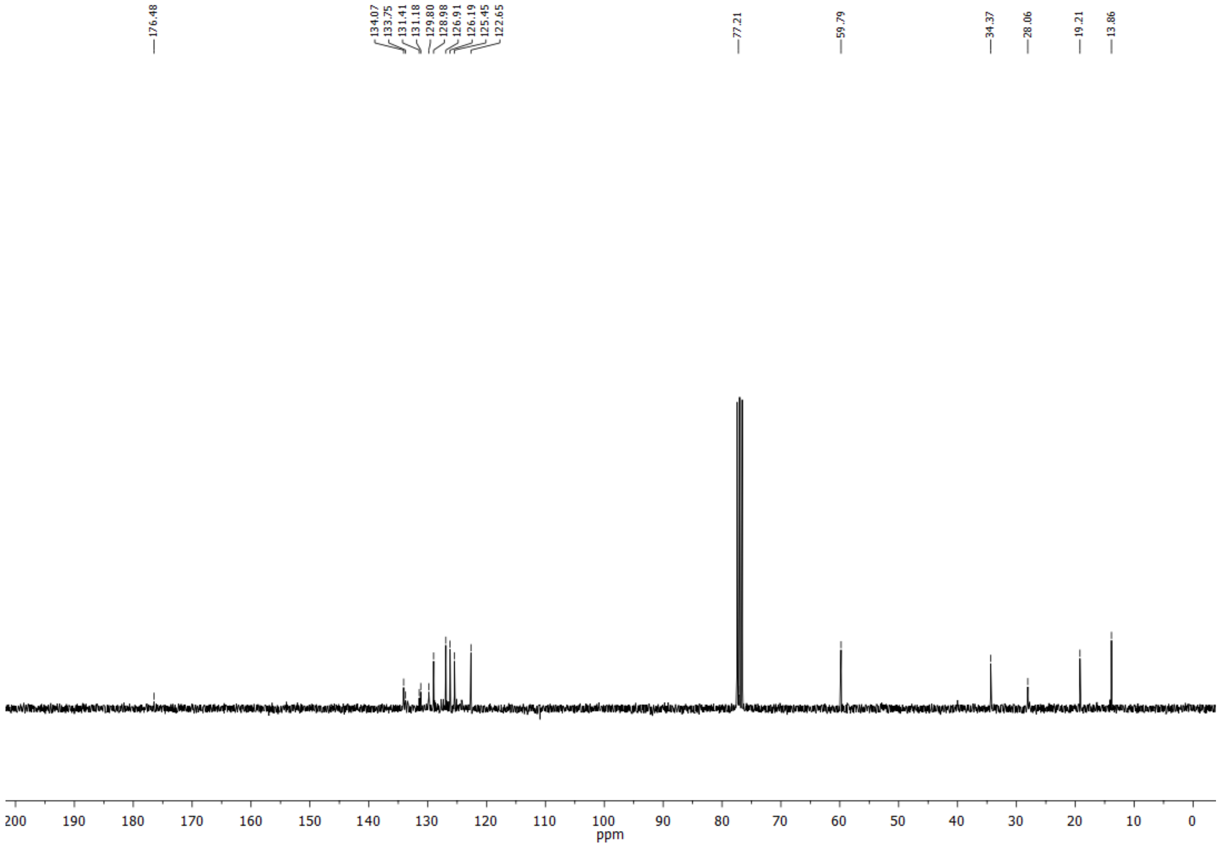

(^{13}C-NMR, 75 MHz, $CDCl_3$)

(2*S*,5*S*)-(–)-5-Benzyl-3-methyl-2-(3,5-di-*tert*-butyl-phen-1-yl)-4-imidazolidinone (VII)

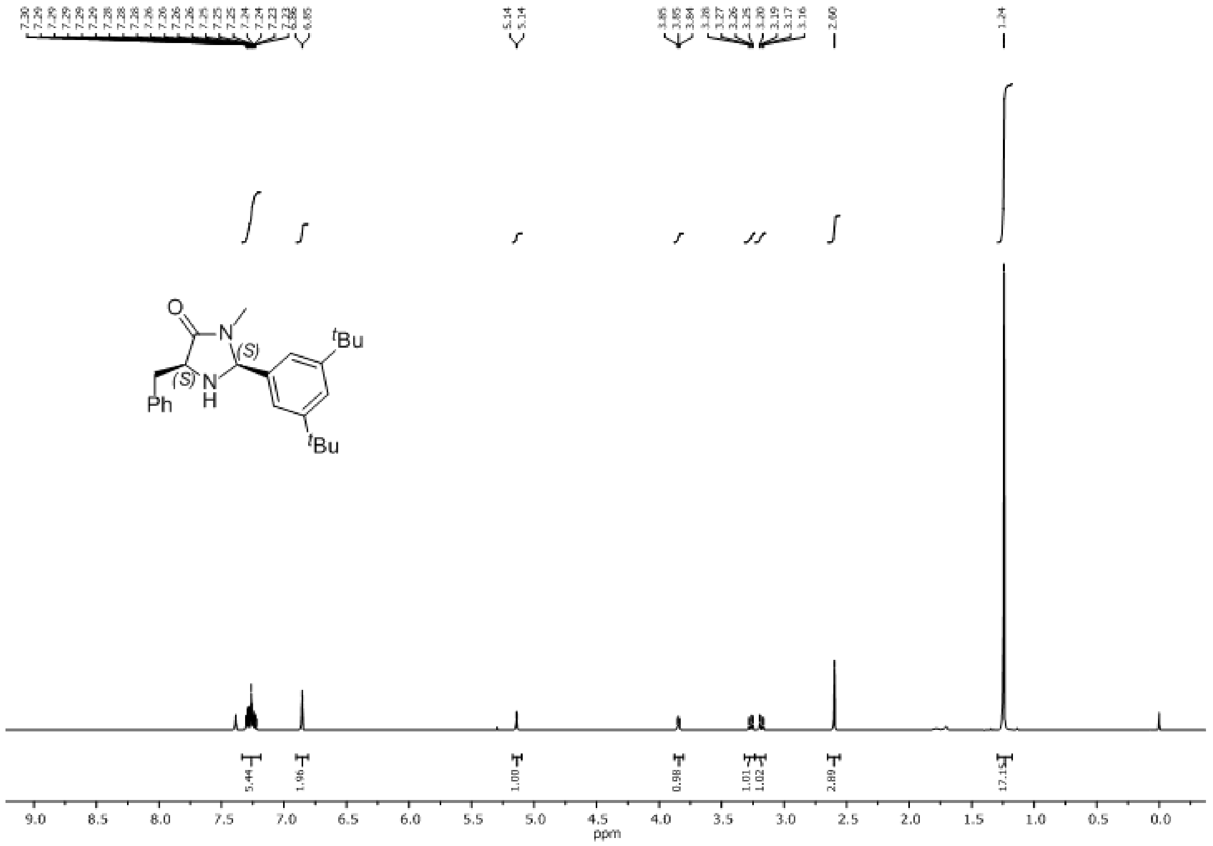

(^{1}H-NMR, 600 MHz, $CDCl_3$)

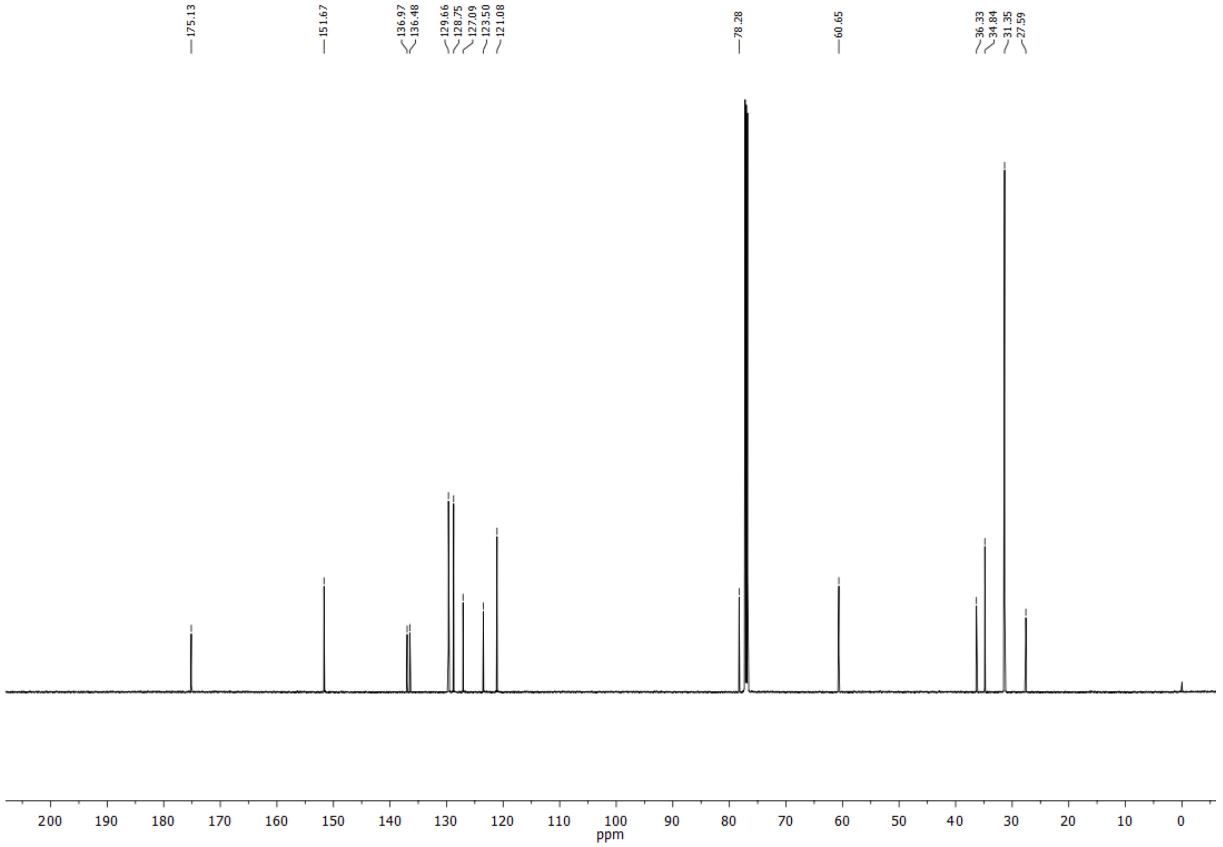

(^{13}C-NMR, 150 MHz, $CDCl_3$)

(*R*)-2-((1*R*,2*S*)-2-Chlorocyclohexyl)-2-(*p*-tolylthio)ethanol (3a)

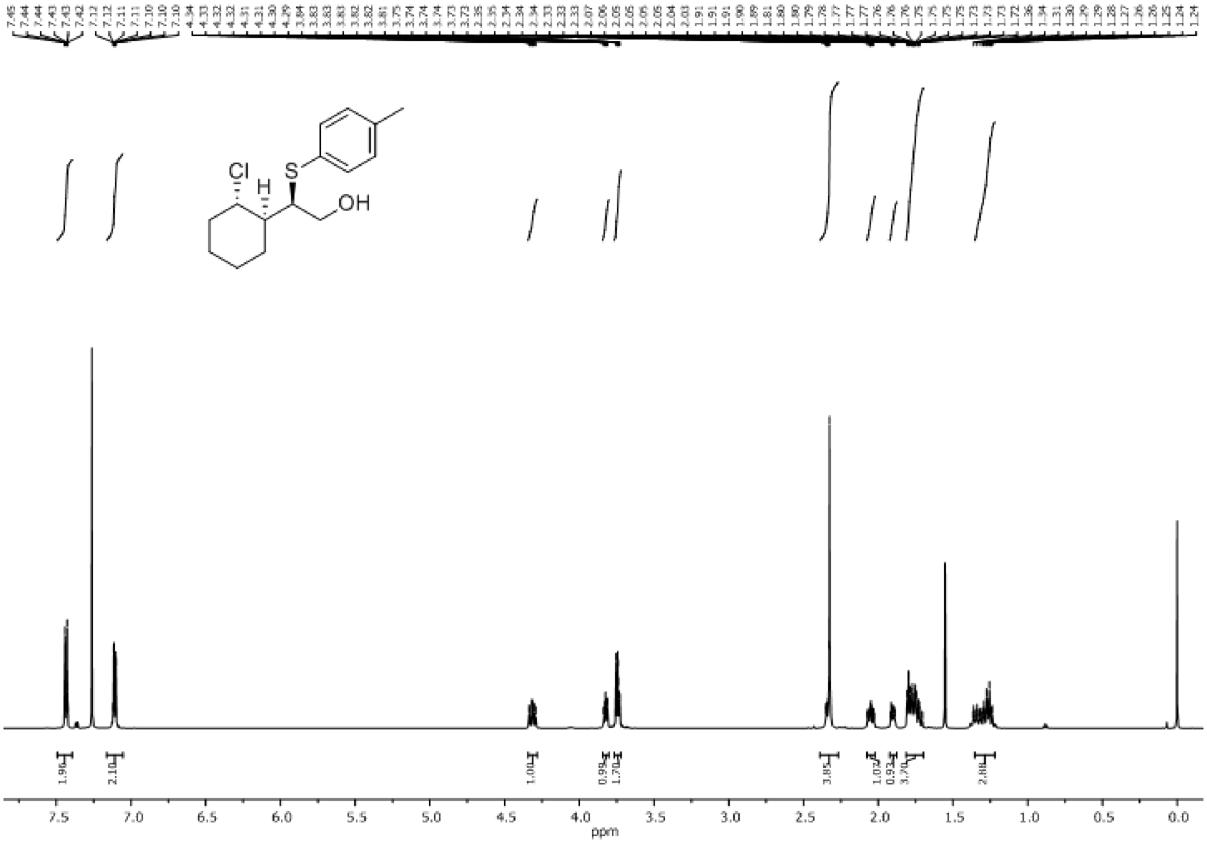

(^{1}H-NMR, 600 MHz, $CDCl_3$)

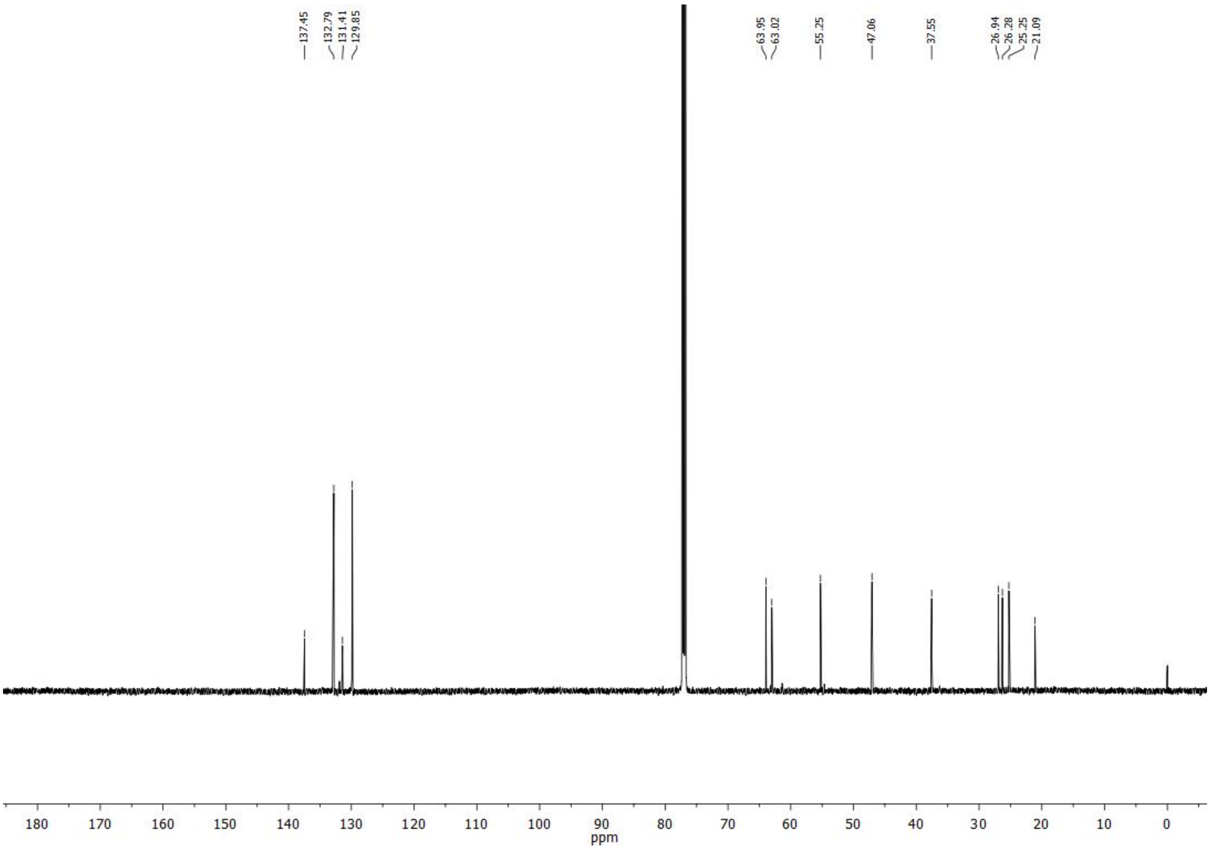

(^{13}C-NMR, 150 MHz, $CDCl_3$)

(*R*)-2-((1*R*,2*S*)-2-Chlorocyclohexyl)-2-(*p*-anisolethio)ethanol (3b)

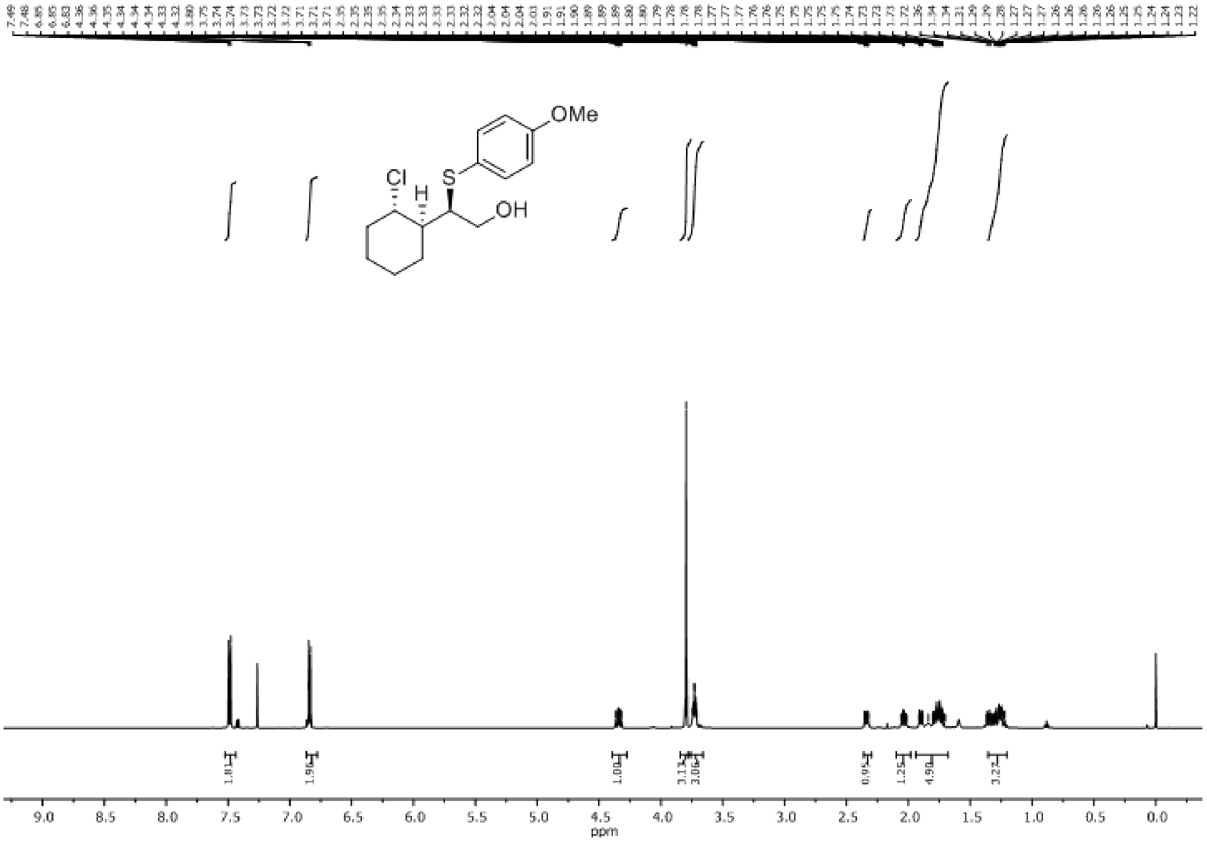

(^{1}H-NMR, 600 MHz, $CDCl_3$)

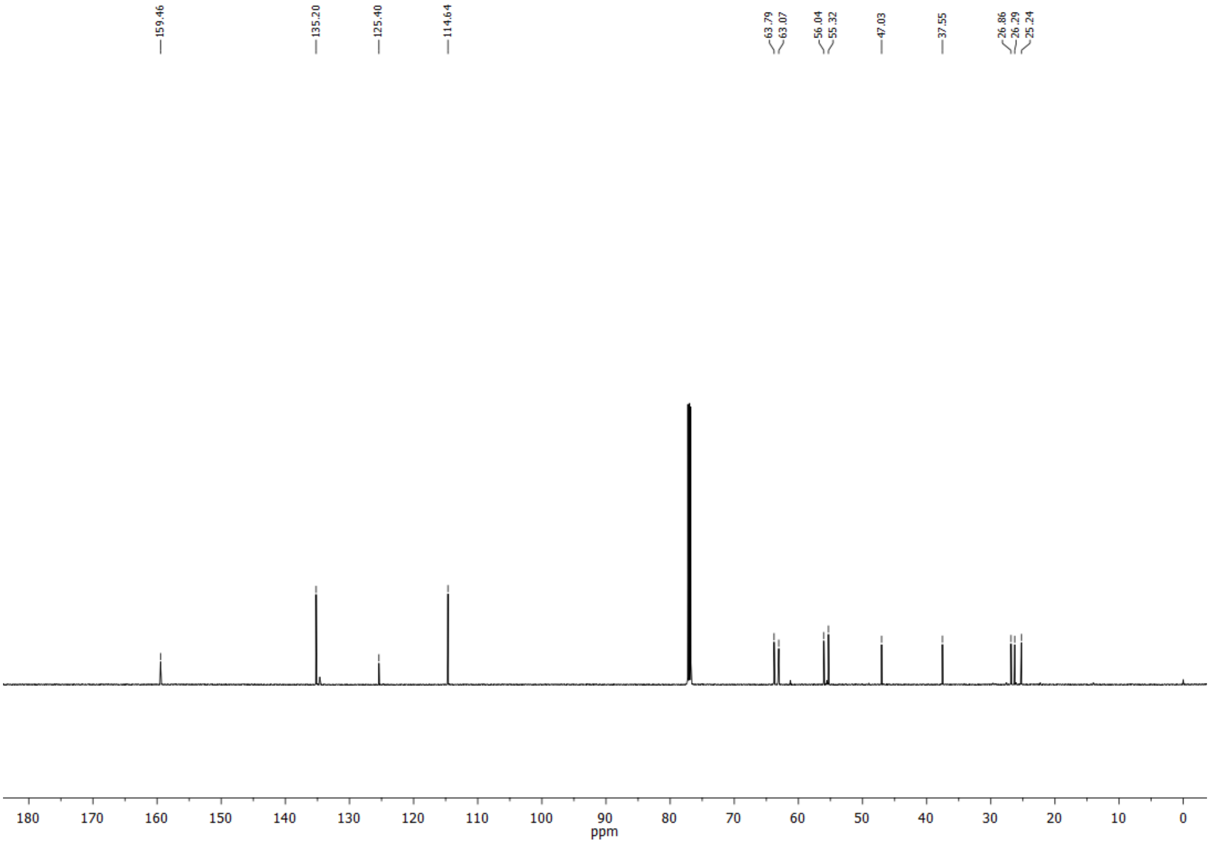

(^{13}C-NMR, 150 MHz, $CDCl_3$)

(*R*)-2-((1*R*,2*S*)-2-Chlorocyclohexyl)-2-(*p*-bromophenylthio)ethanol (3c)

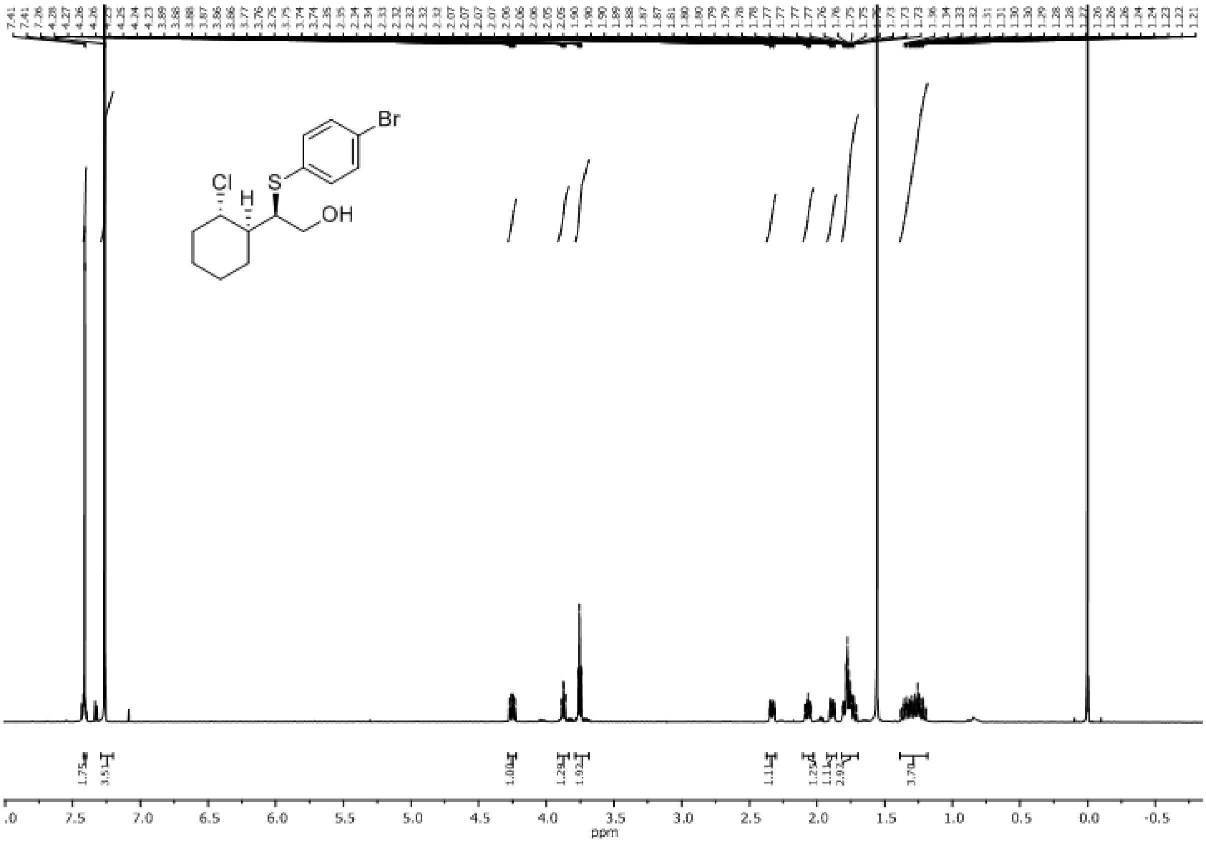

(^{1}H-NMR, 600 MHz, $CDCl_3$)

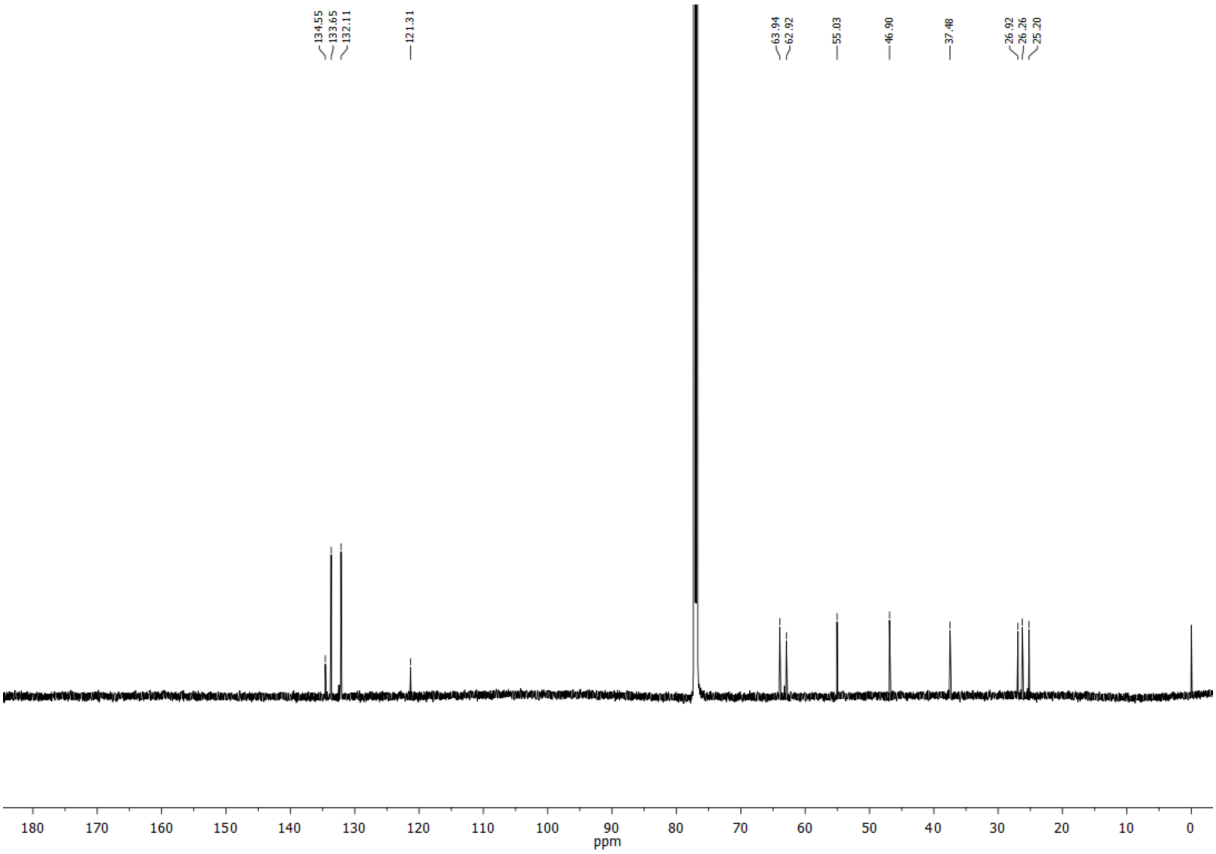

(^{13}C-NMR, 150 MHz, $CDCl_3$)

(*R*)-2-((1*R*,2*S*)-2-Chlorocyclohexyl)-2-(*p*-nitrophenylthio)ethanol (3d)

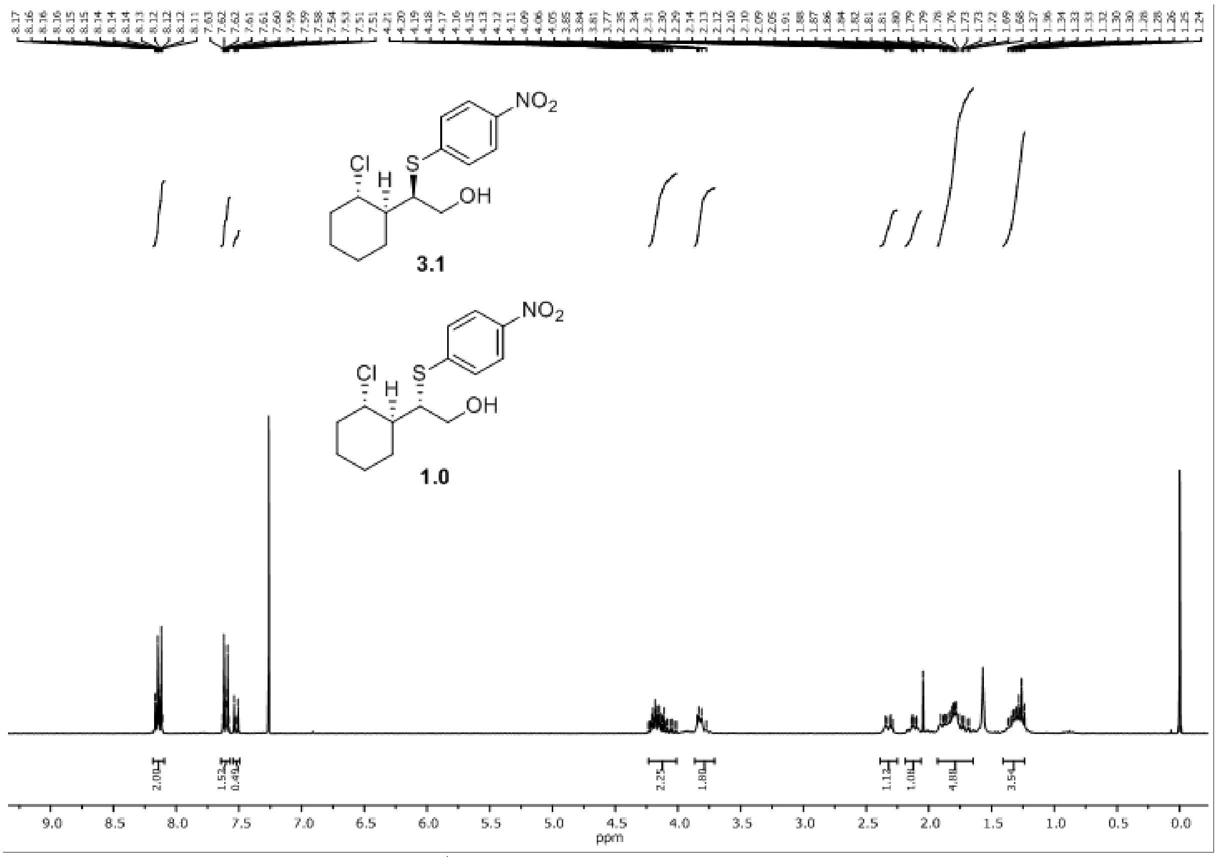

(^{1}H-NMR, 300 MHz, $CDCl_3$)

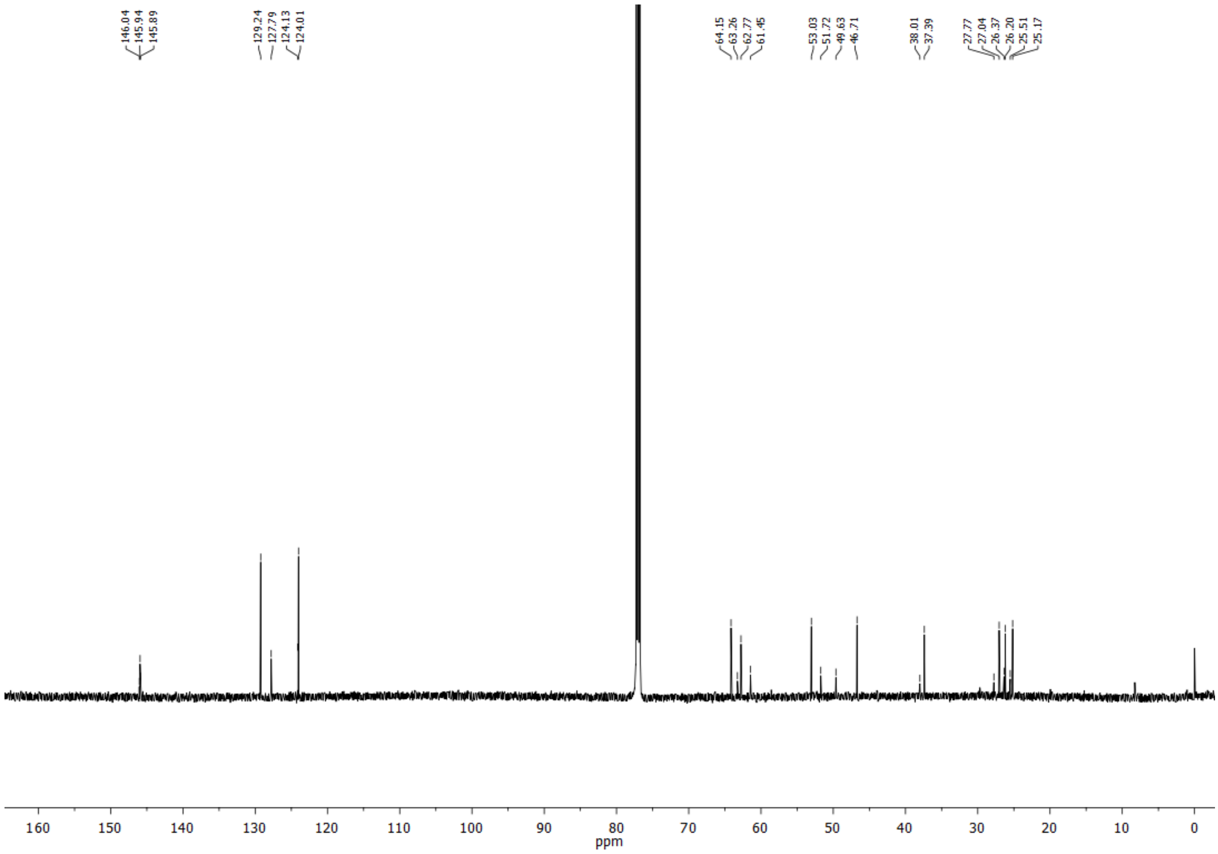

(^{13}C-NMR, 150 MHz, $CDCl_3$)

(*R*)-2-((1*R*,2*S*)-2-Chlorocyclohexyl)-2-(*p*-fluorophenylthio)ethanol (3e)

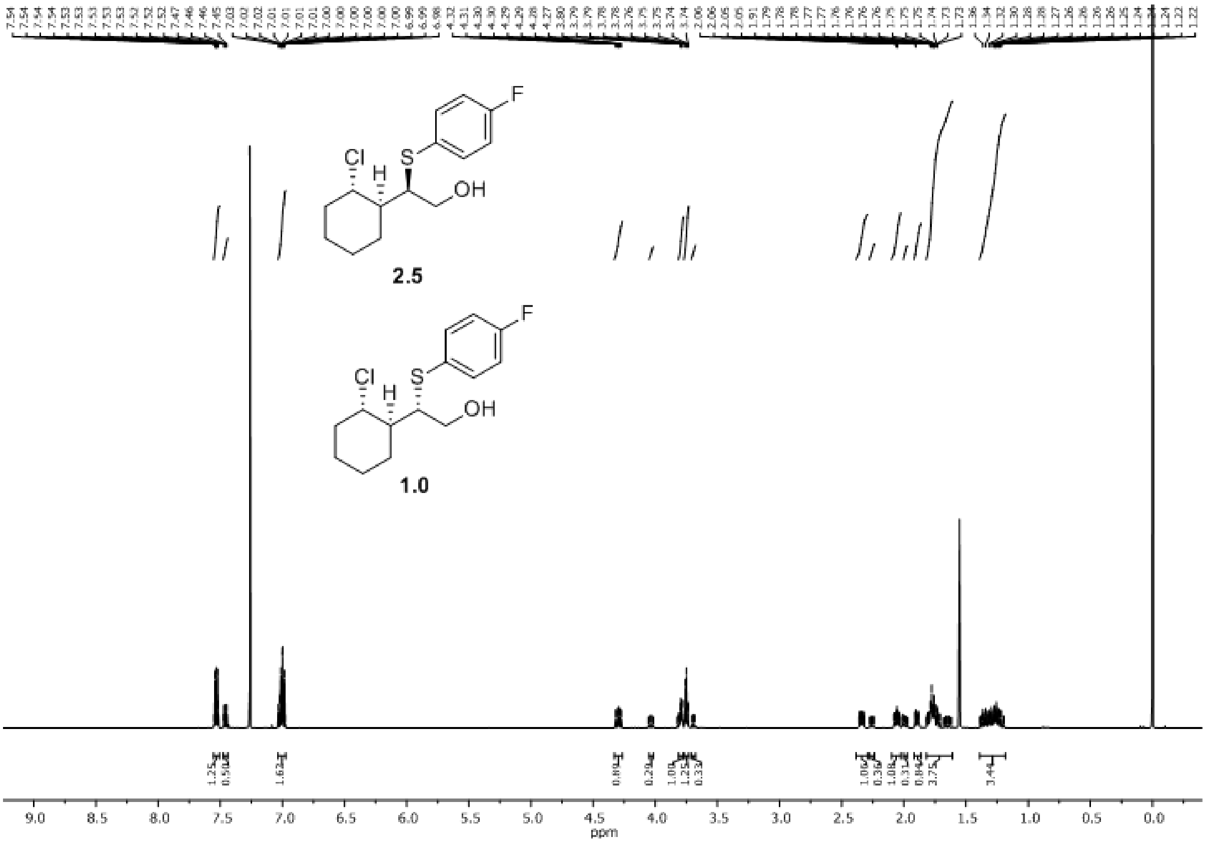

(^{1}H-NMR, 600 MHz, $CDCl_3$)

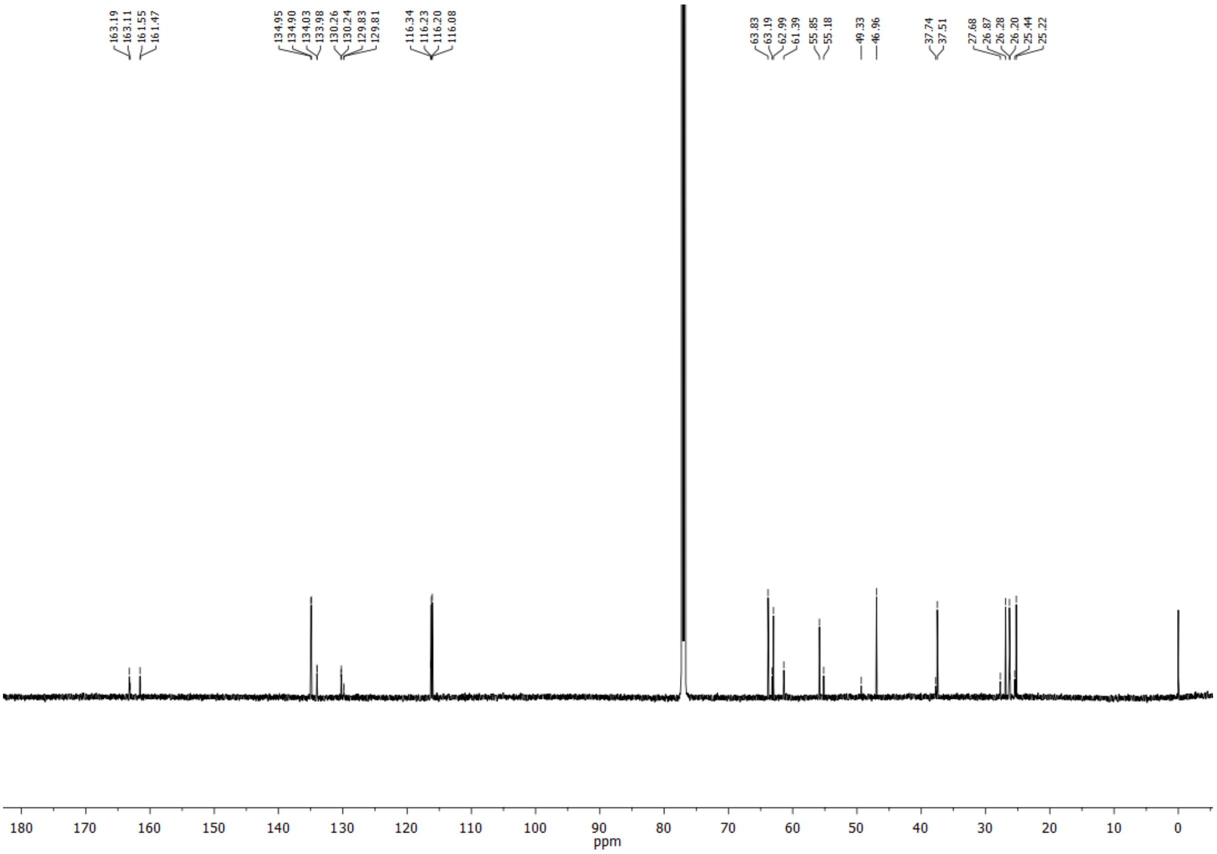

(^{13}C-NMR, 150 MHz, $CDCl_3$)

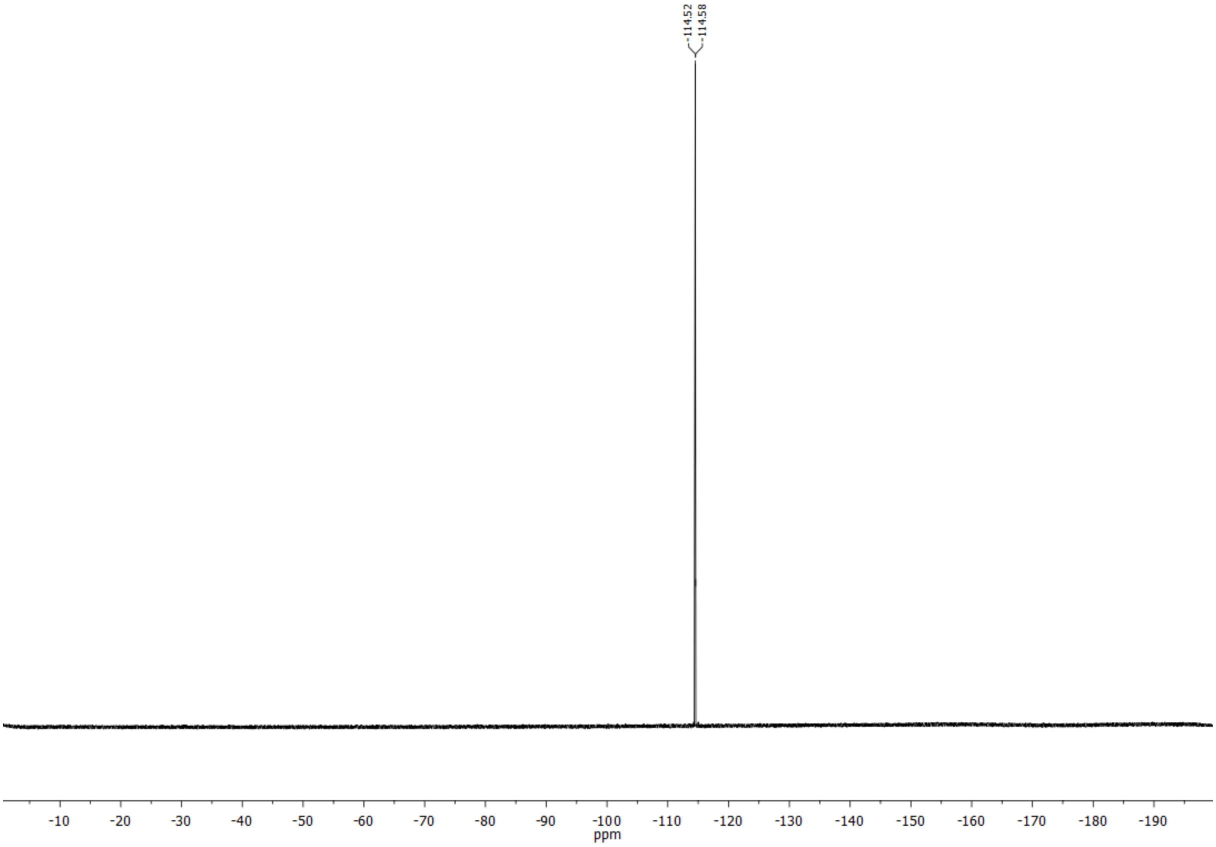

(^{19}F-NMR, 188 MHz, $CDCl_3$)

(*R*)-2-((1*R*,2*S*)-2-Chlorocyclohexyl)-2-(cyclohexylthio)ethanol (3f)

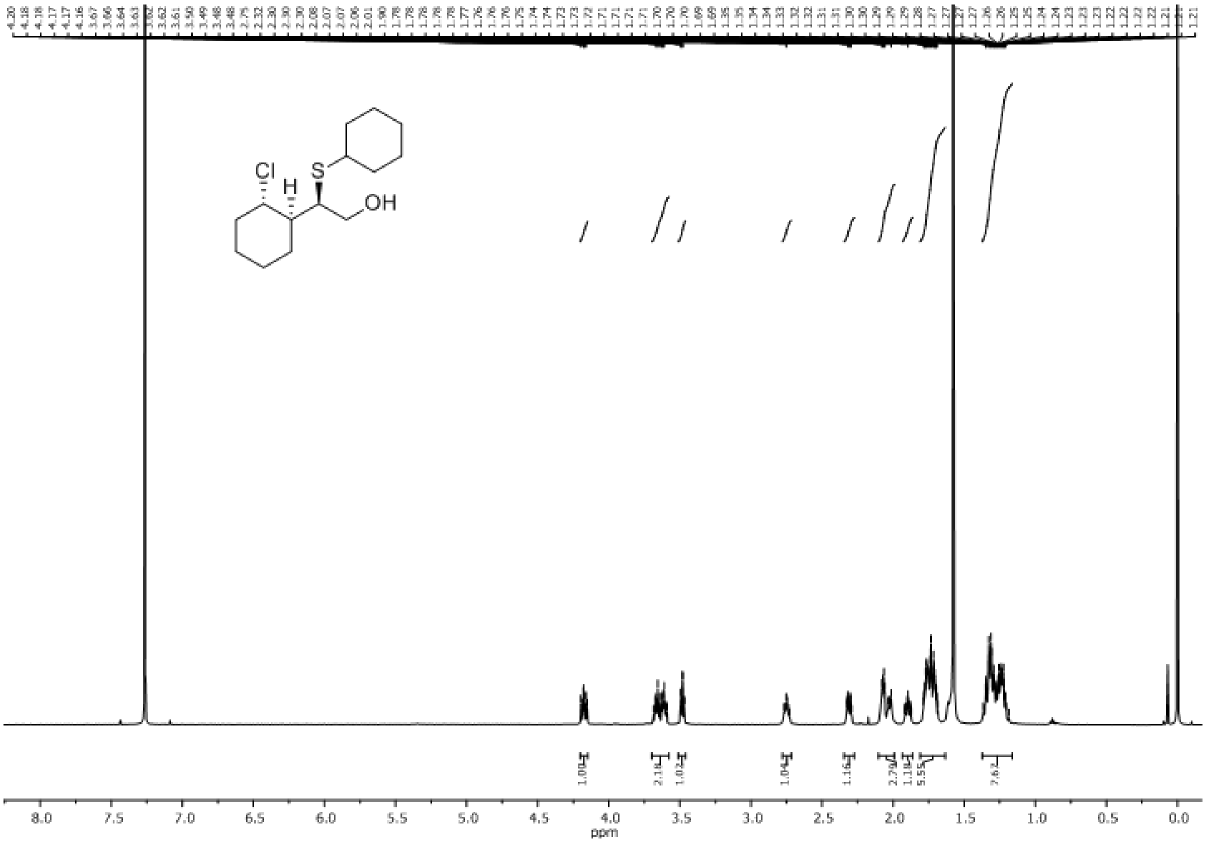

(^{1}H-NMR, 600 MHz, $CDCl_3$)

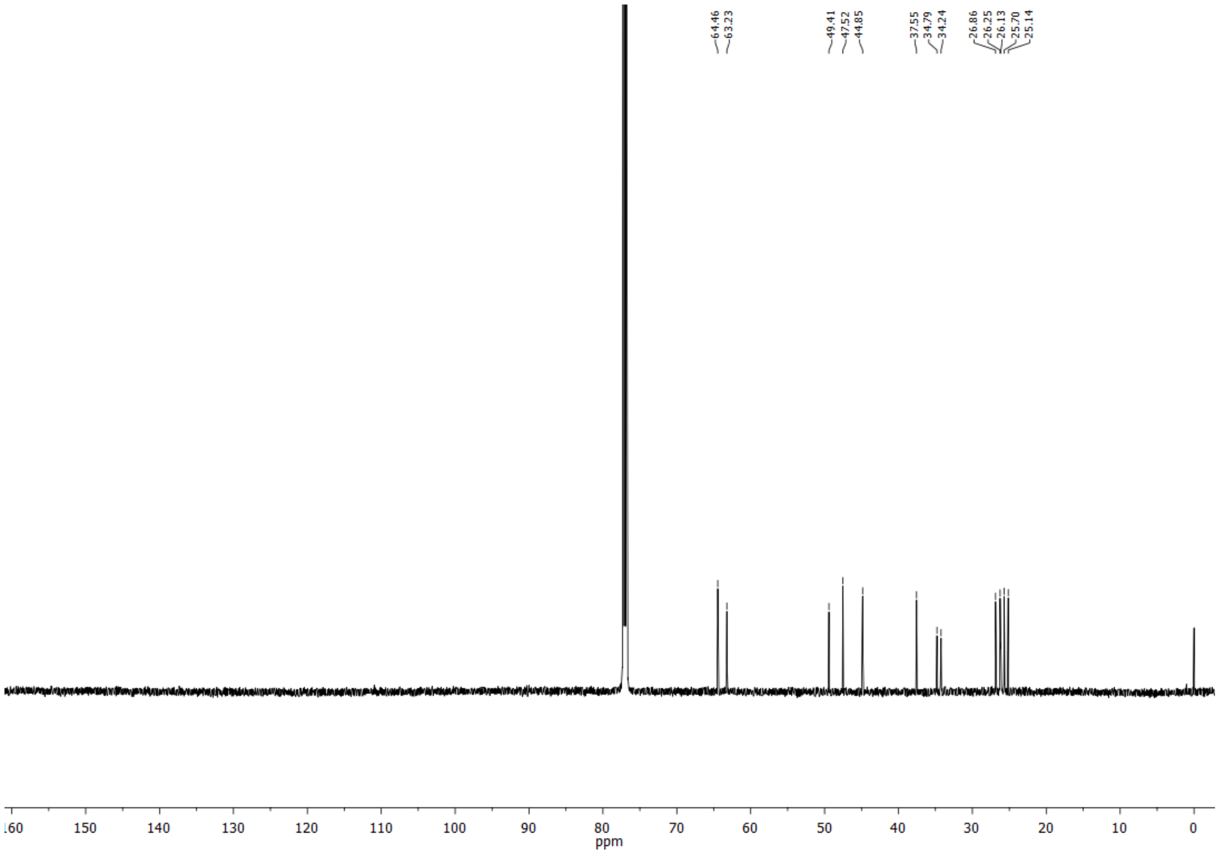

(^{13}C-NMR, 150 MHz, $CDCl_3$)

(*R*)-2-((1*R*,2*S*)-2-Chlorocyclohexyl)-2-(*tert*-butyldisulfanyl)ethanol (3g)

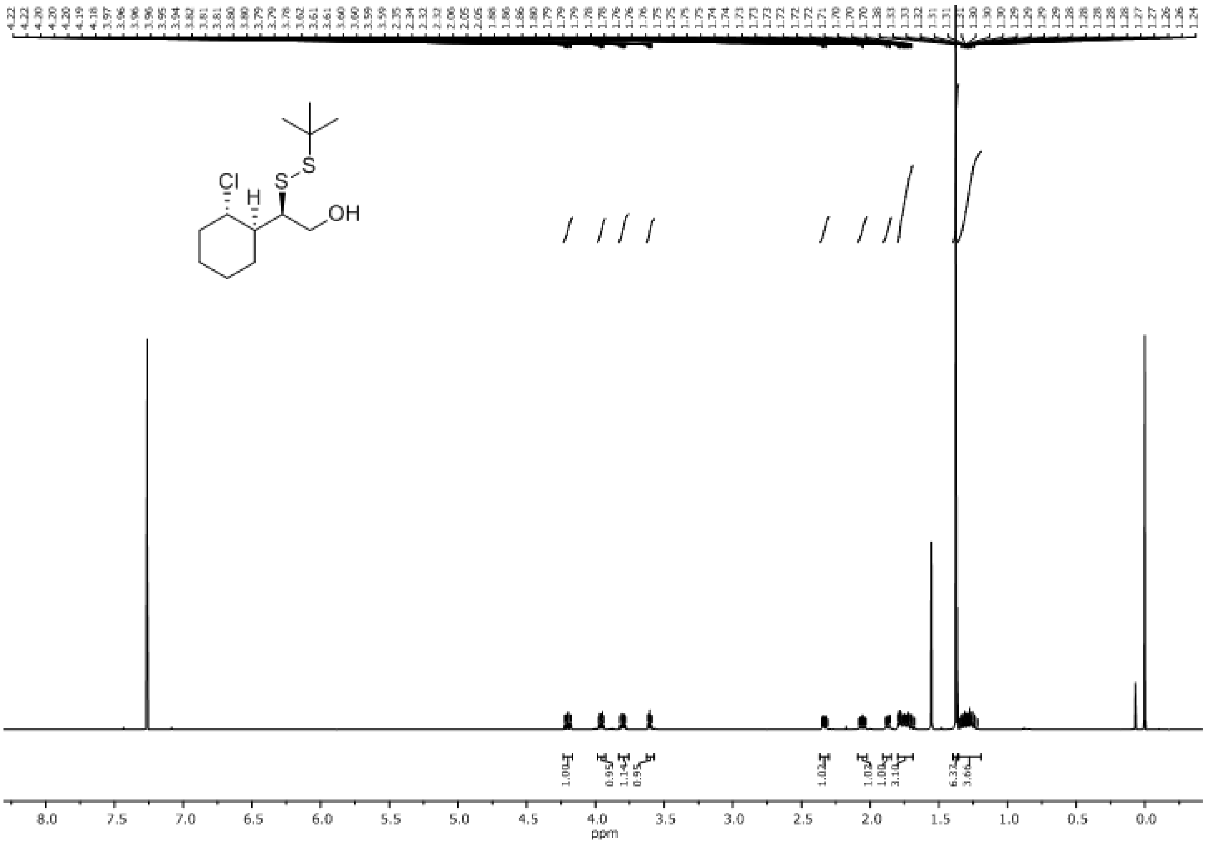

(^{1}H-NMR, 600 MHz, $CDCl_3$)

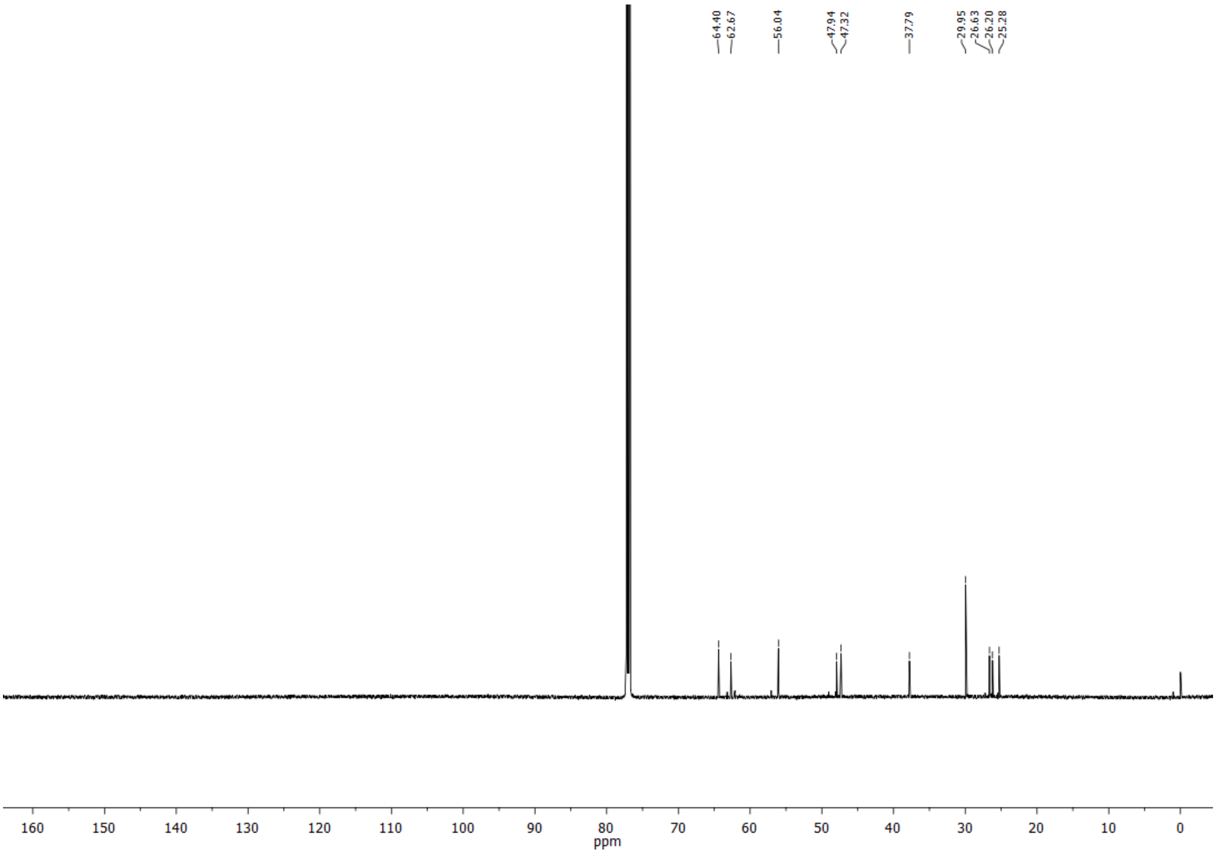

(^{13}C-NMR, 150 MHz, $CDCl_3$)

(*R*)-2-((1*R*,2*S*)-2-Chlorocyclohexyl)-2-(methoxycarbonylthio)ethanol (3h)

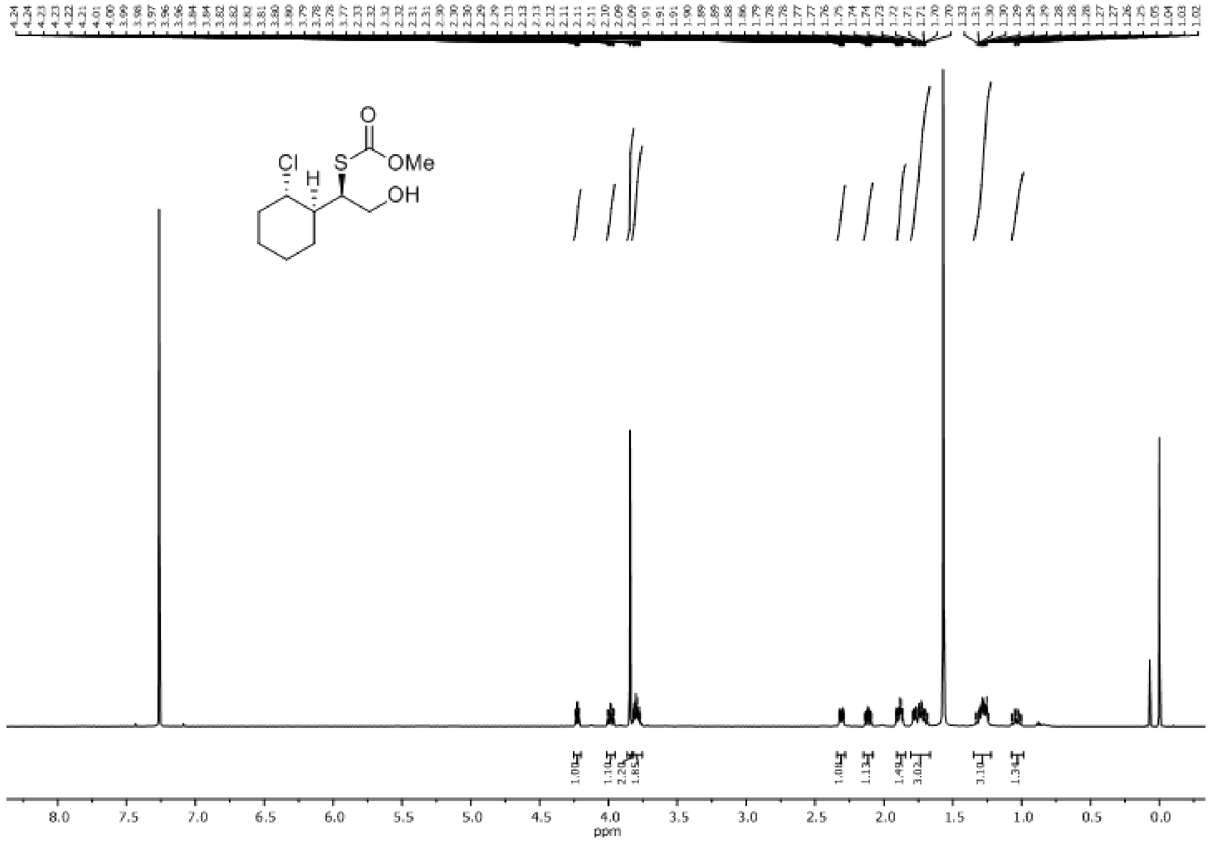

(^{1}H-NMR, 600 MHz, $CDCl_3$)

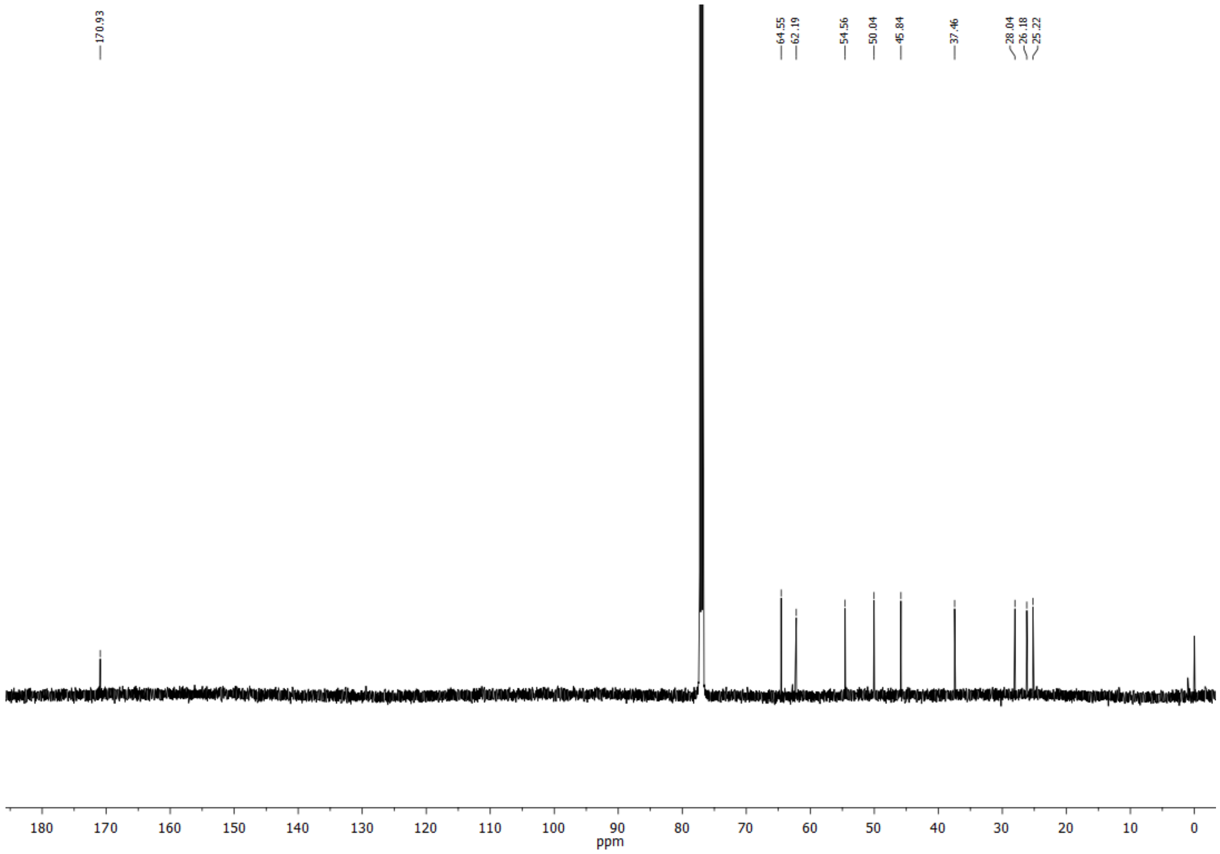

(^{13}C-NMR, 150 MHz, $CDCl_3$)

(*R*)-2-((1*R*,2*S*)-2-Chlorocyclohexyl)-2-(phenylseleno)ethanol (3i)

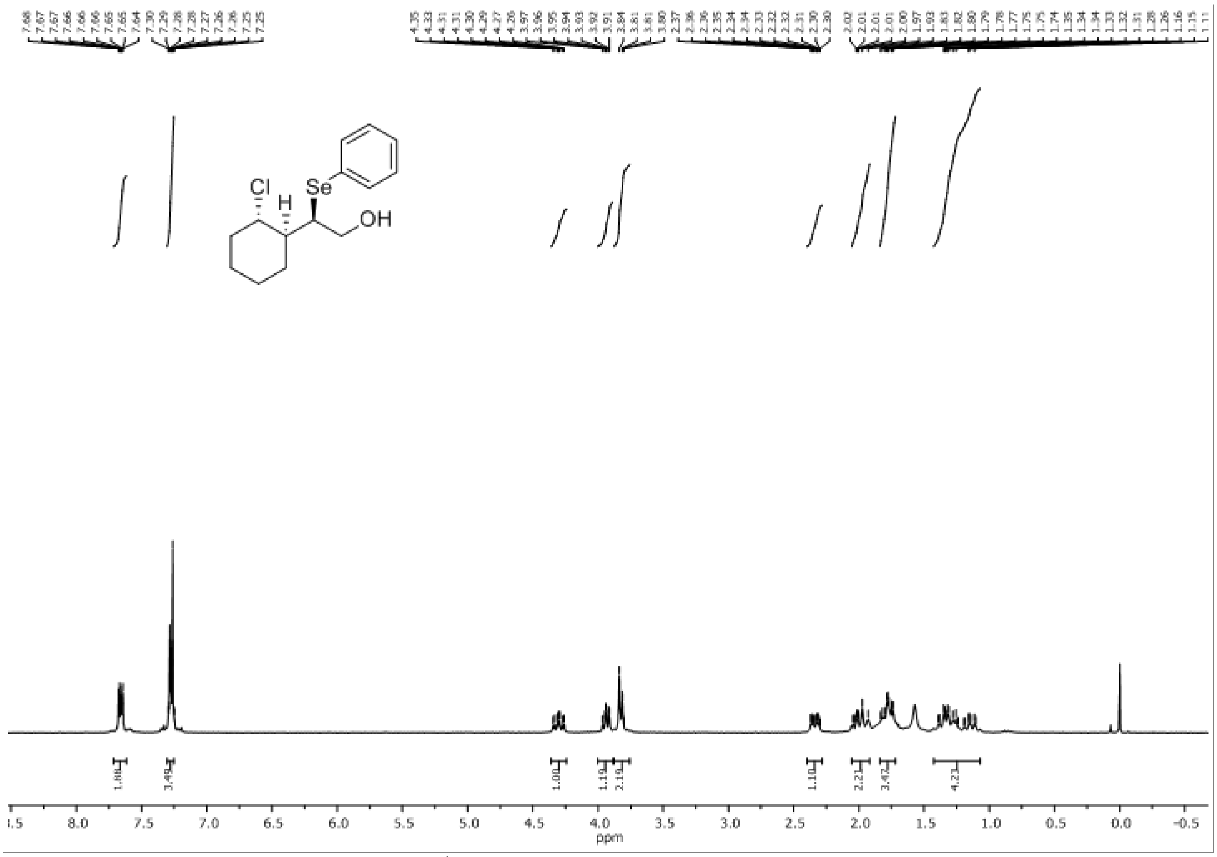

(^{1}H-NMR, 300 MHz, $CDCl_3$)

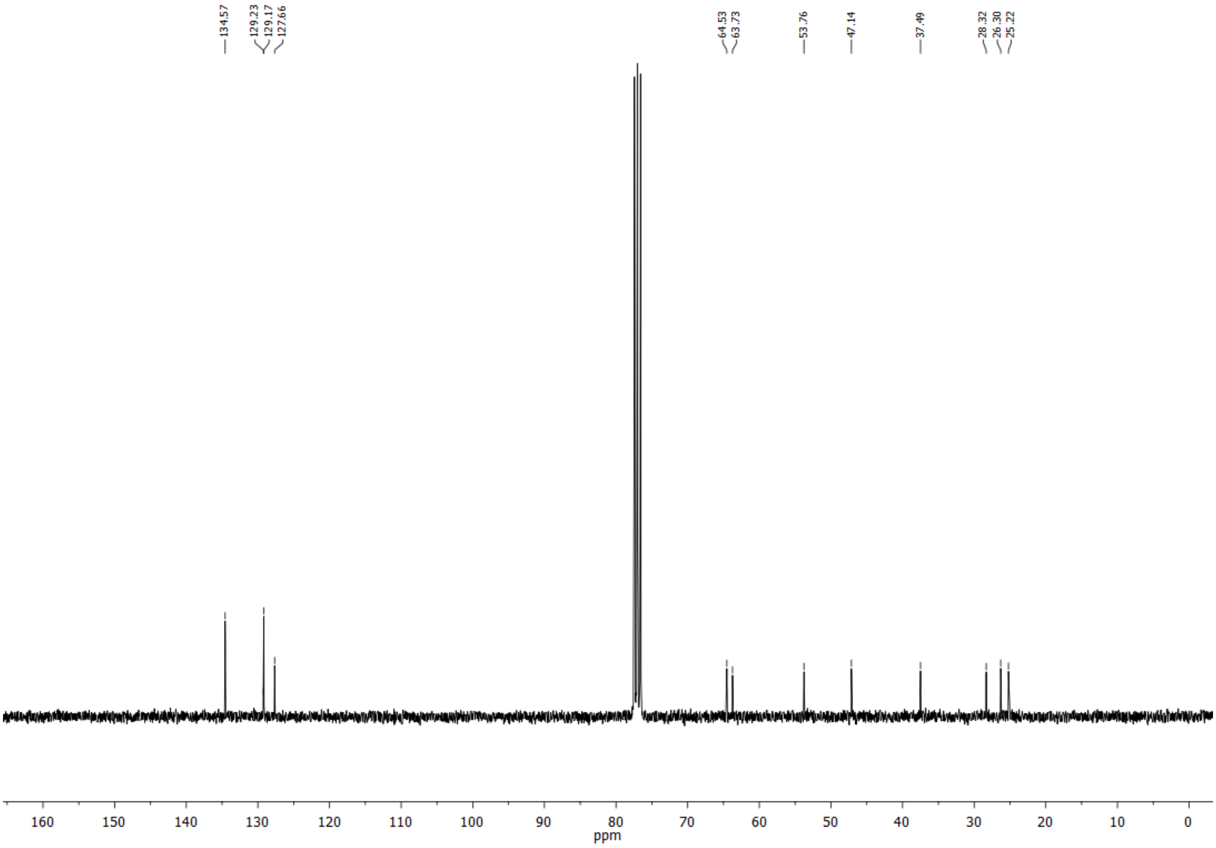

(^{13}C-NMR, 75 MHz, $CDCl_3$)

(2*R*,3*R*,4*S*)-4-Chloro-3-ethyl-2-(*p*-tolylthio)hexan-1-ol (4b)

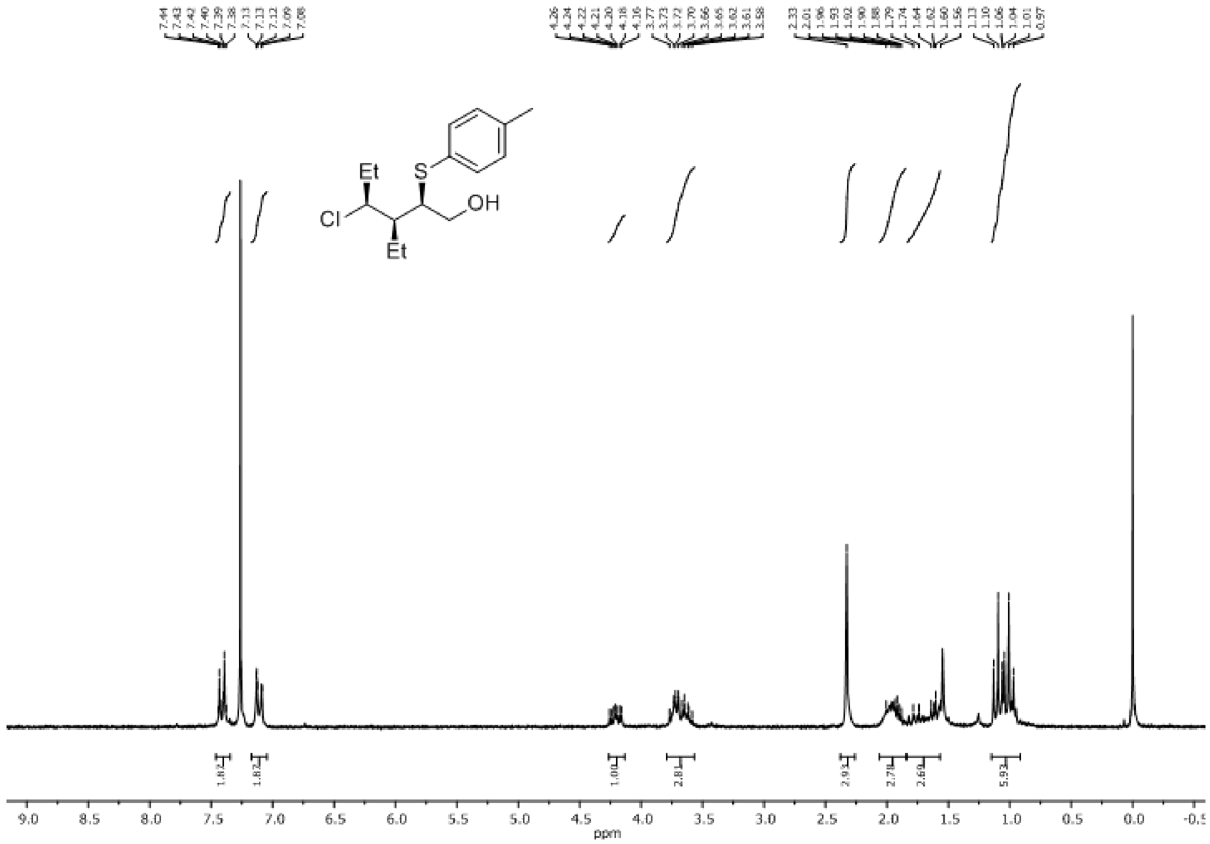

(^{1}H-NMR, 200 MHz, $CDCl_3$)

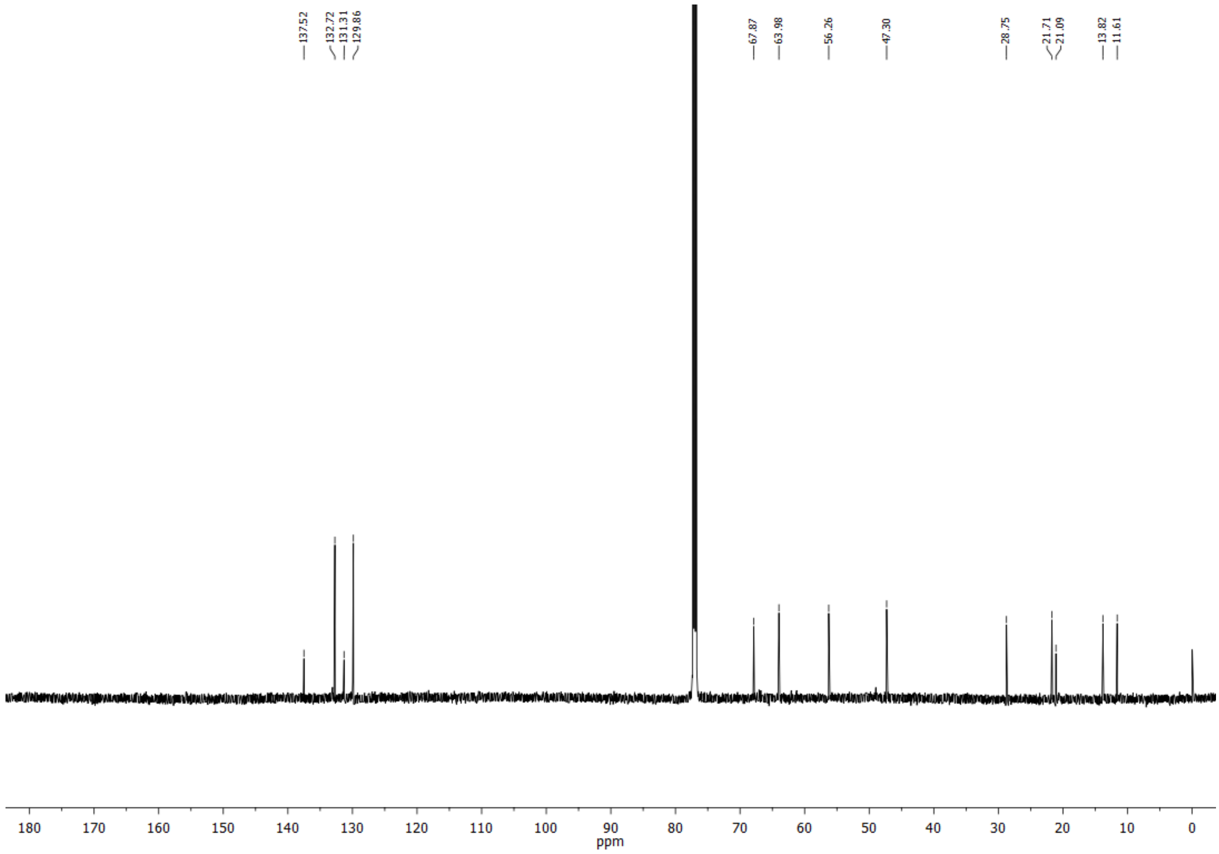

(^{13}C-NMR, 150 MHz, $CDCl_3$)

(2*S*,3*R*,4*S*)-4-Chloro-3-ethyl-2-(*p*-tolylthio)hexan-1-ol (4b')

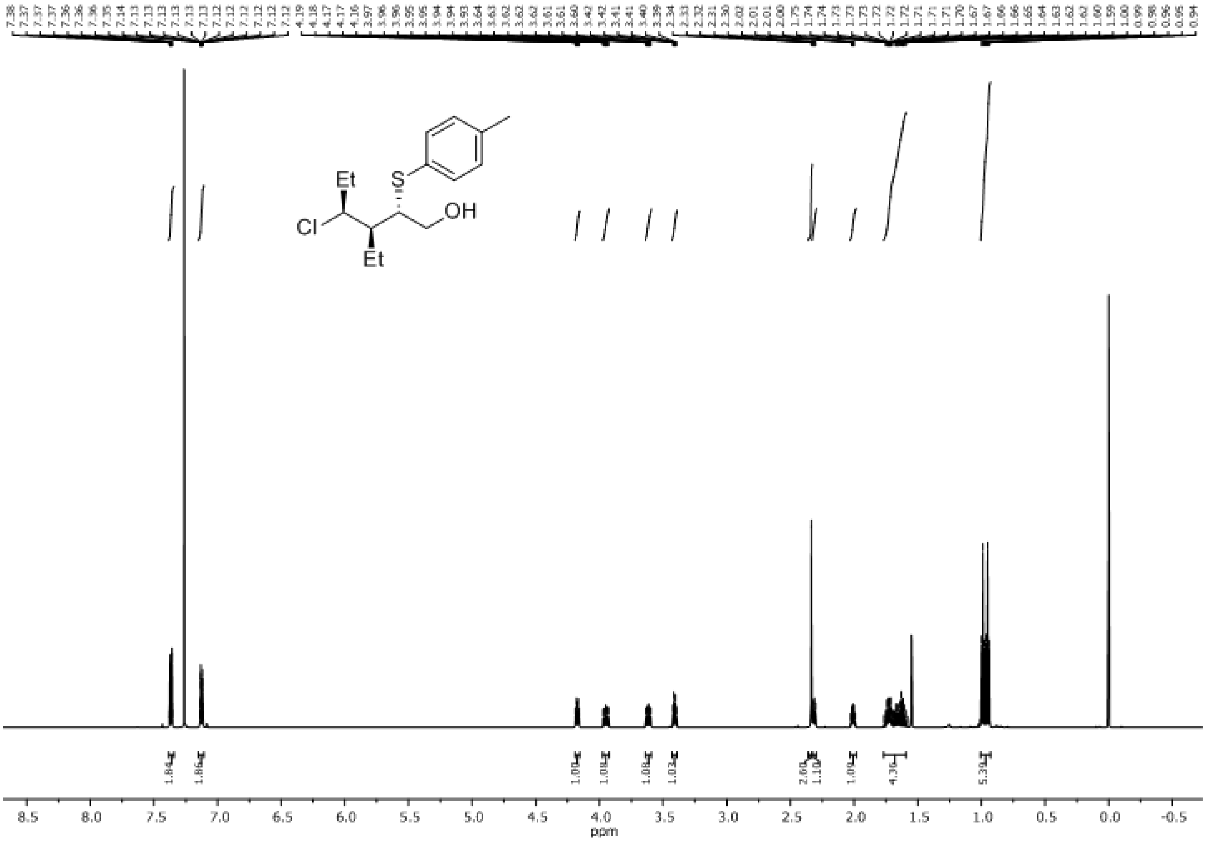

(^{1}H-NMR, 600 MHz, $CDCl_3$)

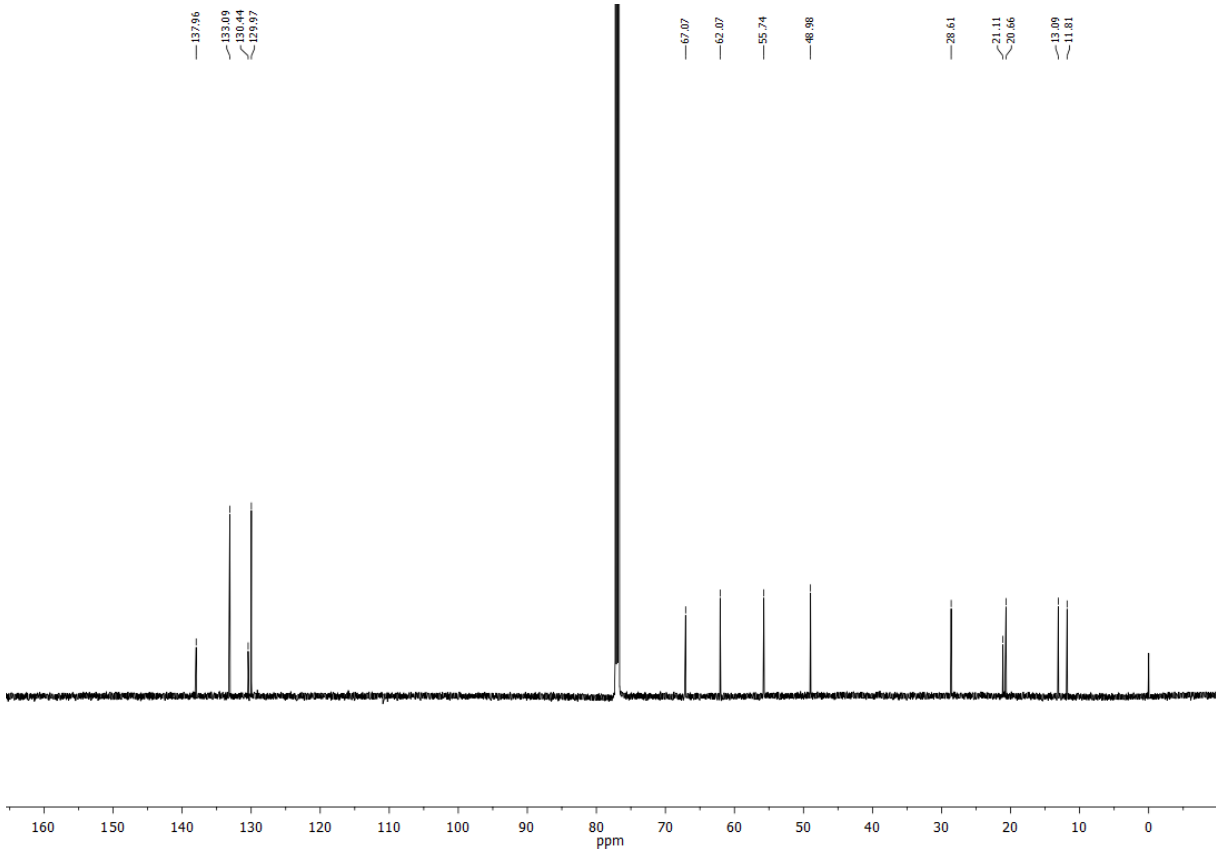

(^{13}C-NMR, 150 MHz, $CDCl_3$)

(2*R*,3*R*,4*R*)-4-Chloro-3,4-diphenyl-2-(*p*-tolylthio)butan-1-ol (4c)

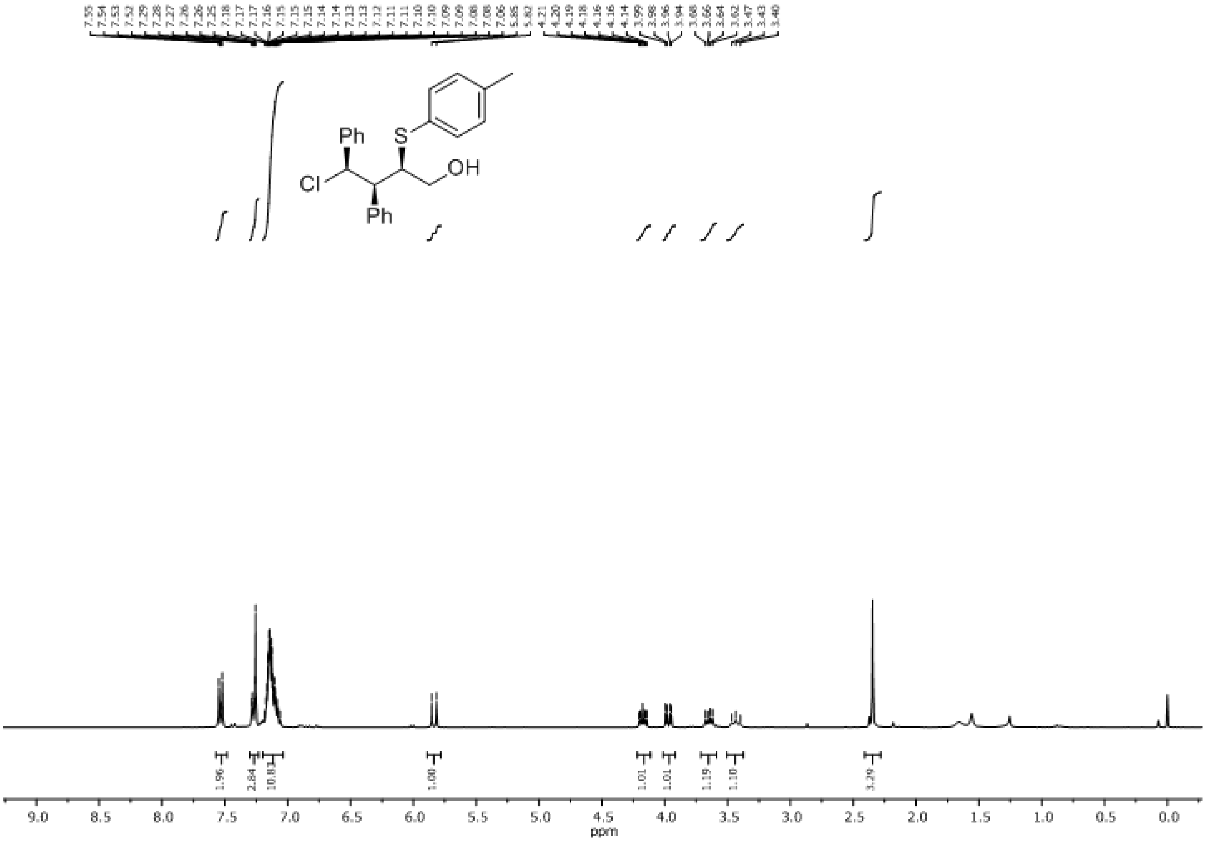

(^{1}H-NMR, 300 MHz, $CDCl_3$)

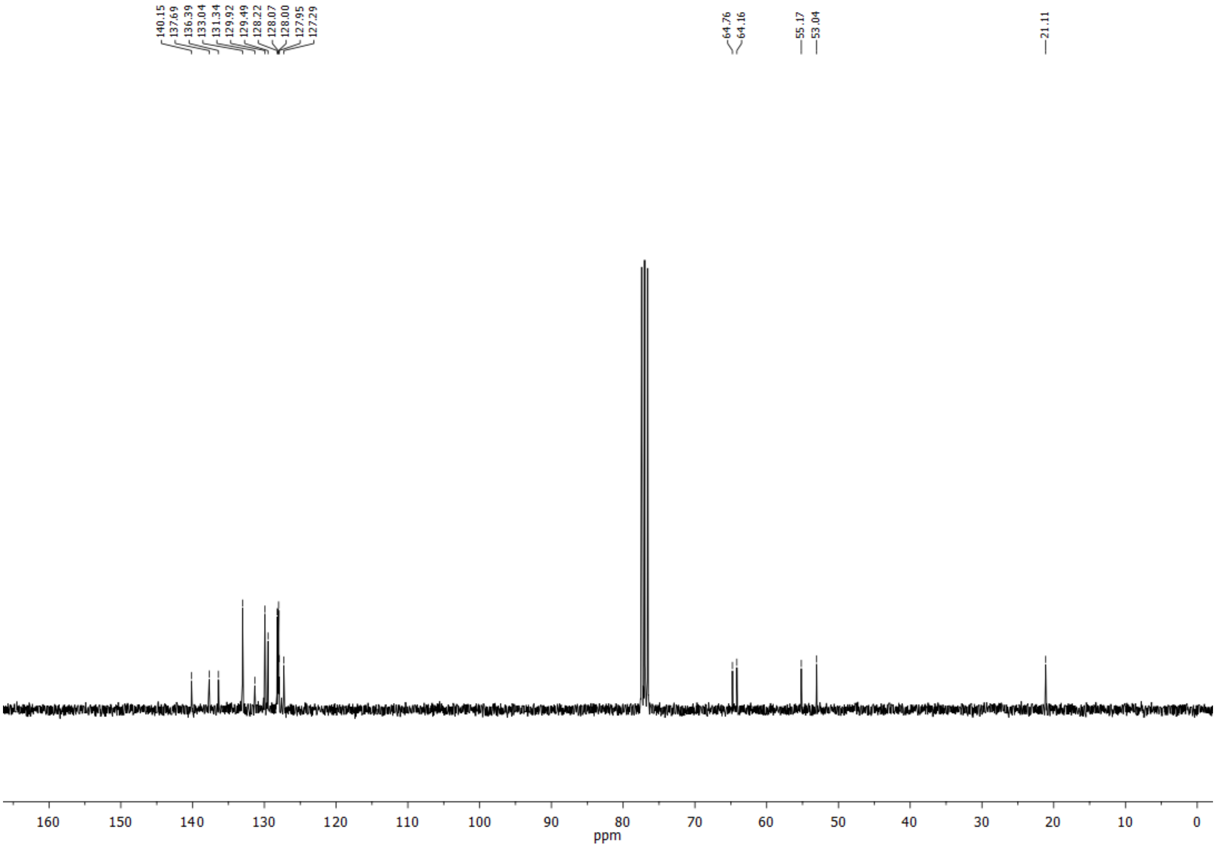

(^{13}C-NMR, 75 MHz, $CDCl_3$)

(*R*)-2-((3*R*,4*R*)-4-Chlorotetrahydrofuran-3-yl)-2-(*p*-tolylthio)ethanol (4d)

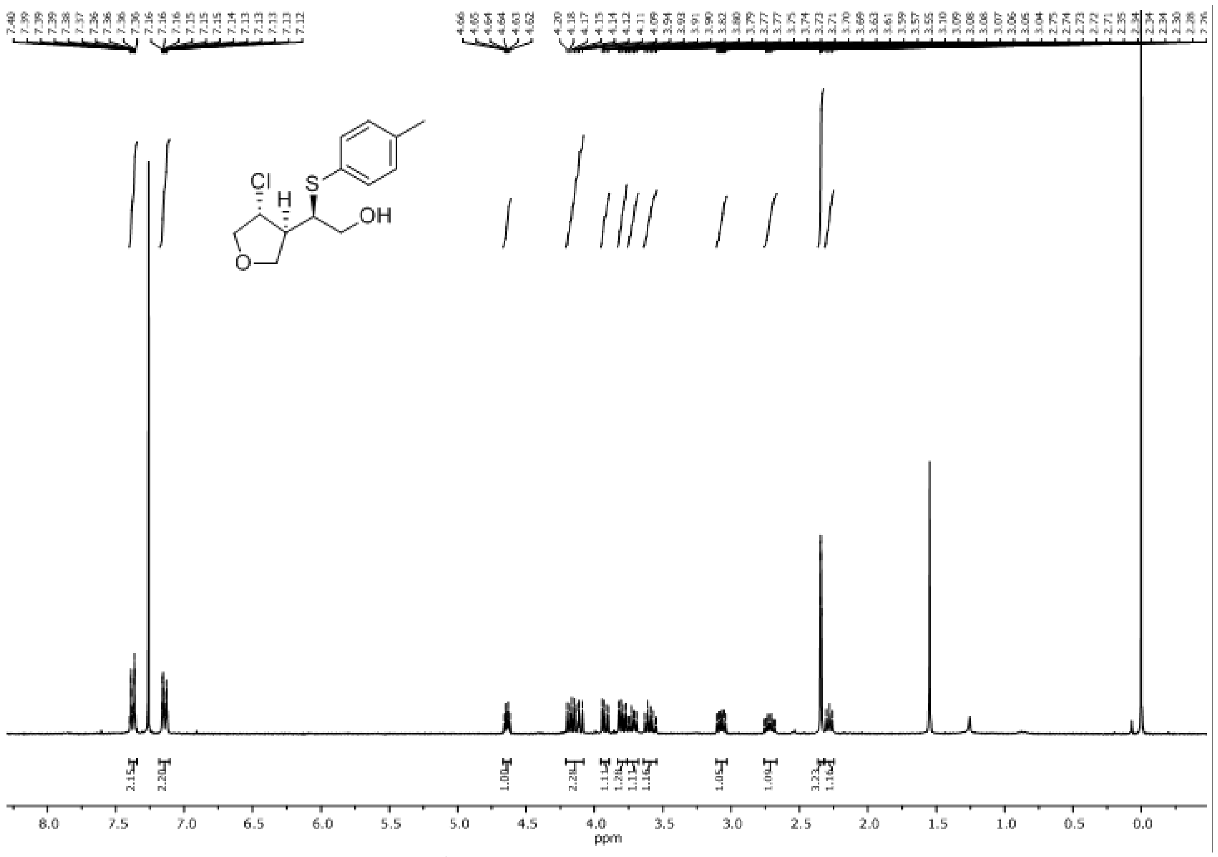

(^{1}H-NMR, 300 MHz, $CDCl_3$)

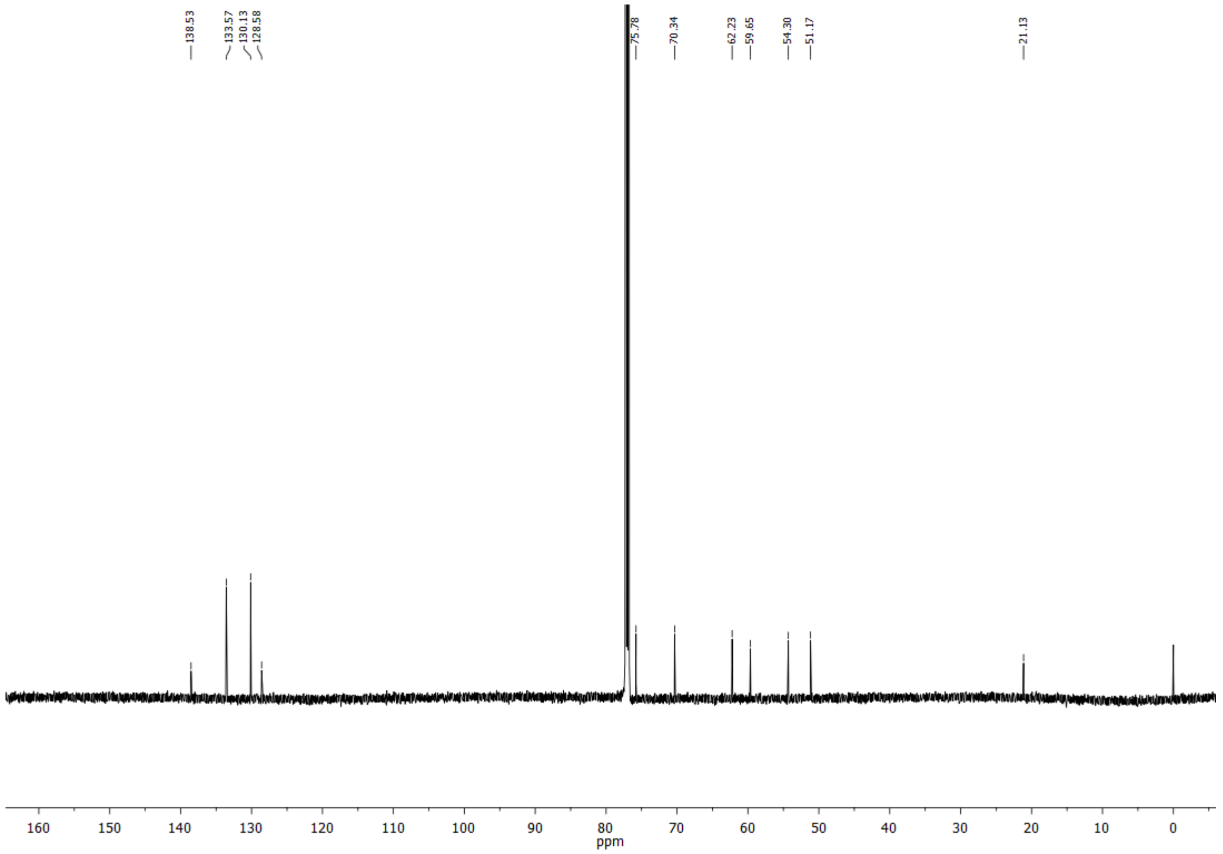

(^{13}C-NMR, 150 MHz, $CDCl_3$)

(*R*)-2-((3*S*,4*R*)-4-Chloro-*N*-Boc-pyrroldin-3-yl)-2-(*p*-tolylthio)ethanol (4e)

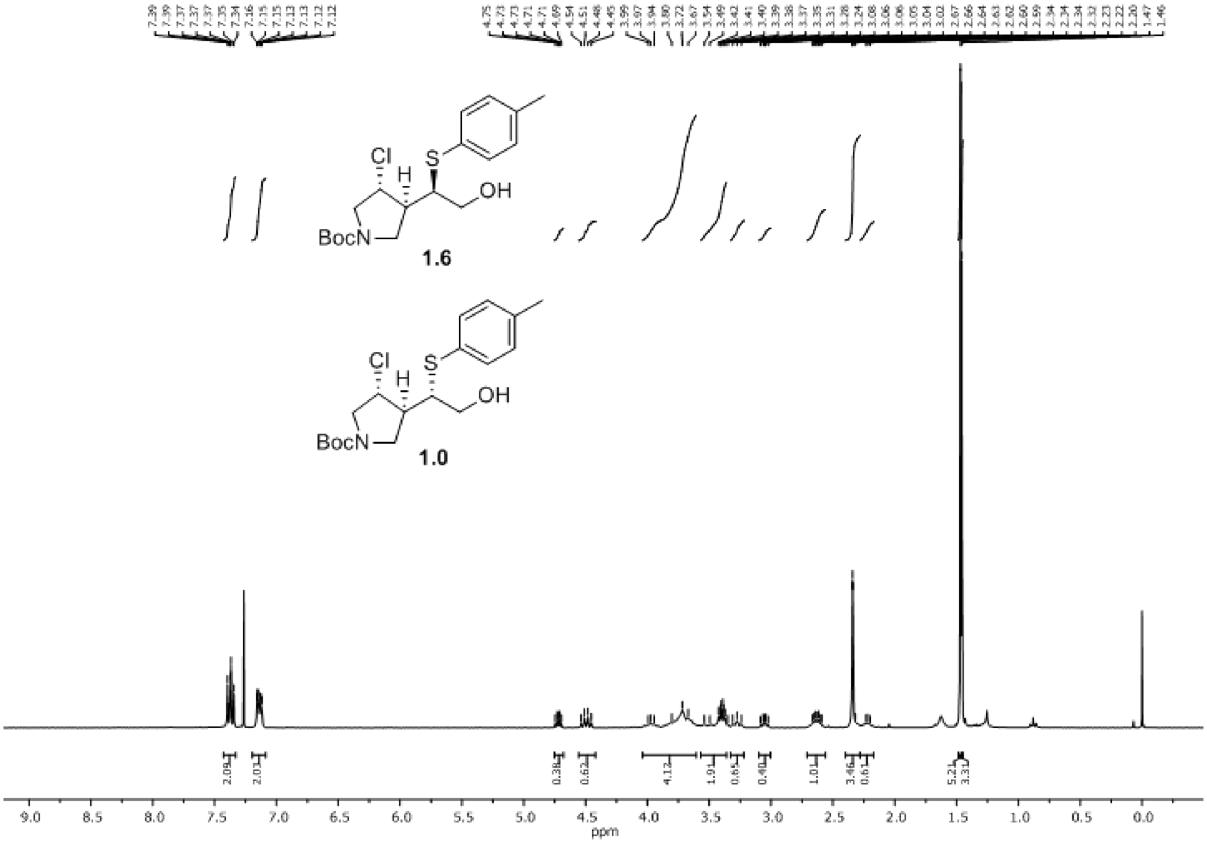

(^{1}H-NMR, 300 MHz, $CDCl_3$)

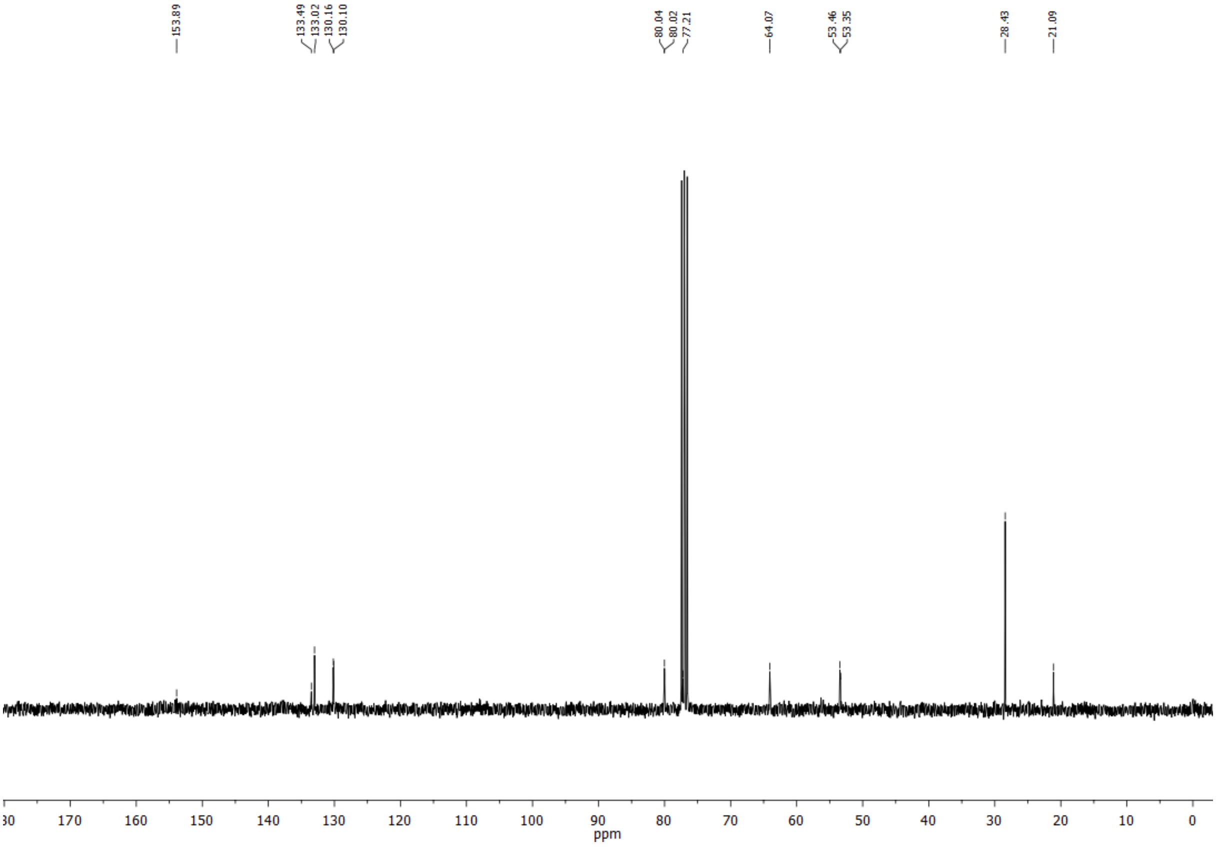

(^{13}C-NMR, 150 MHz, $CDCl_3$)

Ferrocene ester adduct 5

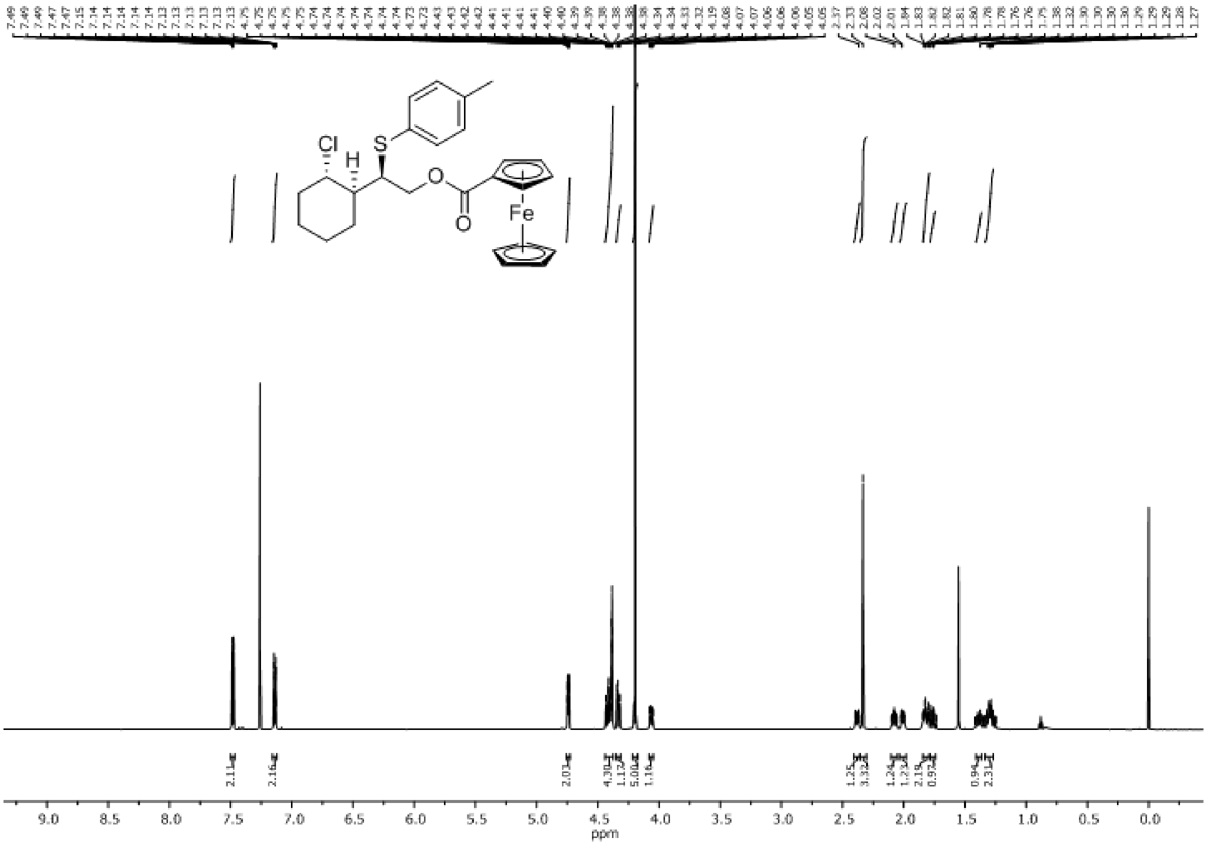

(¹H-NMR, 600 MHz, $CDCl_3$)

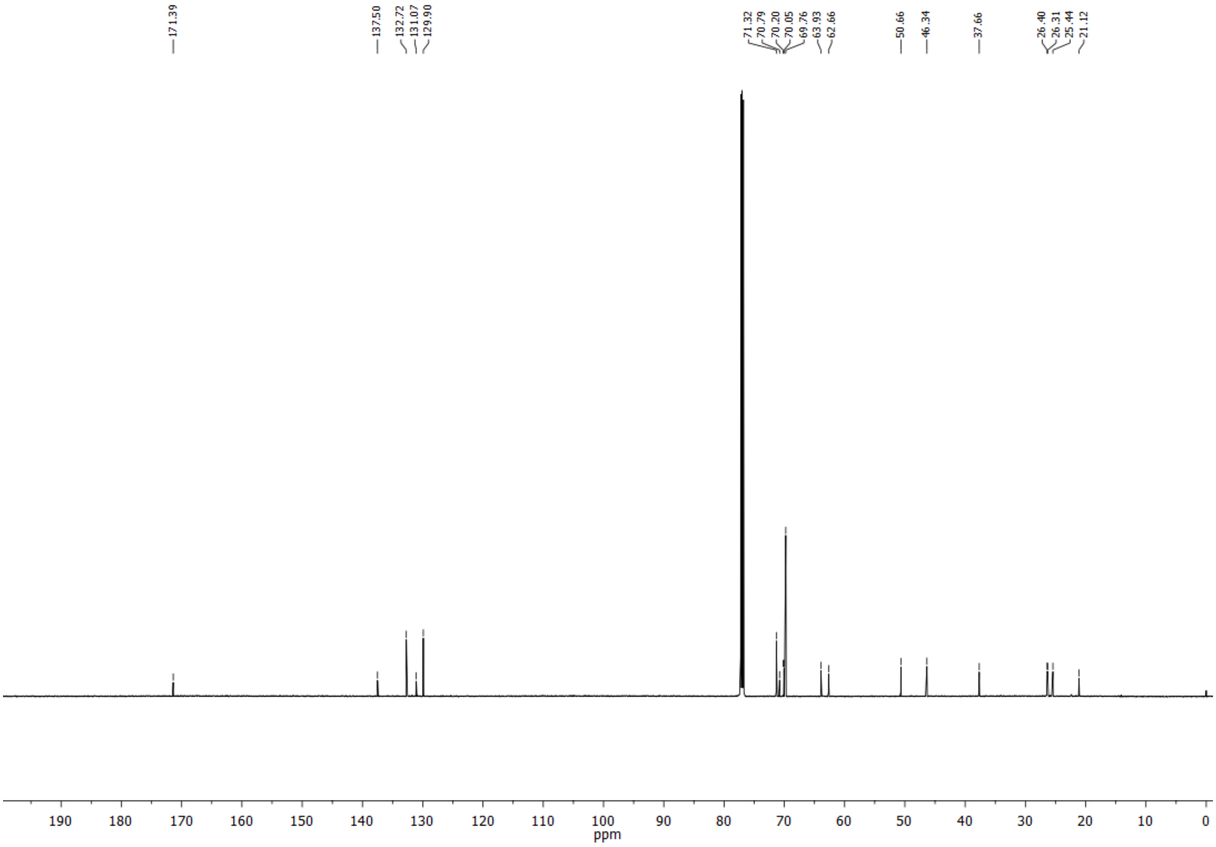

(¹³C-NMR, 150 MHz, $CDCl_3$)

Crystal Structure Determination of 5

Crystals in the form of large orange-brown prisms were obtained by evaporation of a solution of **5** in *n*-pentane. A crystal was cut to appropriate size, then mounted in inert oil on a glass fibre and transferred to the cold gas stream of the diffractometer (Oxford Diffraction Xcalibur E using monochromated Mo *K*α radiation). An absorption correction was implemented on the basis of multi-scans. The structure was refined anisotropically on F^2 using the program SHELXL-97.[1] Hydrogens were refined using rigid methyl groups or a riding model starting from calculated positions. *Special features*: The absolute configuration was confirmed by the Flack parameter, which refined to 0.009(10).

Table S1. Crystallographic data.

Compound	**5**
Formula	$C_{26}H_{29}ClFeO_2S_2$
M_r	496.85
Habit	orange block
Cryst. size (mm)	0.4 × 0.2 × 0.2
Crystal system	orthorhombic
Space group	$P2_12_12_1$
Temperature (°C)	–173
Cell constants:	
a (Å)	7.33306(17)
b (Å)	10.9288(3)
c (Å)	28.4922(8)
α (°)	90
β (°)	90
γ (°)	90
V (Å^3)	2283.41
Z	4
D_x (Mg m^{-3})	1.445
λ (Å)	0.71073
μ (mm^{-1})	0.89
Transmissions	0.936 – 1.000
F(000)	1040
$2\theta_{max}$	62
Refl. measured	61222
Refl. indep.	6884
R_{int}	0.039
Parameters	281
Restraints	0
$wR(F^2$, all refl.)	0.066
$R(F, >4\sigma(F))$	0.030
S	1.18
max. Δρ (e Å^{-3})	0.38

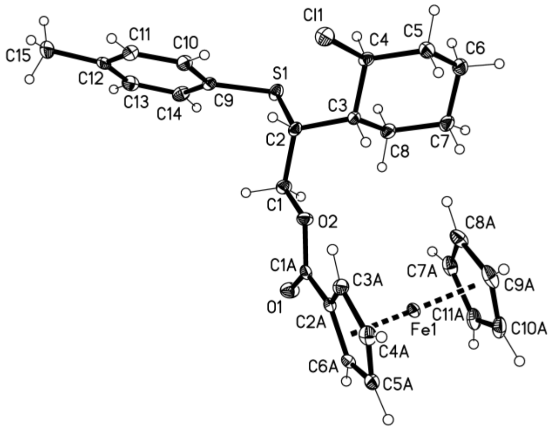

Table S2. Atomic coordinates (x 10^4) and equivalent isotropic displacement parameters ($Å^2$x 10^3). U(eq) is defined as one third of the trace of the orthogonalized U_{ij} tensor.

	x	y	z	U(eq)
C(1)	4605(2)	2629.9(15)	5779.0(6)	15.3(3)
C(2)	4673(2)	3318.6(15)	6245.4(5)	13.2(3)
S(1)	3764.6(6)	2316.1(4)	6704.8(1)	16.5(1)
C(3)	3598(2)	4525.2(15)	6237.1(5)	12.6(3)
C(4)	3832(2)	5301.0(14)	6680.0(5)	13.3(3)
Cl(1)	6234.4(5)	5598.6(4)	6795.5(1)	19.7(1)
C(5)	2865(2)	6529.4(16)	6639.6(6)	17.5(3)
C(6)	824(2)	6340.9(17)	6548.3(6)	18.7(3)
C(7)	513(2)	5536.5(18)	6119.3(6)	18.7(3)
C(8)	1534(2)	4322.4(17)	6158.1(6)	16.6(3)
C(9)	5652(2)	1380.9(15)	6857.4(5)	14.1(3)
C(10)	7359(2)	1863.0(16)	6970.1(6)	16.2(3)
C(11)	8739(2)	1096.8(16)	7122.8(6)	17.7(3)
C(12)	8450(3)	-157.9(16)	7176.0(6)	17.0(3)
C(13)	6749(3)	-626.7(17)	7063.6(6)	19.7(3)
C(14)	5362(3)	127.7(16)	6899.2(6)	18.4(3)
C(15)	9939(3)	-970.3(18)	7368.4(7)	24.7(4)
O(1)	4306.1(19)	2110.3(11)	4870.7(4)	20.5(3)
O(2)	5157.2(16)	3489.4(11)	5417.5(4)	14.7(2)
C(1A)	4911(2)	3105.1(15)	4971.8(6)	13.4(3)
Fe(1)	4059.8(3)	5575.2(2)	4470.2(1)	10.9(1)
C(2A)	5487(2)	4038.5(14)	4630.7(5)	11.7(3)
C(3A)	6563(2)	5103.5(16)	4725.3(6)	14.1(3)
C(4A)	6758(2)	5749.3(16)	4294.4(6)	16.5(3)
C(5A)	5809(2)	5092.5(15)	3936.4(6)	16.9(3)
C(6A)	5017(2)	4035.5(15)	4142.5(6)	14.6(3)
C(7A)	1651(2)	5510.7(17)	4829.7(6)	18.4(3)
C(8A)	2791(2)	6471.8(16)	5003.7(6)	19.0(3)
C(9A)	3142(2)	7290.2(16)	4627.0(7)	19.9(4)
C(10A)	2236(2)	6845.3(17)	4222.7(7)	20.1(4)
C(11A)	1315(2)	5744.0(17)	4344.9(6)	19.1(3)

Table 3. Selected bond lengths [Å] and angles [°].

C(1)-O(2)	1.4514(19)	C(1A)-C(2A)	1.471(2)
C(1)-C(2)	1.528(2)	Fe(1)-C(2A)	2.0309(15)
C(2)-C(3)	1.537(2)	Fe(1)-C(8A)	2.0337(17)
C(2)-S(1)	1.8324(16)	Fe(1)-C(3A)	2.0403(16)
S(1)-C(9)	1.7748(17)	Fe(1)-C(9A)	2.0411(18)
C(3)-C(4)	1.530(2)	Fe(1)-C(7A)	2.0433(16)
C(3)-C(8)	1.546(2)	Fe(1)-C(6A)	2.0484(16)
C(4)-C(5)	1.523(2)	Fe(1)-C(4A)	2.0496(16)
C(4)-Cl(1)	1.8213(16)	Fe(1)-C(10A)	2.0527(17)
C(5)-C(6)	1.533(3)	Fe(1)-C(11A)	2.0527(16)
C(6)-C(7)	1.523(2)	Fe(1)-C(5A)	2.0582(17)
C(7)-C(8)	1.528(2)	C(2A)-C(3A)	1.432(2)
C(9)-C(14)	1.391(2)	C(2A)-C(6A)	1.433(2)
C(9)-C(10)	1.395(2)	C(3A)-C(4A)	1.423(2)
C(10)-C(11)	1.384(2)	C(4A)-C(5A)	1.428(2)
C(11)-C(12)	1.396(2)	C(5A)-C(6A)	1.420(2)
C(12)-C(13)	1.386(3)	C(7A)-C(11A)	1.426(3)
C(12)-C(15)	1.510(2)	C(7A)-C(8A)	1.431(3)
C(13)-C(14)	1.391(2)	C(8A)-C(9A)	1.420(3)
O(1)-C(1A)	1.209(2)	C(9A)-C(10A)	1.416(3)
O(2)-C(1A)	1.3496(19)	C(10A)-C(11A)	1.423(3)
O(2)-C(1)-C(2)	106.81(13)	C(10)-C(11)-C(12)	121.15(17)
C(1)-C(2)-C(3)	113.13(13)	C(13)-C(12)-C(11)	118.36(17)
C(1)-C(2)-S(1)	108.36(11)	C(13)-C(12)-C(15)	121.14(17)
C(3)-C(2)-S(1)	109.74(11)	C(11)-C(12)-C(15)	120.46(18)
C(9)-S(1)-C(2)	103.64(8)	C(12)-C(13)-C(14)	121.12(17)
C(4)-C(3)-C(2)	113.91(12)	C(13)-C(14)-C(9)	120.05(17)
C(4)-C(3)-C(8)	108.03(13)	C(1A)-O(2)-C(1)	115.40(13)
C(2)-C(3)-C(8)	112.42(14)	O(1)-C(1A)-O(2)	123.58(15)
C(5)-C(4)-C(3)	111.96(13)	O(1)-C(1A)-C(2A)	124.85(15)
C(5)-C(4)-Cl(1)	107.85(11)	O(2)-C(1A)-C(2A)	111.57(14)
C(3)-C(4)-Cl(1)	110.89(11)	C(4A)-C(3A)-C(2A)	107.22(14)
C(4)-C(5)-C(6)	110.42(14)	C(3A)-C(4A)-C(5A)	108.54(15)
C(7)-C(6)-C(5)	111.11(14)	C(6A)-C(5A)-C(4A)	108.22(14)
C(6)-C(7)-C(8)	111.71(14)	C(5A)-C(6A)-C(2A)	107.54(14)
C(7)-C(8)-C(3)	111.47(15)	C(11A)-C(7A)-C(8A)	107.77(16)
C(14)-C(9)-C(10)	119.29(16)	C(9A)-C(8A)-C(7A)	107.83(16)
C(14)-C(9)-S(1)	117.95(13)	C(10A)-C(9A)-C(8A)	108.28(16)
C(10)-C(9)-S(1)	122.58(13)	C(9A)-C(10A)-C(11A)	108.29(16)
C(11)-C(10)-C(9)	119.99(16)	C(10A)-C(11A)-C(7A)	107.83(16)

Table 4. Selected torsion angles [°].

O(2)-C(1)-C(2)-C(3)	52.43(17)
O(2)-C(1)-C(2)-S(1)	174.34(10)
C(1)-C(2)-S(1)-C(9)	82.54(12)
C(3)-C(2)-S(1)-C(9)	-153.49(11)
C(1)-C(2)-C(3)-C(4)	-173.15(13)
S(1)-C(2)-C(3)-C(4)	65.71(15)
C(1)-C(2)-C(3)-C(8)	63.54(17)
S(1)-C(2)-C(3)-C(8)	-57.60(15)
C(2)-C(3)-C(4)-C(5)	175.48(13)
C(8)-C(3)-C(4)-C(5)	-58.86(17)
C(2)-C(3)-C(4)-Cl(1)	54.97(16)
C(8)-C(3)-C(4)-Cl(1)	-179.37(11)
C(3)-C(4)-C(5)-C(6)	58.46(18)
Cl(1)-C(4)-C(5)-C(6)	-179.27(11)
C(4)-C(5)-C(6)-C(7)	-54.78(19)
C(5)-C(6)-C(7)-C(8)	54.16(19)
C(6)-C(7)-C(8)-C(3)	-56.14(18)
C(4)-C(3)-C(8)-C(7)	57.16(17)
C(2)-C(3)-C(8)-C(7)	-176.30(13)
C(2)-S(1)-C(9)-C(14)	-133.86(14)
C(2)-S(1)-C(9)-C(10)	51.09(15)
C(14)-C(9)-C(10)-C(11)	-0.2(3)
S(1)-C(9)-C(10)-C(11)	174.81(13)
C(9)-C(10)-C(11)-C(12)	-1.3(3)
C(10)-C(11)-C(12)-C(13)	1.2(3)
C(10)-C(11)-C(12)-C(15)	-176.61(16)
C(11)-C(12)-C(13)-C(14)	0.4(3)
C(15)-C(12)-C(13)-C(14)	178.14(16)
C(12)-C(13)-C(14)-C(9)	-1.8(3)
C(10)-C(9)-C(14)-C(13)	1.7(3)
S(1)-C(9)-C(14)-C(13)	-173.52(13)
C(2)-C(1)-O(2)-C(1A)	-170.09(13)
C(1)-O(2)-C(1A)-O(1)	-1.3(2)
C(1)-O(2)-C(1A)-C(2A)	179.33(13)
O(1)-C(1A)-C(2A)-C(3A)	-165.36(17)
O(2)-C(1A)-C(2A)-C(3A)	14.0(2)
O(1)-C(1A)-C(2A)-C(6A)	16.0(3)
O(2)-C(1A)-C(2A)-C(6A)	-164.64(14)
C(6A)-C(2A)-C(3A)-C(4A)	-0.25(18)
C(1A)-C(2A)-C(3A)-C(4A)	-179.11(15)
C(2A)-C(3A)-C(4A)-C(5A)	0.12(18)
C(3A)-C(4A)-C(5A)-C(6A)	0.05(19)
C(4A)-C(5A)-C(6A)-C(2A)	-0.20(18)
C(3A)-C(2A)-C(6A)-C(5A)	0.28(18)
C(1A)-C(2A)-C(6A)-C(5A)	179.17(14)
C(11A)-C(7A)-C(8A)-C(9A)	0.26(19)
C(7A)-C(8A)-C(9A)-C(10A)	-0.1(2)
C(8A)-C(9A)-C(10A)-C(11A)	-0.1(2)
C(9A)-C(10A)-C(11A)-C(7A)	0.22(19)
C(8A)-C(7A)-C(11A)-C(10A)	-0.29(1

References

1. G. M. Sheldrick, *Acta Cryst*. **2008**, *A64*, 112–122 and **2015**, *C71*, 3.
